# Supplementary material for: iFISH is a publically available resource enabling versatile DNA FISH to study genome architecture
Source: Nat Commun. 2019 Apr 9;10:1636. doi: 10.1038/s41467-019-09616-w (PMC6456570; doi:10.1038/s41467-019-09616-w)
Supplement: Supplementary file 1 — Supplementary Information [file 41467_2019_9616_MOESM1_ESM.pdf]

Supplementary Information

iFISH is a publically available resource enabling versatile DNA FISH to study genome architecture

Gelali et al.

Supplementary Figures

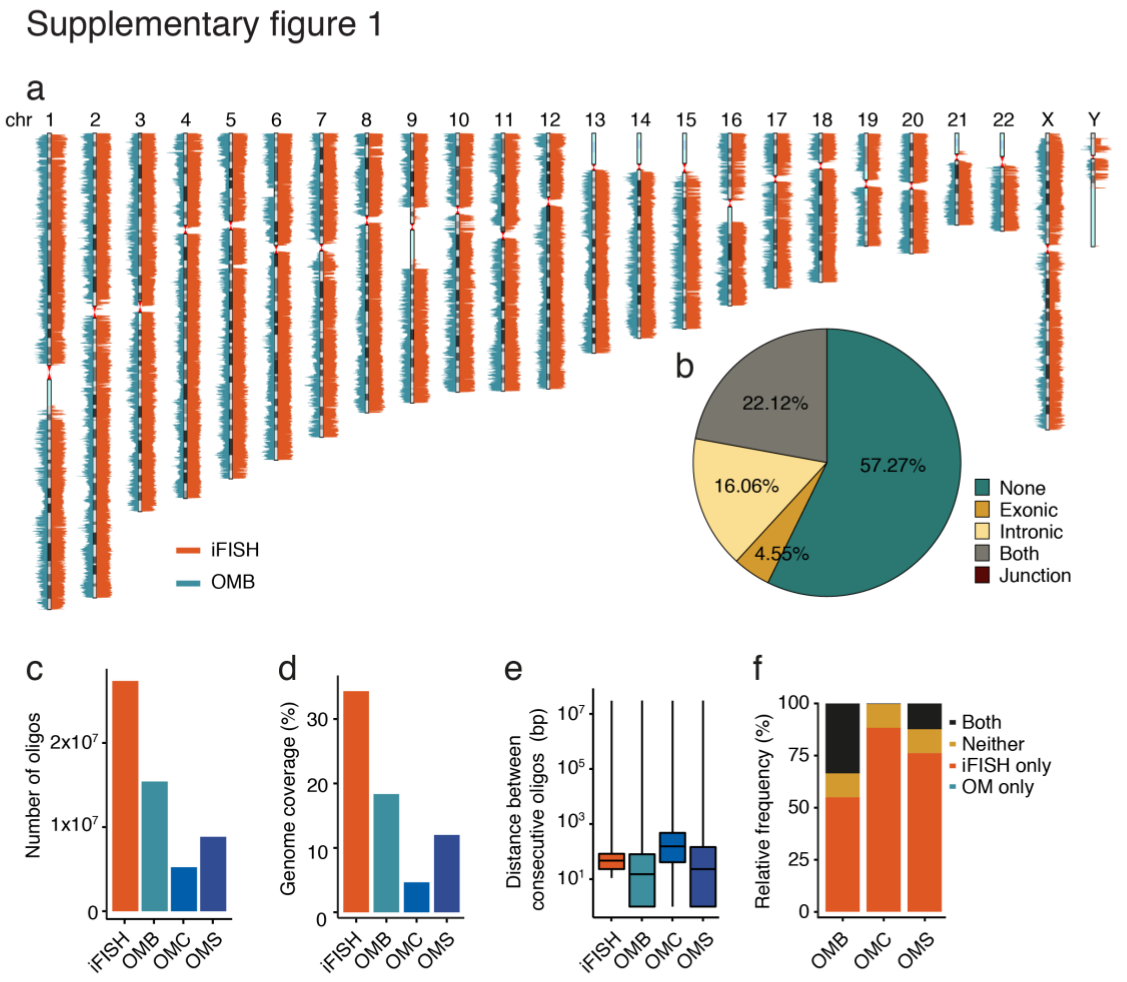

Supplementary Figure 1. (**a**) Number of oligos per consecutive (non-overlapping) 100 kb genomic windows, in the 40-mers database (iFISH) and OligoMiner 'Balanced' hg19 (OMB) oligo database. (**b**) Annotation of oligos in the 40-mers iFISH database. None, oligos with no overlap with transcribed regions in the human genome. Exonic, oligos overlapping with exons only. Intronic, oligos overlapping with introns only. Both, oligos overlapping with both exons and introns. Junction, oligos overlapping with exon-intron junctions. (**c**) Total number of oligos in the iFISH 40-mers database compared to the OligoMiner 'Balance' (OMB), 'Coverage' (OMC), and 'Stringent' (OMS) hg19 databases. (**d**) Same as in (c), for genome coverage. (**e**) Distributions of the distances in base-pairs (bp) between the consecutive targets of the oligos in the same databases as in (c) and (d). In all the box plots, the central line represents the median, the bottom and upper bounds of

the box represent the 25th and 75th percentile respectively, and the whiskers extend from –1.5×IQR to +1.5×IQR from the closest quartile, where IQR is the inter-quartile range. (**f**) Fraction of consecutive (non-overlapping) 15 kb genomic windows containing at least 96 oligos, found only in the iFISH 40-mers database (iFISH only), or only in the OligoMiner 'Balanced' hg19 (OMB) oligo database (OMB only), or in both or neither of them.

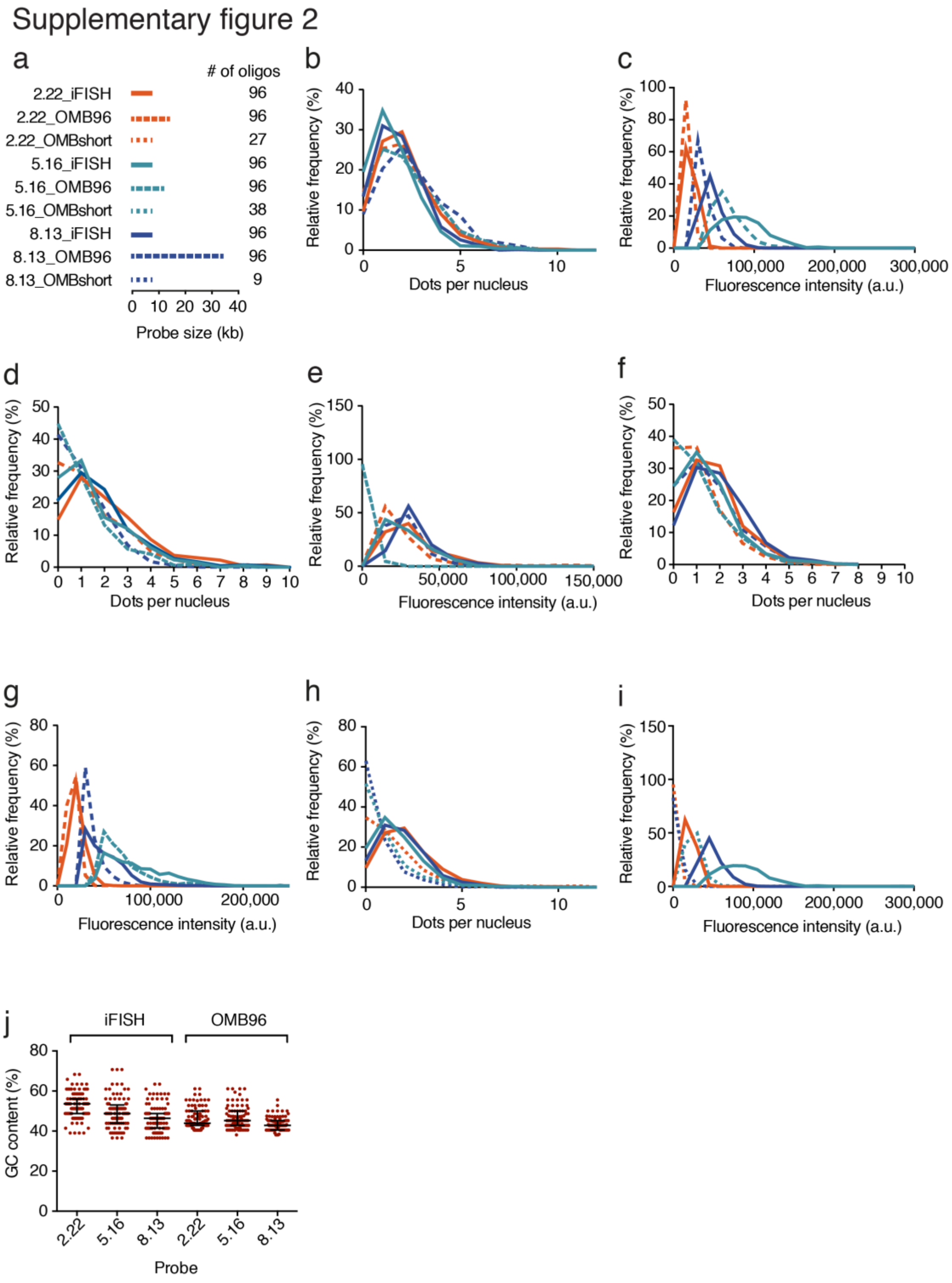

Supplementary Figure 2. (**a**) Schematic representation of the probe size, for nine different probes targeting three loci on chr2 (2.22), chr5 (5.16), and chr8 (8.13), designed either

using the iFISH 40mers database, or the OligoMiner 'Balanced' hg19 database (OMB). The number of oligo species in each probe is shown in the right column. (**b-i**) Distributions of dot counts per cell (b, d, f, h) and of mean dot intensities per cell (c, e, g, i), for the iFISH and OMB probes shown in (a), visualized in HAP1 cells. *n*, number of cells analyzed. The source data for the plots shown were obtained either by picking FISH dots using an automatic identification of a fluorescence intensity threshold that yields the distribution of dots per nucleus as similar to the expected one as possible (b-e and h-i) or by using the same intensity threshold for the corresponding iFISH and OMB probes (f-g). The source data for panels (d-e) were obtained following a DNA FISH procedure similar to the one described in [1] (for details, see "Comparison of iFISH and OMB probes" in the Methods). (**j**) GC content of the 96 oligos constituting the iFISH and OMB96 probes schematically shown in (a). Error bars indicate medians and interquartile ranges.

Supplementary figure 3

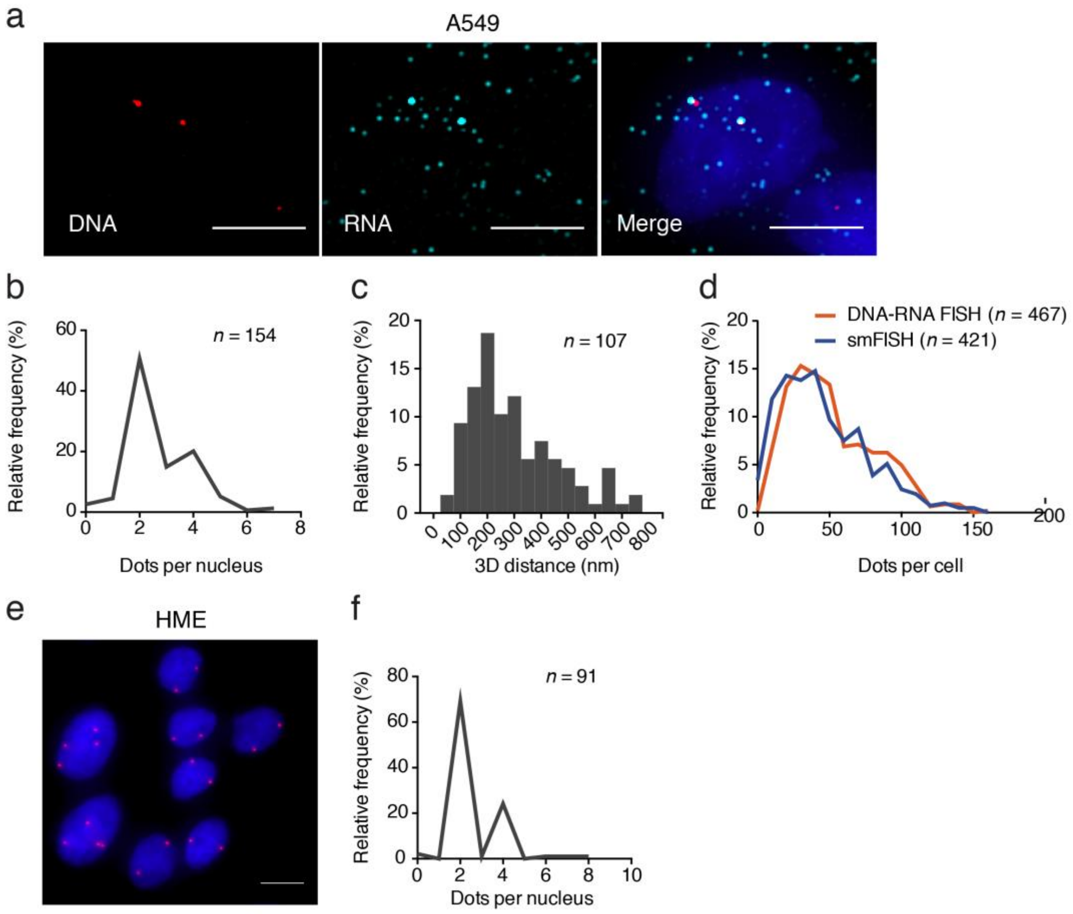

Supplementary Figure 3. (**a**) Simultaneous DNA-RNA FISH against *MYC* loci (red dots) and *MYC* transcripts (turquoise dots) in A549 cells. The white dots in the image on the right represent *MYC* loci being actively transcribed (transcription sites). Blue, DNA. Scale bar, 10 μm. (**b**) Distribution of the number of *MYC* DNA FISH dots per nucleus, in the images of which (f) is a representative example. *n*, number of cells analyzed. (**c**) Distribution of 3D distances between *MYC* loci and *MYC* transcription sites (TS), for 107 TS visually classified as true-positives in A549 cells. (**d**) Distribution of *MYC* transcript counts per cell, in A549 cells processed through smFISH only or simultaneoys DNA FISH-smFISH. *n*, number of cells analyzed. (**e**) iFISH visualization of a large (~1 Mb) region on chr8 encompassing the *MYC* locus, in HME cells. Red dots, *MYC* loci. Blue, DNA. Scale bar, 10 μm. (**f**) Distribution of the number of *MYC* DNA FISH dots per nucleus, in the

images of which (j) is a representative example. *n*, number of cells analyzed. All the microscopy images in this figure are the maximum intensity z-projection of each channel.

Supplementary figure 4

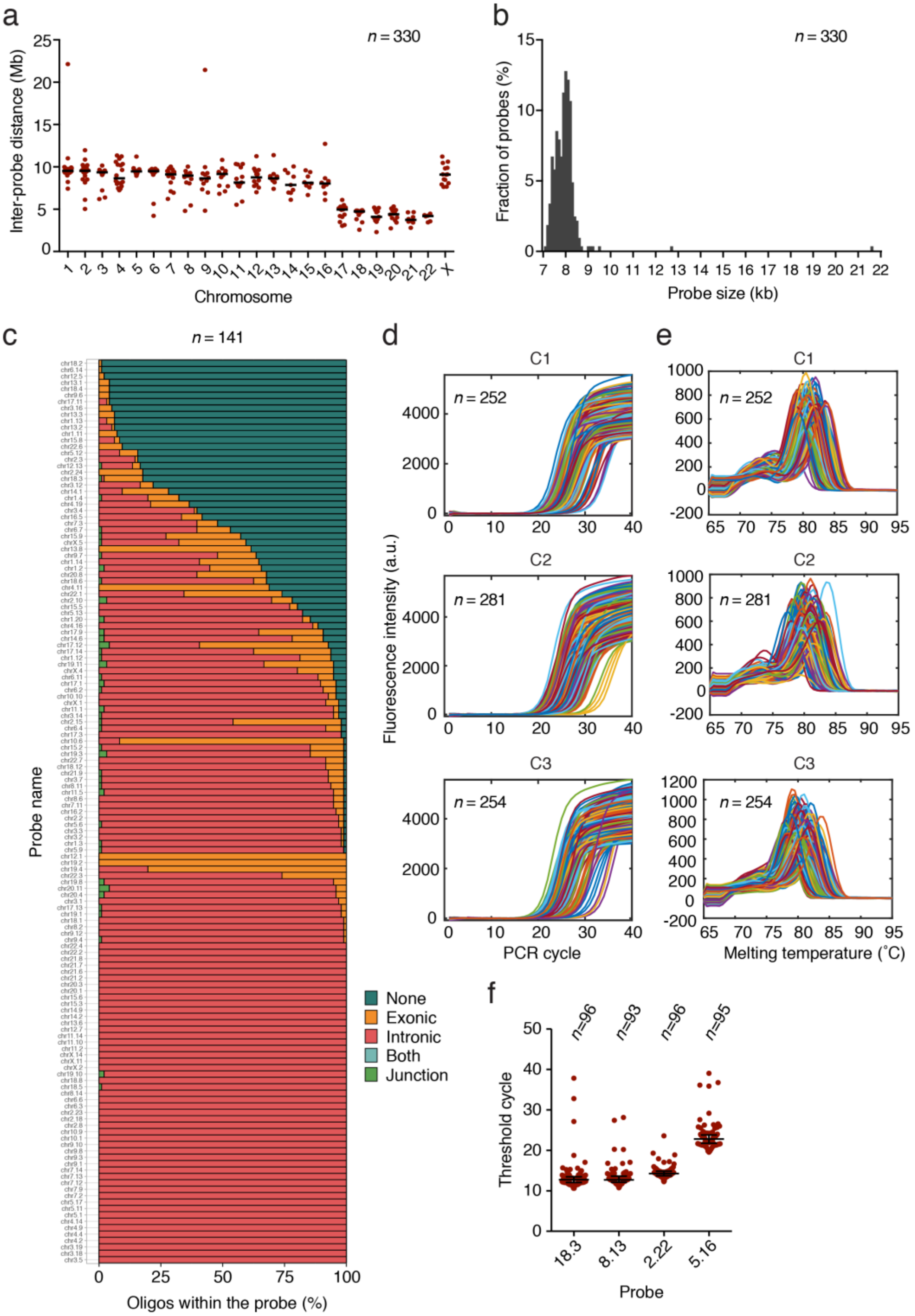

Supplementary Figure 4. (**a**) Distance between consecutive probes on the same chromosome, for the 330 probes shown in Fig. 1e. Black lines indicate mean values. The three outliers on chr1, 9 and 16 correspond to probe pairs across centromeric regions. (**b**) Distribution of the sizes of the 330 probes. Only two probes are larger than 10 kb. (**c**) Annotation of the oligos in 141 out of 330 probes (43%) that have at least one oligo that is complementary to an RNA sequence. None, oligos with no overlap with transcribed regions in the human genome. Exonic, oligos overlapping with exons only. Intronic, oligos overlapping with introns only. Both, oligos overlapping with both exons and introns. Junction, oligos overlapping with exon-intron junctions. When designing DNA FISH probes for simultaneous DNA-RNA FISH, if possible, avoid including oligos that are complementary to the transcribed strand in the targeted regions, as this might cause cross-talk between DNA and RNA FISH probes. (**d**) PCR amplification curves, for three color adapters C. *n*, number of probes (out of 330) there were successfully amplified in the first attempt. (**e**) Melting curves of the PCR amplifications shown in (e). Black lines indicate median values, error bars indicate medians and inter-quartile ranges. *n*, number of probes (out of 330) there were successfully amplified in the first attempt. (**f**) Threshold cycle for the oligos in the probes shown on the *x*-axis, amplified using real-time PCR. Each dot represents the mean threshold cycle of one oligo, measured in two or three replicates. *n*, number of oligos in each probe that were amplified in two or three replicates.

Supplementary figure 5

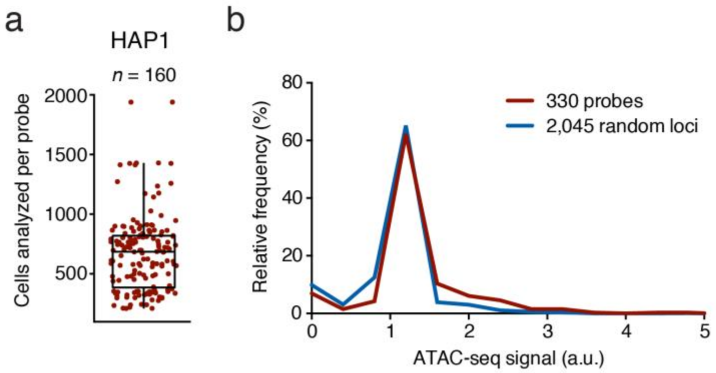

Supplementary Figure 5. (**a**) Number of HAP1 cells in which 153 out of the 330 probes shown in **Fig. 1e** were individually tested. Each dot corresponds to one probe visualized in one channel. *n*, number of unique probe-channel combinations. In the box plot, the central line represents the median, the bottom and upper bounds of the box represent the 25th and 75th percentile respectively, and the whiskers extend from –1.5×IQR to +1.5×IQR from the closest quartile, where IQR is the inter-quartile range. (**b**) Distribution of ATAC-seq signals within the genomic loci targeted by the 330 probes, and in 2,045 randomly picked, non-overlapping 10 kb regions, as control.

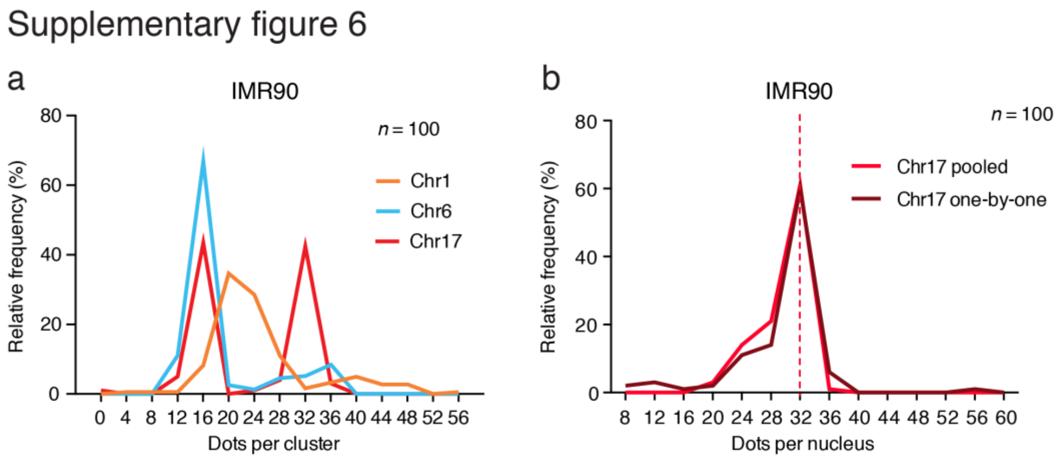

Supplementary Figure 6. (**a**) Distributions of dot counts per cluster per nucleus for three chromosomes, quantified in the images of which (a) is a representative example. Dots were manually grouped in either one or two clusters depending on how well separated were the clouds of dots. (**b**) Frequency distribution of the number of dots per nucleus using chr17 spotting probes produced in two different ways. In one case (pooled) all chr17 probes were pooled after the PCR step while in the other (one-by-one) every probe was produced separately and pooled only at the hybridization step. The dashed red line indicates the expected number of dots per nucleus.

Supplementary figure 7

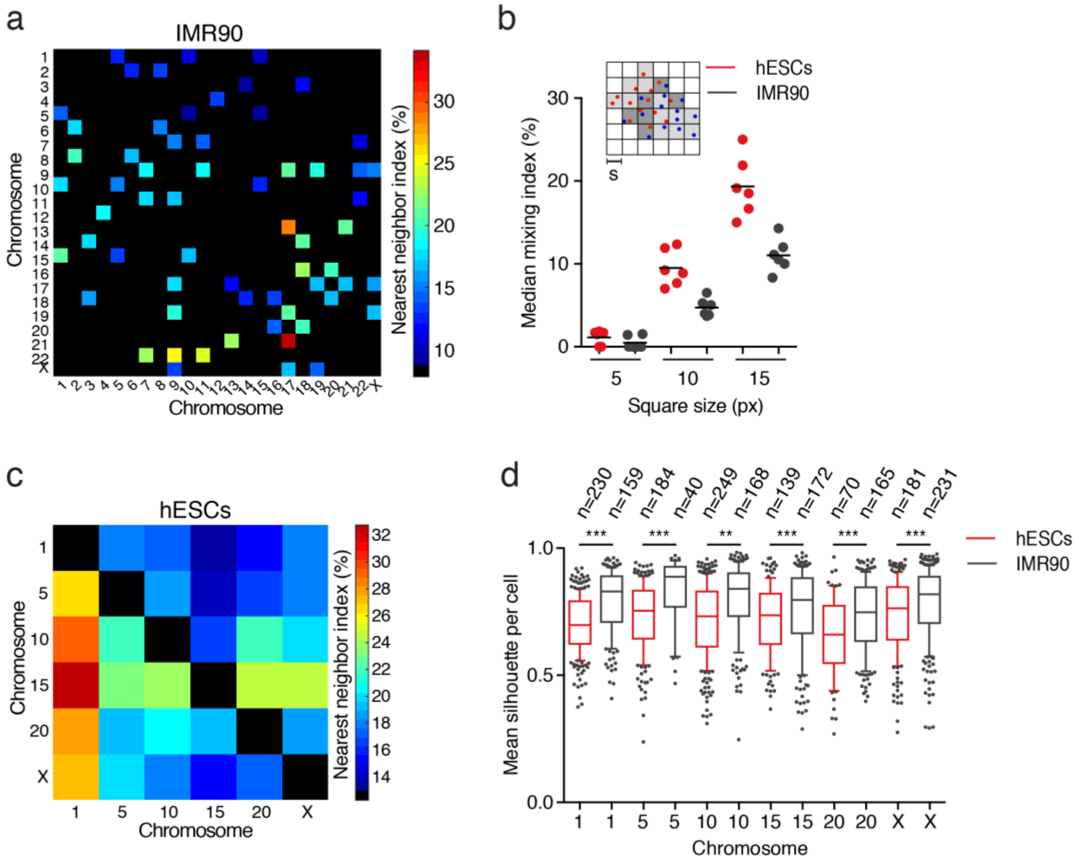

Supplementary Figure 7. (**a**) Average nearest neighbor index for 31 chromosome pairs visualized in IMR90 cells. Note that the matrix is not symmetric, as the index calculated for chrA *vs.* chrB is different than the index calculated for chrA *vs.* chrB (**b**) Mixing index between the same six chromosome pairs shown in Fig. 4g, calculated using three different square sizes (*s*). Each dot represents the median value of the mixing index for one chromosome pair. Black lines, mean values. Px, pixels. (**c**) Same as in (a), but for 15 chromosome pairs visualized in hESCs. (**d**) Distribution of silhouette values per cell obtained after applying K-means clustering to partition into two clusters all the dots belonging to the same chromosome, in hESCs *vs.* IMR90 cells. *n*, number of cells analyzed. ***, Mann-Whitney test *P* value $\leq$ 0.0005. **, Mann-Whitney test *P* value $\leq$ 0.005. In all the box plots, the central line represents the median, the bottom and upper bounds of the box represent the 25th and 75th percentile respectively, and the whiskers extend from the 5th to the 95th percentile.

Supplementary Tables

Supplementary Table 1. List of dyes tested and used.

Dyes tested	Dyes selected	Site of oligo labelling	Excitation filter	Dichroic mirror	Emmision filter
Hoechst 33342	Hoechst 33342	NA	390/22 (Lumencor)	440/40 (Custom-made Polychroic 1 from Chroma)	FF01-447/60-25 (Semrock)
Alexa Fluor 488	Alexa Fluor 488	3' and 5'	FF01-494/20-25 (Semrock)	535/30 (Custom-made Polychroic 2 from Chroma)	FF01-542/27-25 (Semrock)
ATTO 488					
Alexa Fluor 546					
Alexa Fluor 555					
ATTO 542	ATTO 542	3'	FF01-534/20-25 (Semrock)	570/20 (Custom-made Polychroic 1 from Chroma)	FF01-567/15-25 (Semrock)
Alexa Fluor 594	Alexa Fluor 594	3' and 5'	FF01-586/20-25x5 (Semrock)	630/27 (Custom-made Polychroic 2 from Chroma)	FF01-628/32-25 (Semrock)
ATTO 594					
Alexa Fluor 647					
ATTO 647N	ATTO 647N	3'	FF01-628/32-25 (Semrock)	675/29 (Custom-made Polychroic 1 from Chroma)	FF01-676/29-25 (Semrock)
Alexa Fluor 700	Alexa Fluor 700	3'	ZET690/10x (Chroma)	720dcxxr (Chroma)	ET740/40x (Chroma)
ATTO 700					
ATTO 725					
DY-700					
DY730					
Promo Fluor700					
Alexa Fluor 790					
IRDye 800CW	IRDye 800CW	3' and 5'	780 nm MaxLine® laser clean-up filter (Semrock)	FF801-Di02-25x36	FF01-832/37-25 (Semrock)

Supplementary Table 2. List of microscope components used.

Microscope component	Manufacturer
TI-E Inverted Microscope	NIKON
TI-ND6-PFS-S Perfect Focus Unit Motorized Nosepiece	NIKON
Objective CFI Plan Fluor DLL 10X	NIKON
Objective CFI Plan Apokromat 60X λ oil	NIKON
Objective CFI Plan Apokromat 100X λ oil	NIKON
TI-C System Condenser Turret	NIKON
TI-C-LWD LWD Lens Unit for System Condenser Turret	NIKON
TI-S-E Motorized stage	NIKON
TI-SH-U Universal Holder	NIKON
Software NIS-Elements AR including the NIS-A Bundle JOBS	NIKON
iXon Ultra 888 USB3 Camera	ANDOR
Piezo Insert W/USB Controller, Nano-Z100	Mad City Labs Inc.
98 x 66 cm Active Anti-vibration Table	SUPERTECH
Cage Incubator with a temperature unit	OKOLAB

Supplementary Table 3. List of light sources used.

Spectra X Light Engine 1: violet (LED), blue (LED), cyan (LED), green-yellow (LED), red (LED), Near-IR (LED)
Spectra X Light Engine 2: violet (LED), cyan (the original Spectra X cyan light source replaced by the cyan light source from the SOLA SE 365 FISH), green-yellow (the original Spectra X green-yellow light source replaced by the green-yellow light source from the SOLA SE 365 FISH), red (LED), Near-IR1 (690 nm laser diode), Near-IR2 (780 nm laser diode)

Supplementary Notes

Supplementary Note 1. Comparison with the protocol for producing MERFISH probes. The iFISH probe production step-by-step protocol described in the previous section builds on the previously published protocol for synthesizing MERFISH probes[2], with two major improvements. Firstly, we optimized PCR conditions so that the cost per reaction is lower compared to MERFISH (see table below), and a lower amount of oligo-pool can be used as template in the same PCR volume (20 fmol in iFISH *vs.* 50 fmol in MERFISH). This means that, with the iFISH protocol, the same oligopool can be used to perform 2.5 times more PCR-IVT-RT cycles (*i.e.*, to re-synthesize the same probe(s) multiple times from the same oligo-pool) at the same initial cost for purchasing the oligo-pool.

	iFISH	MERFISH
**Oligopool input in PCR (fmol)**	20	50
**PCR cost (USD)***	2.37	4.89
**IVT cost (USD) ****	18.57§	15.24
**RT cost (USD) *****	65.27	65,27
**Total cost for converting all the**	86.21	85.40

*The final PCR reaction volume is set to be the same in all the methods
**Based on the volumes shown in the step-by-step protocol in section 3, two IVT reactions can be performed for every PCR reaction
***Based on the volumes shown in the step-by-step protocol in section 3, five RT reactions can be performed for every IVT reaction
§Cost including RNA purification using RNAClean XP beads and RNA concentration measurement using Qubit.

Secondly, we introduced an RNA purification step, before RT, that allows measuring the concentration of RNA, and thus using the same amount of RNA as RT input, for every probe synthesized in parallel. This is critical to make the final ssDNA yield reproducible across multiple probes synthesized in parallel, as well as across different experiments. Purifying RNA before RT also allows identifying samples in which the IVT reaction, for some reasons, has failed, thus avoiding to proceed to RT. Furthermore, by measuring the RNA concentration prior to RT, one can use the maximum possible amount of RNA input in the reaction, thus maximizing cost-effectiveness. For example, as shown in the plot below, since max 5 μg of RNA can be used as input per RT reaction, in both the iFISH and MERFISH protocols, then the cost of producing the same final amount of ssDNA becomes higher when less than 5 μg is used. On the other hand, if more than 5 μg is used, a fraction of the RNA cannot be converted to ssDNA and is therefore wasted in the process, resulting in extra cost with no benefit. Importantly, even though the addition of an RNA purification step slightly increases the cost of the IVT step (see table above), the combined cost of PCR,

IVT and RT reactions is only minimally higher (0.95%) in iFISH protocol compared to MERFISH.

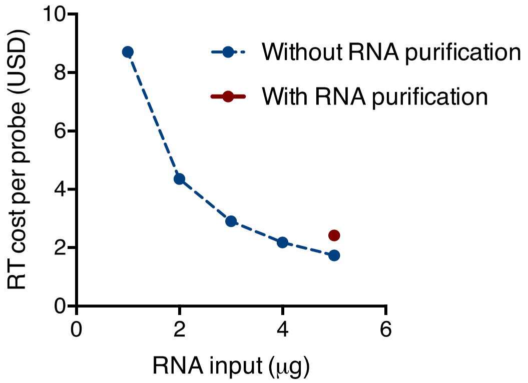

Supplementary Note 2. Considerations on the cost of the iFISH probe repository. In this work, we have established a repository of more than 1,000 oligo-based DNA FISH probes, targeting more than 350 loci all over the human genome, which we are continuously expanding to include more probes targeting the human genome. Here, we present an analysis of the reagent costs needed to build and maintain an even larger repository of 3,000 DNA FISH probes, each in six different colors, targeting 3,000 non-overlapping 1 Mb loci all along the human genome, with each probe consisting of 96 oligo species (as shown in this paper, this number of oligos per probe is sufficient to visualize a locus of less than 15 kb with high sensitivity and specificity in cultured interphase cells). This gives $3{,}000 \times 96 =$ 288,000 oligos to be synthesized on $288{,}000 / 12{,}000 = 24$ arrays, for a total cost of 1,636 USD $\times$ 24 = 39,264 USD (approximate cost of one oligo-pool based on CustomArray price for 12K arrays of 100 nt oligos). Now, let's consider the case in which we amplify all the 24 oligo-pools by PCR, generating six different versions of each probe by using six different C adapters. Using 96-well plates (one well per probe, one plate per C adapter), we need to perform $3{,}000 \times 6 = 18{,}000$ PCR reactions in 188 plates. A set of 30 F and 30 R primers synthesized at 25 nmol synthesis scale in 96-well plates is completely sufficient to amplify each oligopool separately, and can be purchased for 792 USD (approximate cost based on Integrated DNA Technologies prices for oligos synthesized at 25 nmol scale in 96-well plates). Using the reagents listed in the iFISH step-by-step protocol (see above), 27,754 USD are needed to amplify all the 24 oligopools and

to purify each PCR product using magnetic beads. This gives a total setup cost of 67,810 USD to establish a repertoire of 18,000 individual PCR products, which can be stocked long-term at –20°C and made available to the research community, following the Addgene model for plasmids. This would allow researchers to quickly synthesize probes against virtually any genomic region of interest in-house, simply by purchasing IVT and RT reagents, and without the need to design and purchase oligo-pools. In this context, the cost-effectiveness of the iFISH protocol over MERFISH, which we discussed above, becomes even more evident. For example, let us assume to re-generate the aforementioned 18,000 probes as PCR products, whenever the oligo-pools are finished. As it can be appreciated in the plot below, the cumulative cost using the iFISH protocol grows at a much lower rate compared to a scenario in which the probes are being produced following the MERFISH protocol.

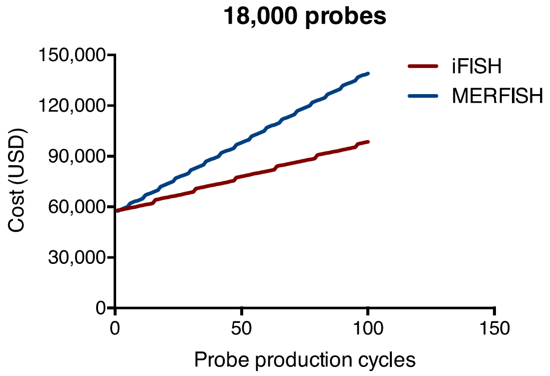

Using the iFISH protocol, all of the 18,000 probes can be produced 100 times for less than 100,000 USD. This means that each of the 18,000 probes could be distributed to 100 different research groups at a nominal cost of less than 6 USD per probe. Accounting for personnel and other indirect costs, we estimate that iFISH probes could be distributed as ready-to-use PCR products for less than 10 USD per probe (excluding shipment costs), which is an order of magnitude lower than the price of probes that can be purchased from commercial vendors.

Supplementary References

1. Nir, G. *et al.* Walking along chromosomes with super-resolution imaging, contact maps, and integrative modeling. *PLoS Genet.* **14**, e1007872 (2018).
2. Moffitt, J. R. & Zhuang, X. RNA Imaging with Multiplexed Error-Robust Fluorescence In Situ Hybridization (MERFISH). *Methods Enzymol.* **572**, 1–49 (2016).
