# Supplementary material for: iFISH is a publically available resource enabling versatile DNA FISH to study genome architecture
Source: Nat Commun. 2019 Apr 9;10:1636. doi: 10.1038/s41467-019-09616-w (PMC6456570; doi:10.1038/s41467-019-09616-w)
Supplement: Supplementary file 13 — Reporting Summary [file 41467_2019_9616_MOESM13_ESM.pdf]

nature research

Corresponding author(s): Bienko M, Crosetto N

Last updated by author(s): Mar 15, 2019

Reporting Summary

Nature Research wishes to improve the reproducibility of the work that we publish. This form provides structure for consistency and transparency in reporting. For further information on Nature Research policies, see Authors & Referees and the Editorial Policy Checklist.

Statistics

For all statistical analyses, confirm that the following items are present in the figure legend, table legend, main text, or Methods section.

n/a	Confirmed	
☐	☒	The exact sample size (*n*) for each experimental group/condition, given as a discrete number and unit of measurement
☒	☐	A statement on whether measurements were taken from distinct samples or whether the same sample was measured repeatedly
☐	☒	The statistical test(s) used AND whether they are one- or two-sided<br>*Only common tests should be described solely by name; describe more complex techniques in the Methods section.*
☒	☐	A description of all covariates tested
☒	☐	A description of any assumptions or corrections, such as tests of normality and adjustment for multiple comparisons
☐	☒	A full description of the statistical parameters including central tendency (e.g. means) or other basic estimates (e.g. regression coefficient) AND variation (e.g. standard deviation) or associated estimates of uncertainty (e.g. confidence intervals)
☐	☒	For null hypothesis testing, the test statistic (e.g. *F*, *t*, *r*) with confidence intervals, effect sizes, degrees of freedom and *P* value noted<br>*Give P values as exact values whenever suitable.*
☒	☐	For Bayesian analysis, information on the choice of priors and Markov chain Monte Carlo settings
☒	☐	For hierarchical and complex designs, identification of the appropriate level for tests and full reporting of outcomes
☐	☒	Estimates of effect sizes (e.g. Cohen's *d*, Pearson's *r*), indicating how they were calculated

Our web collection on statistics for biologists contains articles on many of the points above.

Software and code

Policy information about availability of computer code

Data collection	NIS Elements software (Nikon) was used for image acquisition
Data analysis	For the design of the oligo sequences, we used a custom perl pipeline exploiting JELLYFISH v2.2.6, VMATCH v2.2.4 and OligoArrayAux v3.8 for designing the target sequences as described in the text. We used a custom-made pipeline (OOD-FISH v0.0.2) to design the F, R and C sequences, which includes: BLAT v36x1 to align to the reference genome as described in the text, customized R/C99 scripts to calculate the self/hetero-dimerization free energy, as well as the Parallel Maximum Clique library to find the largest subset of non interacting sequences. All scripts for the F/R/C sequence design are available at https://github.com/ggirelli/ood-fish/releases/tag/v0.0.2.<br>For probe design, we used a Python3 web interface (iFISH-probe-design v2.0.2; d.o.i: 10.5281/zenodo.2565789) that we developed. The iFISH4U website is run using the ifpd_serve script form the iFISH-probe-design repository, extending it with the code available at https://github.com/ggirelli/iFISH4U.<br>For image analysis, we used our in-house suite DOTTER, written in MATLAB (MATLAB and Statistics Toolbox Release R2018a) and C99 with GSL (https://www.gnu.org/software/gsl/). K-means and silhouette MATLAB functions were used for dot clustering and for computation of the mean silhouette value per cell of the clustered dots.<br>We performed further downstream analysis using custom R scripts, and used the ggplot2 package to generate the plots.

For manuscripts utilizing custom algorithms or software that are central to the research but not yet described in published literature, software must be made available to editors/reviewers. We strongly encourage code deposition in a community repository (e.g. GitHub). See the Nature Research guidelines for submitting code & software for further information.

Data

Policy information about availability of data

All manuscripts must include a data availability statement. This statement should provide the following information, where applicable:

- Accession codes, unique identifiers, or web links for publicly available datasets
- A list of figures that have associated raw data
- A description of any restrictions on data availability

We have added a Data Availability statement.

Field-specific reporting

Please select the one below that is the best fit for your research. If you are not sure, read the appropriate sections before making your selection.

☒ Life sciences ☐ Behavioural & social sciences ☐ Ecological, evolutionary & environmental sciences

For a reference copy of the document with all sections, see nature.com/documents/nr-reporting-summary-flat.pdf

Life sciences study design

All studies must disclose on these points even when the disclosure is negative.

Sample size	All sample sizes are described in the relevant figures.
Data exclusions	NA
Replication	Due to the nature of FISH assays, we obtained a large collection of data by imaging the same set of samples multiple times, in order to image at high magnification a large number of single cells.
Randomization	NA
Blinding	NA

Reporting for specific materials, systems and methods

We require information from authors about some types of materials, experimental systems and methods used in many studies. Here, indicate whether each material, system or method listed is relevant to your study. If you are not sure if a list item applies to your research, read the appropriate section before selecting a response.

Materials & experimental systems

n/a	Involved in the study	
☒	☐	Antibodies
☐	☒	Eukaryotic cell lines
☒	☐	Palaeontology
☒	☐	Animals and other organisms
☒	☐	Human research participants
☒	☐	Clinical data

Methods

n/a	Involved in the study	
☒	☐	ChIP-seq
☒	☐	Flow cytometry
☒	☐	MRI-based neuroimaging

Eukaryotic cell lines

Policy information about cell lines

Cell line source(s)	IMR90, A549, HME from ATCC® (cat. no. CCL-186, CCL-185, and PCS-600-010, respectively)<br>HAP1 from Horizon Discovery (cat.no. C859)<br>Human embryonic stem cells HS975 previously derived as described in: Rodin et al., Nature Communications volume 5, Article number: 3195 (2014)
Authentication	None of the cell lines used were authenticated
Mycoplasma contamination	Our cell lines are regularly tested for mycoplasma contamination.
Commonly misidentified lines (See ICLAC register)	None used
