# Supplementary material for: iFISH is a publically available resource enabling versatile DNA FISH to study genome architecture
Source: Nat Commun. 2019 Apr 9;10:1636. doi: 10.1038/s41467-019-09616-w (PMC6456570; doi:10.1038/s41467-019-09616-w)
Supplement: Supplementary file 15 — Supplementary Software [file 41467_2019_9616_MOESM15_ESM.zip › Supplementary Software/documentation/oligo-picker-usersGuide.pdf]

Identification of Unique Oligonucleotides: Users Guide

Mihaela Martis

October 27, 2016

0.1 Getting started

The pipeline detects unique DNA oligonucleotides in the human genome using a combination of external tools and internal filtering steps. The identified oligomers are selected based on the following criteria: uniqueness of the genomic location, melting temperature, GC content, and inability to form a secondary structure. The unique oligomers are detected using the Jellyfish (version 2.2.3) software, while Vmatch (version 2.2.4) return their genome/chromosom positions. Vmatch runs twice, first to determine if the selected oligos are unique over the entire length, second by allowing up to 5 mismatches. The second run allows a much more restrictive filtering of the oligonucleotides. Nevertheless, the number of allowed mismatches should not exceed 5. Due to the intensive search necessary to perform the task, the allocated core hours and time would be exceeded and the pipeline will fail.

```
vmatch -q in.fa -l 60 -p -d -selfun mydb.so -showdesc 100 index > out.fa
vmatch -q in.fa -complete -p -d -h 9b -selfun mydb.so -showdesc 100 index > out.fa
```

The degree of free energy is calculated by the OligoArrayAux software (version 3.8). All oligomers with an GC-content and melting temperature below or above the given intervals, and with a ΔG (free energy) < 0 are filtered out from the final data set. In addition the oligomers containing homopolymers (e.x. AAAAA or TTTTT or GGGG or CCCC) can be droped out, too. The melting temperature is calculated using the following formula:

$$81.5 + (0.41 * GC) + (16.6 * log10(Na)) - (500/K) - (0.62 * F),$$

where

GC = %GC-content

Na = salt concentration

F = formamid concentration

K = k-mer size

The unique oligomers are uploaded to a SQLite database, which can be enquired using the same pipeline (see 0.3) or by using the SQLite Manager add-on for the Firefox-browser (http://www.sqlabs.com/sqlitemanager.php). For each run the data will be saved in a table, which name will contain the following parameters: kmer (-k), minimal and maximal GC content (-g and -G), reference name (-n), maximal overlap between 2 oligomers (-v), the delta needed to increase or decrease the melting temperature, as well as the information if a homopolymer filtering has been done or not (-F) (e. x. *kmer60_dtm10_gcmin35_gcmax80_hpolno*). The table will be overwritten with each run, except one of the above mentioned parameters are changed.

The pipeline automatically generates a log file for each individual chromosom to describe step by step the design process. The main interest of this files are the explanations of why the design failed for a given step. This can help to figure out if a parameter was too stringent and need to be relaxed for a second run or if other events occured, which caused the pipelinie to crash.

Furthermore, the files .bash_profile and .bashrc located in the home directory need to be customized. This is necessary, because Vmatch, OligoArrayAux and

some Perl libraries required by the pipeline, could be installed only locally. Please contact me (mihaela.martis@bils.se) to get further information.

0.2 Options

This section describes the command line options to run the pipeline in order to identify unique oligomers.

-a, --all	This option accepts only yes or no as arguments and it is mandatory. It specifies if the pipeline should perform only a database enquiry or the identification of unique k-mers.
-b, --begin_pos	The start position of the region of interest. This value is necessary if unique oligomers from a certain region should be extracted from the database. This value is optional.
-c, --chromosom	The chromosom of the region of iterest (e.x. 1, 8, X, or Y). This value is optional.
-d, --db_name	The path and name of the SQLite database. If this option is not used, then the default path is equal to the working directory and the database name is unique_oligonucleotides.db
-D, --distance	The minimum distance between two unique oligomers. If this option is not used, then the default value is 10.
-e, --end_pos	The stop position of the region of interest. This value is necessary if unique oligomers from a certain region should be extracted from the database. This value is optional.
-f, --formamid	The percentage of the formamid concentration. If this option is not used, then the default value is 50.
-F, --hpol_flag	This option accepts only 'yes' or 'no' as arguments. 'yes' means that homopolymers were filtered in the table of interest, 'no' means there was no homopolymer filtering done. It is mandatory if you want to run only the database enquiry.
-g, --gc_min	The minimum oligomer GC content in percentage. A positive integer between 0 and 100 is expected. If this option is not used, then the default value is 35.
-G, --gc_max	The maximum oligomer GC content in percentage. A positive integer between 0 and 100 is expected. If this option is not used, then the default value is 80.
-h, --help	Show a summary of all options available for this pipeline and terminate the pipeline.
-k, --kmer	The length of the unique oligomers. This option is mandatory and should be a positive integer between 20 and 100.
-m, --mail	The email address to which the SLURM system should report if the jobs fail or complete. Please use quotation marks (' ') around the email address. [default: 'mihaela.martis@bils.se'].
-M, --mismatch	The number of allowed mismatches in the second Vmatch run. The number should be not higher than 5, due to runtime issues.
-n, --refname	The name of the reference genome. [default: human]

-o, --homopolymer	A list of homopolymers separated by semi-colon, which should not be contained in the oligomer sequences (e.x. GGGG;CCCC;AAAAA;TTTTT). If this option is not used, then the default value is na (no homopolymer filtering).
-p, --project	Uppmax project name. If this option is not used, then the default value is b2015233.
-r, --reference	The path to the individual human chromosomes. If this option is not used, then the default is /sw/data/uppnex/reference/Homo_sapiens/hg19/chromosomes
-s, --salt	The salt concentration [Na+] in M. If this option is not used, then the default value is 0.42.
-S, --ss_temp	The threshold temperature for the calculation of the free energy of the secondary structure. If this option is not used, then the default value is 65.
-t, --tm_delta	The degree of which the average melting temperature should be increased and decreased to form an interval. A positive integer is expected. If this option is not used, then the default value is 10.
-u, --user	UPPMAX user id.
-v, --maxoverlap	The maximal overlap between 2 oligomers in percentage.
-w, --workdir	The absolute path to the working directory. If this option is not used, then the default working directory is: /proj/b2015233/nobackup/uniqueOligoPipeline.
-W, --wgs	The absolute path to the whole genome sequence of interest. The default directory is: /sw/data/uppnex/igenomes/Homo_sapiens/Ensembl/GRCh37/Sequence/WholeGenomeFasta/genome.fa.

0.3 How to

The pipeline needs to be run from the command line by using various options described above (see 0.2). There are three ways to run the pipeline:

- only to enquiry the database for unique oligomers from a certain region on a given chromosome. This presupposes that the unique kmers were already identified and stored in the database. In this case the options -a, -k, -c, -b, -e, and -F are mandatory:

  perl uniqueDnaOligonucleotidesCatcher.pl -a no -k 60 -c 10 -b 15000 -e 30000 -F yes

  perl uniqueDnaOligonucleotidesCatcher.pl -a no -k 60 -c 10 -b 15000 -e 30000 -F no

  The -F option specifies whether a homopolymer filtering has been performed or not.

- to identify unique oligomers for a given k-mer size without database enquiry. In this case only the options -a and -k are mandatory:

perl uniqueDnaOligonucleotidesCatcher.pl -a yes -k 60

- to identify unique oligomers for a given k-mer size and enquiry the database for a certain region:

  perl uniqueDnaOligonucleotidesCatcher.pl -a yes -k 60 -c 10 -b 15000 -e 30000

  In this case the -F option is not required. Whether a homopolymer filtering occured or not is deduced automatically.

If oligomers containing homopolymers should be excluded from the final data set, too, then the following cue can be used:

perl uniqueDnaOligonucleotidesCatcher.pl -a yes -k 60 -o 'AAAAA;TTTTT;CCCC;GGGG'. Here, the quotation marks (' ') are very important and should not be forgotten.
