# Supplementary material for: iFISH is a publically available resource enabling versatile DNA FISH to study genome architecture
Source: Nat Commun. 2019 Apr 9;10:1636. doi: 10.1038/s41467-019-09616-w (PMC6456570; doi:10.1038/s41467-019-09616-w)
Supplement: Supplementary file 16 — Description of Additional Supplementary Files [file 41467_2019_9616_MOESM16_ESM.docx]

**Supplementary Data Legends**

**Supplementary Data 1.** Summary of oligo design features in previously described Oligopaint probes.

**Supplementary Data 2.** Sequences of the oligos in the three probes designed to compare the iFISH and OM databases.

**Supplementary Data 3.** Genomic coordinates and sequences of the oligos in the MYC DNA and RNA FISH probes.

**Supplementary Data 4.** Genomic coordinates and sequences of the oligos in the large MYC probe.

**Supplementary Data 5.** Genomic coordinates and sizes of the 330 probes.

**Supplementary Data 6.** Sequences of the oligos in the 330 probes.

**Supplementary Data 7.** Sequences of the PCR primers used to generate the 330 probes.

**Supplementary Data 8.** Sequences of non-cross-hybridizing 20-mers orthogonal to the human genome.

**Supplementary Data 9.** Sequences of the primers used to assess the oligo complexity in four probes randomly selected from the 330 probes.

**Supplementary Data 10.** Genomic coordinates and sizes of the probes used to generate the chr17-spotting 'dense' probe.

**Supplementary Data 11.** Sequences of the oligos in the 63 probes used to generate the 'dense' chr17-spotting probe.

**Supplemetnary Software:** Software for designing the 40-mers database, the probe design pipeline, and the iFISH4U web interface
