# Supplementary material for: iFISH is a publically available resource enabling versatile DNA FISH to study genome architecture
Source: Nat Commun. 2019 Apr 9;10:1636. doi: 10.1038/s41467-019-09616-w (PMC6456570; doi:10.1038/s41467-019-09616-w)
Supplement: Supplementary file 15 — Supplementary Software [file 41467_2019_9616_MOESM15_ESM.zip › Supplementary Software/documentation/iFISH-probe-design-gh-pages.zip!/iFISH-probe-design-gh-pages/_layouts/default.html]

{{ site.title }}


# {{ site.title }}

{{ site.description }}

 View the Project on GitHub  
 View the Project on PyPI  
{{ site.github.owner\_name }}/{{ site.github.repository\_name }} ({{ site.version }})

#### Content

- Introduction
  - Database
  - Algorithms
- Install
- Usage
  - Scripts
  - Interface
  - Examples
- Contributing
- Code of Conduct

# {{ page.title }}

{{ content }}

This project is maintained by

Hosted on GitHub Pages — Theme based on minimal
