# Supplementary material for: iFISH is a publically available resource enabling versatile DNA FISH to study genome architecture
Source: Nat Commun. 2019 Apr 9;10:1636. doi: 10.1038/s41467-019-09616-w (PMC6456570; doi:10.1038/s41467-019-09616-w)
Supplement: Supplementary file 15 — Supplementary Software [file 41467_2019_9616_MOESM15_ESM.zip › Supplementary Software/source-code/oligo-picker.zip!/genome_v0.3/docs/vmatch.pdf]

The *Vmatch* large scale sequence analysis software

A Manual

Stefan Kurtz
Center for Bioinformatics,
University of Hamburg,
Bundesstrasse 43, 20146 Hamburg, Germany,
E-mail: kurtz@zbh.uni-hamburg.de

August 4, 2014

Contents

1 Introduction **1**

2 `mkvtree` **4**

2.1 The Options of `mkvtree` 7

2.2 Applying `mkvtree` 11

3 `mkdna6idx` **15**

4 `vseqinfo` **17**

4.1 Applying `vseqinfo` 17

5 `vstree2tex` **18**

5.1 Applying `vstree2tex` 19

6 `vseqselect` **20**

6.1 Applying `vseqselect` 21

7 `vsubseqselect` **22**

7.1 Applying `vsubseqselect` 23

8 `vmigrate.sh` **24**

8.1 Applying `vmigrate.sh` 24

9 `vmatch` **24**

9.1 Input Options 27

9.2 Matchkind Options 27

9.3 Postprocessing Options 29

9.4 Algorithm Options 33

9.5 Direction Options 34

9.6 Matchconstraint Options 34

9.7 Output Options 37

9.8 Miscellaneous Options 39

9.9 Applying `vmatch` 40

9.9.1 Self Comparison 40
9.9.2 Matching Queries against an Index 48
9.9.3 Matching a DNA sequence against a Protein Index 53
9.9.4 Matching a DNA sequence on the Protein Level 53
9.9.5 Computing Regions not containing a Match 55
9.9.6 Masking Matches 56
9.9.7 Clustering Indexed Sequences 57
9.9.8 Computing a non-redundant set of sequences 59
9.9.9 Chaining Matches 60
9.9.10 Clustering Matches 61
9.9.11 Selection Functions 63

10 `vmatchselect` **65**

11 Recent Changes **66**

12 References **72**

A Basic Notions **74**

B Specification of a symbol mapping **76**

C The X-Drop Extension Strategy **78**

D Substring Specifications **78**

E Selection Function Bundles **79**

F The Tables Comprising the Index **82**

G Environment Variables **86**

1 Introduction

This document describes *Vmatch*, a versatile software tool for efficiently solving large scale sequence matching tasks. *Vmatch* subsumes the software tool *REPuter* [7] but is much more general, with a very flexible user interface, and improved space and time requirement. Here are the most important features of *Vmatch*.

Persistent index

Usually, in a large scale matching problem, extensive portions of the sequences under consideration are static, i.e. they do not change much over time. Therefore it makes sense to preprocess this static data to extract information from it and to store this in a structured manner, allowing efficient searches. *Vmatch* does exactly this: it preprocesses a set of sequences into an index structure. This is stored as a collection of several files constituting the persistent index. The index efficiently represents all substrings of the preprocessed sequences and, unlike many other sequence comparison tools, allows matching tasks to be solved in time, *independent* of the size of the index. Different matching tasks require different parts of the index, but only the required parts of the index are accessed during the matching process.

Alphabet independency

Most software tools for sequence analysis are restricted to DNA and/or protein sequences. In contrast, *Vmatch* can process sequences over any user defined alphabet not larger than 250 symbols. *Vmatch* fully implements the concept of *symbol mappings*, denoting alphabet transformations. These allow the user to specify that different characters in the input sequences should be considered identical in the matching process. This feature is used to group similar amino acids, for example.

Versatility

Vmatch allows a multitude of different matching tasks to be solved using the persistent index. Every matching task is basically characterized by (1) the *kind of sequences* to be matched, (2) the *kind of matches* sought, (3) additional *constraints* on the matches, and (4) the *kind of post-processing* to be done with the matches.

In the standard case, *Vmatch* matches sequences over the same alphabet. Additionally, DNA sequences can be matched against a protein sequence index in all six reading frames. Finally, DNA sequences can be transformed in all six reading frames and compared against itself.

Where appropriate, *Vmatch* can compute the following kinds of matches, using state-of-the-art algorithms:

- maximal repeats using the algorithm of [2].
- branching tandem repeats using the algorithm of [1],
- supermaximal repeats using the algorithm of [2].
- maximal substring matches using the algorithm of [6].
- maximal unique substring matches using the algorithm of [6].
- complete matches using the algorithms of [8] and [9]

To compute degenerate substring matches or degenerate repeats, each kind of match (with the exception of tandem repeats and complete matches) can be taken as an exact seed and extended by either of two different strategies:

- the *maximum error* extension strategy, as described in [7] for repeat detection,
- the *greedy* extension strategy of [12].

Matches can be selected according to their length, their E-value, their identity value, or match score.

In the standard case, a match is displayed as an alignment including positional information. Alternatively, a match can directly be postprocessed in different ways:

- *inverse output*, i.e. reporting of substrings *not* covered by a match.
- *masking* of substrings covered by a match.
- *clustering* of sequences according to the matches found.
- *chaining* of matches, i.e. finding optimal subsets of matches which do not cross, using the algorithms described in [3].
- *clustering* of matches according to pairwise sequence similarities computed by the dynamic programming algorithm of [10].
- *clustering* of matches according to the positions where they occur, following the approach of [11].

Efficient algorithms and data structures

Vmatch is based on enhanced suffix arrays described in [2]. This data structure has been shown to be as powerful as suffix trees, with the advantage of a reduced space requirement and reduced processing time. Careful implementation of the algorithms and data structures incorporated in *Vmatch* have led to exceedingly fast and robust software, allowing very large sequence sets to be processed quickly. The 32-bit version of *Vmatch* can process up to 400 million symbols, if enough memory is available. For large server class machines (e.g. SUN-Sparc/Solaris, Intel Xeon/Linux, Compaq-Alpha/Tru64) *Vmatch* is available as a 64 bit version, enabling gigabytes of sequences to be processed.

Flexible input format

The most common formats for input sequences (Fasta, Genbank, EMBL, and SWISSPROT) are accepted. The user does not have to specify the input format. It is automatically recognized. All input files can contain an arbitrary number of sequences. Gzipped compressed inputs are accepted.

Customized output and match selection

Vmatch's output can be parsed by other programs easily. Furthermore, several options allow for its customization. XML output is available and new output formats can easily be incorporated without changing *Vmatch*'s program code. Certain matches can easily be selected by user defined criteria, without intermediate output and subsequent parsing.

The parts of Vmatch

Up until now we have referred to *Vmatch* as a collection of programs. In the following we use the same name, `vmatch` (in typewriter font), for the most important program in this collection. Besides `vmatch`, there are the following programs available:

1. `mkvtree` constructs the persistent index and stores it on files.
2. `mkdna6idx` constructs an index for a DNA sequence after translating this in all six reading frames.
3. `vseqinfo` delivers information about indexed database sequences.
4. `vstree2tex` outputs a representation of the index in LaTeX-format. It can be used, for example, for educational or debugging purposes.
5. `vseqselect` selects indexed sequences satisfying specific criteria.

6. `vsubseqselect` selects substrings of a specified length range from an index.

7. `vmigrate.sh` converts an index from big endian to little endian architectures, or vice versa.

8. `vmatchselect` sort and selects matches delivered by `vmatch`.

9. `chain2dim` computes optimal chains of matches from files in *Vmatch*-format.

10. `matchcluster` computes clusters of matches from files in *Vmatch*-format.

Figure 1 depicts the data flow in the different tools described in this manual.

Overview

The rest of this document is organized as follows: In Section 2 we describe `mkvtree`, a program which constructs the index. For a DNA sequence we can construct a six-frame index using the programming `mkdna6idx`, described in section 3. Section 4 is devoted to the program `vseqinfo` which delivers information about the indexed database sequences. In Section 5 we describe the program `vstree2tex` which outputs the tables of a virtual suffix tree in LaTeX-format. Section 6 is devoted to the program `vseqselect` which allows to select sequences from an index satisfying specific criteria. Similarly, the program `vsubseqselect` described in Section 7 selects substrings of a specified length range from an index. Section 8 describes the shell-script `vmigrate.sh` to convert an index from big endian (e.g. SUN/Sparc or SGI/IRIX) to little endian (e.g. Linux or Compaq-Alpha) architectures, or vice versa. In Section 9 we explain how to use the program `vmatch` which allows to solve a variety of matching tasks on the index. Section 10 is devoted to the program `vmatchselect`, which allows to sort and select matches delivered by `vmatch`. Each of these sections is completed with several examples showing how to apply the different programs to solve the different matching tasks. In the course of this, the output format is explained.

In Appendix A we define some basic notions. Appendix B explains the format of the symbol mapping. Appendix C goes into detail about the X-drop extension strategy. Substring Specifications are described in Appendix D. Appendix E is devoted to the concept of selection functions. Finally Appendix F explains the tables the index consists of.

2 `mkvtree`: Making Virtual Suffix Trees

The program `mkvtree` constructs an *index* for a given set of sequences These are given as a list of *input files*. The sequences are referred to as *database sequences*. They can be over any given *alphabet*. The alphabet can be the DNA alphabet, or the protein alphabet, or any other alphabet consisting of printable characters. An alphabet is specified by a file storing a *symbol mapping*. The index consists of several files, the *index files*. Each such file stores a different table. The

Figure 1: The Dataflow in *Vmatch*. The programs are shown in ellipses. The inputs are the database and the query sequences and the alphabet transformation, represented by rectangles. At the center of all computations the persistent index is shown. All other rectangles represent the different kinds of output.

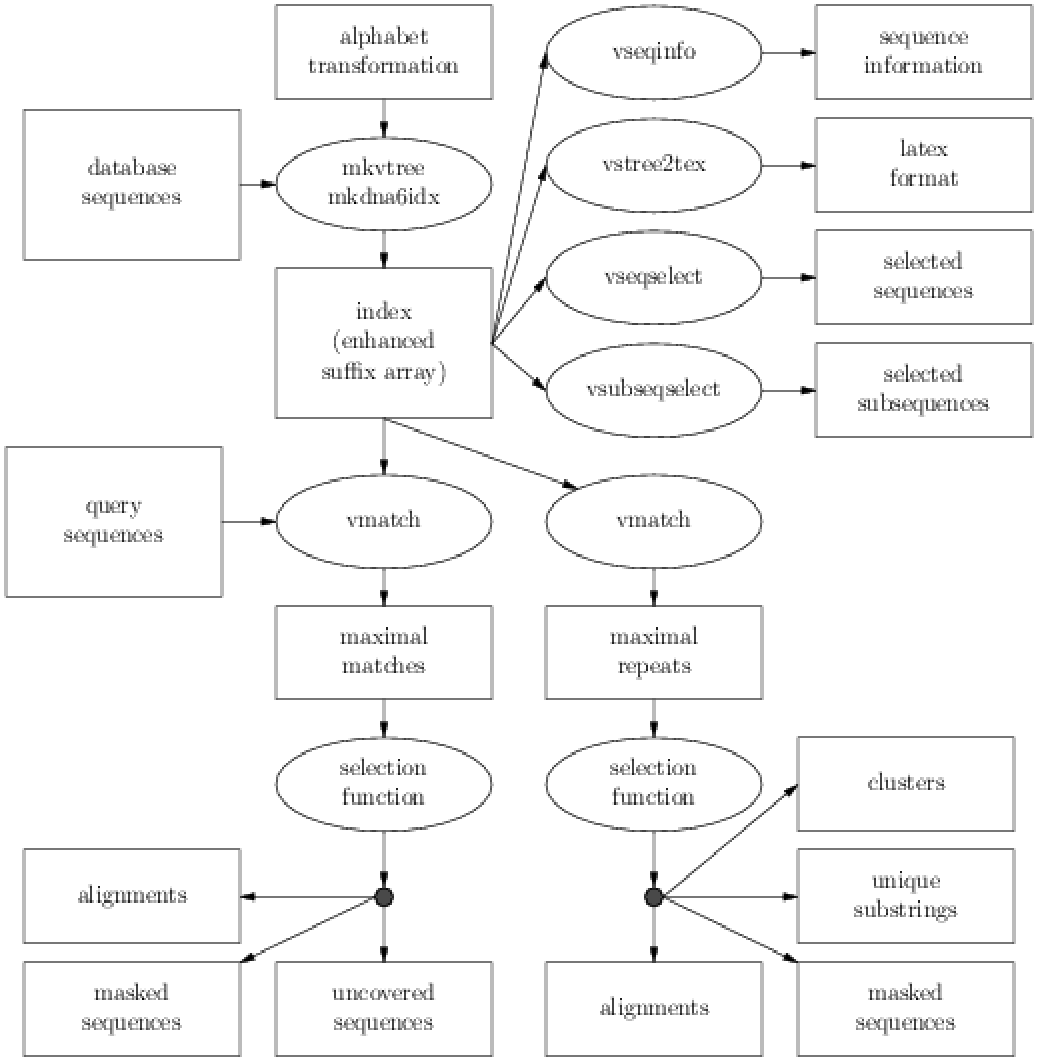

user specifies which tables (i.e. which part of the index) is written to a file, using one of eight output options, or a single option specifying that all tables are written to file. Appendix F gives a comprehensive description of the different tables. We recommend the user to only use the output options `-tis`, `-ois`, `-suf`, `-sti1`, `-bwt`, `-bck`, and `-lcp`. Option `-skp` is only necessary for approximate pattern matching.

If an error occurs, the program exits with error code 1. Otherwise, the exit code is 0. We support the following formats for the input files. They are recognized according to the first non-white space symbol in the file.

multiple FASTA format If the file begins with the symbol >, then this file is considered to be a file in multiple FASTA format (i.e. it contains one or more sequences). Each line starting with the symbol > contains the *description* of the sequence following it. Each line not starting with the symbol > contains the sequence. Empty lines are allowed and ignored when reading the input.

multiple EMBL/SWISSPROT format If the file begins with the string `ID`, then this file is considered to be a file in multiple EMBL format (i.e. containing one or more sequences, each in EMBL-format). The information contained in the `ID` and `DE`-lines is taken as the *description* of the corresponding sequence. The EMBL format is identical to the SWISSPROT format (w.r.t. the information we need to extract from such entries). So one can also use files in multiple SWISSPROT format as input.

multiple GENBANK format If the file begins with the string `LOCUS`, then this file is considered to be a file in multiple GENBANK format (i.e. containing one or more entries in GENBANK-format). The information contained in the `LOCUS` and the `DEFINITION`-lines is taken as the *description* of the corresponding sequence.

plain format If the file does not begin with the symbol > or the strings `ID` or `LOCUS`, then the file is taken verbatim. That is, the entire file is considered to be the input sequence (white spaces are *not* ignored).

There is no special option necessary to tell the program the sequence format. It automatically detects the appropriate format, according to the rules given above. If none of the above rules apply, then the program cannot recognize the input format and exits with error code 1. In such a case please check you input files for if they are conform with the input formats above. Another good solution is to use a more versatile sequence format transformation programs (e.g. `readseq`) to first generate multiple FASTA files and then feed this into `mkvtree`.

Today many files containing sequence files are provided compressed by the program `gzip`. To simplify the use of these files, `mkvtree` also accepts gzipped input files. These files must have the ending `.gz`. The gzipped formatted files are gunzipped internally and then processed as any other file.

Table 1: Overview of the `mkvtree`-Options

`-db`	specify database files (mandatory)
`-q`	specify query files
`-smap`	specify file containing a symbol mapping
`-dna`	input is DNA sequence
`-protein`	input is Protein sequence
`-indexname`	specify name for index to be generated
`-pl`	specify prefix length for bucket sort
`-rev`	use reverse input sequence
`-tis`	output transformed input sequences (tistab) to file
`-ois`	output original input sequences (oistab) to file
`-suf`	output suffix array (suftab) to file
`-sti1`	output reduced inverse suffix array (sti1tab) to file
`-bwt`	output Burrows-Wheeler Transformation (bwttab) to file
`-bck`	output bucket boundaries (bcktab) to file
`-lcp`	output longest common prefix lengths (lcptab) to file
`-skp`	output skip values (skptab) to file
`-allout`	output all index tables to files
`-v`	verbose mode
`-help`	this option

2.1 The Options of `mkvtree`

In this subsection, we describe the program `mkvtree` by explaining its options. An option is always beginning with the dash symbol. We write an option in `typewriter` mode. Arguments to options are written in typewriter mode if they are to be used verbatim. We use *italics* for an option argument if it is to be replaced by a some value. Table 1 gives an overview of the option available in `mkvtree`.

The program is called as follows:

`mkvtree` *options*

And here is a description of the options:

`-db` *dbfiles*
: Specify a non empty list of database files separated by white spaces. Each database file contains sequences in one of the formats as specified above. However, the format of all files has to be identical. The sequence must consist of characters over the alphabet as specified by the options `-dna`, `-protein`, or `-smap`, see below. White spaces are ignored. This option is mandatory.

`-smap` *mapfile*
: Specify the file storing the symbol mapping. If the given *mapfile* cannot be found in the directory where `mkvtree` is run, then all directories specified by the environment variable `MKVTREESMAPDIR` are searched. If defined correctly, this contains a list of directory paths

separated by colons (`':'`). For example, if one uses the `csh` or the `tcsh`, the definition of the environment-variable could look like this:

```
$ setenv MKVTREESMAPDIR "$HOME/vstree:/usr/vstree/TRANS"
```

For the `bash` or the `sh` the definition could look like

```
$ MKVTREESMAPDIR="$HOME/vstree:/usr/vstree/TRANS"
$ export MKVTREESMAPDIR
```

Then, if *mapfile* is not available in the current directory, `mkvtree` searches for *mapfile* in the two given directories. It scans the directory-list from left to right. As soon as it has found the file it stops. If the file cannot be found an error message is reported and the program exits with error code 1. See Appendix B for a more detailed explanation of the format of the symbol mapping file.

`-dna`

This option is equivalent to the option `-smap` *mapfile* where *mapfile* stores exactly the following 5 lines:

```
aA
cC
gG
tTuU
nsywrkvbdhmNSYWRKVBDHM
```

This specifies an alphabet of size 4 with additional wildcard symbols appearing in the fifth line. See Appendix B for a more detailed explanation of the format of the symbol mapping file.

`-protein`

This option is equivalent to the option `-smap` *mapfile* where *mapfile* stores exactly the following 21 lines:

```
L
V
I
F
K
R
E
D
A
G
S
T
N
```

```
Q
Y
W
P
H
M
C
XUBZJO*-
```

This specifies an alphabet of size 20 with additional wildcard symbols on the last line. See Appendix B for a more detailed explanation of the format of the symbol mapping file.

`-indexname` *filepath*
Specify *filepath* to be the name of the index, later referred to by *indexname*. This option is mandatory, if more than one database file is given and if additionally at least one file comprising the index is stored (i.e. if any of the output options is used). If no file from the index is stored, then this option is not allowed. If there is only one database file, and this option is not given, then *indexname* is the basename of the given *filepath*, i.e. the filename stripped by the directory path where it is stored. The *filepath* can be a complete path.

`-pl` [*prefixlength*]
Start the sorting of the suffixes with a initial step that sort all suffixes according to their prefixes of length *prefixlength*. The argument *prefixlength* is optional. Hence it is denoted in square brackets. If the argument is omitted, then the value for *prefixlength* is automatically determined. More precisely, it is $\lfloor \log_k \frac{n}{8} \rfloor$, where n is the total length of the database sequences and k is the alphabet size ($k = 4$ for DNA, $k = 20$ for proteins, and $k =$ one less than the number of lines in the corresponding symbol mapping file). Table 2 shows the choices for the *prefixlength* for DNA and protein sequences, given different ranges of values for n. The maximum value for *prefixlength* is $\lfloor \log_k \frac{4n}{8} \rfloor$. If you want to choose the prefix length manually, then we recommend *prefixlength* $\in [4,13]$ for DNA sequences, and *prefixlength* $\in [2,5]$ for protein sequences, depending on the total length of the input sequences, see Table 2. If this option is not specified, then the value for *prefixlength* is 0, in which case no initial bucket sort is performed. Note that in case you want to match a query against the index, the least length for the search must be at least *prefixlength*.

`-tis`
Store the table *tistab* in file *indexname*`.tis`.

`-ois`
Store the table *oistab* in file *indexname*`.ois`.

`-suf`
Store the table *suftab* in file *indexname*`.suf`.

`-sti1`
Store the reduced version of the inverse suffix table *stitab* in file *indexname*`.sti1`.

Table 2: The value of *prefixlength* automatically determined for different ranges for DNA and proteins and different input sizes.

DNA	
range of n	*prefixlength*
513 – 2,048	4
2,049 – 8,192	5
8,193 – 32,768	6
32,769 – 131,072	7
131,073 – 524,288	8
524,289 – 2,097,152	9
2,097,153 – 8,388,608	10
8,388,609 – 33,554,432	11
33,554,433 – 134,217,728	12
134,217,729 – 536,870,912	13

protein	
range of n	*prefixlength*
161 – 3,200	2
3,201 – 64,000	3
64,001 – 1,280,000	4
1,280,001 – 25,600,000	5

`-bwt`

Store the table *bwttab* and the integer i satisfying $\mathit{suftab}[i] = 0$ in file *indexname*`.bwt`.

`-bck`

Store the table *bcktab* in file *indexname*`.bck`.

`-lcp`

Store the tables *lcptab* and *llvtab* in the files *indexname*`.lcp` and *indexname*`.llv`.

`-skp`

Store the table *skptab* in file *indexname*`.skp`.

`-allout`

This option is equivalent to the combination of the output options `-tis`, `-ois`, `-suf`, `-sti1`, `-bwt`, `-bck`, `-lcp`, and `-skp`.

`-v`

Be verbose, that is, give reports about the different steps as well as the resource requirements of the computation. This option is recommended.

`-version`

Show the version of the Vmatch version, the program is part of. Also report the compilation date and the compilation options.

`-help`

Show a summary of all options and terminate with exit code 0.

Note the following when combining options of `mkvtree`:

- If any of the output options `-tis`, `-ois`, `-suf`, `-bwt`, `-lcp`, `-bck`, `-skp`, or `-allout` is used, then tables *prjtab*, *ssptab*, *destab*, and *sdstab* are stored in the files *indexname*.`prj`, *indexname*.`ssp`, *indexname*.`des`, and *indexname*.`sds`.
- Option `-allout` cannot be combined with any other output option.
- Option `-bck` requires that the option `-pl` is used.
- Option `-skp` requires that the options `-lcp` and `-suf` are used.
- The options `-smap`, `-dna`, and `-protein` pairwise exclude each other. If the input files are in FASTA-, EMBL-, SWISSPROT-, or GENBANK-format, then one of these three options is required. If the input files are not in FASTA-, EMBL-, SWISSPROT-, or GENBANK-format, then none of these option is allowed, and the symbol transformation is automatically generated satisfying the following: the symbol in the input files with the smallest ASCII code is mapped to 0, the symbol with the second smallest ASCII code is mapped to 1, etc.
- There must be at least one database file. The maximum number of database files is 256.

In case an error occurs, the error code of `mkvtree` is 1. Otherwise, it the exit code is 0.

2.2 Applying `mkvtree`

Suppose we have a file `atEST` in multiple FASTA format containing several EST sequences from the *Arabidopsis thaliana* genome.

```
>gi|5587835|gb|AF078689.1|AF078689 AF078689 Arabidopsis thaliana
AGTGGCTACGGCGGCGGTGGCGGCGATGGAAGTAGACGGATGGTGGTAGGAATGAAAGGCTAGAAGCGGC
GGAGAAGTATGTGGATAAGAAATAACAAAAACTGAGGGGATCATGAAGTTCTTCGTTATATTATAGTTTT
CAATCTGAATTTCAATTCCGCCGCTCGCCTTTTTCCTCTCCGCCTTTTCCGTCTCTCCGATCTGCTCCCG
CCGCCGACCTTGTGATGATTATAGCTCTGAAGGTCCATACAAGGATATAAAAAAAAAAAAAAAAA
>gi|4714049|dbj|C99932.1|C99932 C99932 Arabidopsis thaliana
ATGRAYAMTCAAAAATGGAGTMTAGGTTTCATATCTCTCGCTTTTCTCTTCATCACTTCCTCTTCAGCTG
...
```

The size of the file is about one megabyte:

```
$ ls -l atEST
-rw-r-----   1 kurtz    users   999815 Nov  6 21:37 atEST
```

We want to construct an index using `atEST` as a database file. Since the sequences in `atEST` are DNA sequences, we use the option `-dna` when constructing the index. We add the option `-pl`, since we want to construct table *bcktab*. Moreover, we specify option `-allout` to create all files comprising the index, and `-v` to obtain information about what the program is doing, and the resources it requires.

```
$ mkvtree -db atEST -dna -pl -allout -v
reading file "atEST"
maximal value for argument of option -pl is 9, recommended value is 8
total length of sequences: 772376 (including 1951 separators)
alphabet of size 5: acgtn
creating file "atEST.tis"
creating file "atEST.ois"
creating file "atEST.des"
creating file "atEST.sds"
creating file "atEST.lcp"
initializing data structures
sorting suffixes according to prefix of length 8
sorting all buckets
creating file "atEST.llv"
creating file "atEST.bck"
creating file "atEST.suf"
creating file "atEST.sti1"
creating file "atEST.bwt"
creating file "atEST.prj"
creating file "atEST.al1"
creating file "atEST.skp"
overall space peak: main=5.34 MB (7.25 bytes/symbol), secondary=1.69 MB
```

The previous call of `mkvtree` has constructed several index files:

```
$ ls -l atEST.*
-rw-r-----  1 kurtz gistaff      37 2005-02-01 01:43 atEST.al1
-rw-r-----  1 kurtz gistaff  524288 2005-02-01 01:43 atEST.bck
-rw-r-----  1 kurtz gistaff  772381 2005-02-01 01:43 atEST.bwt
-rw-r-----  1 kurtz gistaff  215445 2005-02-01 01:43 atEST.des
-rw-r-----  1 kurtz gistaff  772377 2005-02-01 01:43 atEST.lcp
-rw-r-----  1 kurtz gistaff   18536 2005-02-01 01:43 atEST.llv
-rw-r-----  1 kurtz gistaff  772376 2005-02-01 01:43 atEST.ois
-rw-r-----  1 kurtz gistaff     179 2005-02-01 01:43 atEST.prj
-rw-r-----  1 kurtz gistaff    7812 2005-02-01 01:43 atEST.sds
-rw-r-----  1 kurtz gistaff 3089508 2005-02-01 01:43 atEST.skp
-rw-r-----  1 kurtz gistaff    7804 2005-02-01 01:43 atEST.ssp
-rw-r-----  1 kurtz gistaff  772377 2005-02-01 01:43 atEST.sti1
-rw-r-----  1 kurtz gistaff 3089508 2005-02-01 01:43 atEST.suf
-rw-r-----  1 kurtz gistaff  772376 2005-02-01 01:43 atEST.tis
```

Currently, none of the programs described here requires the table with the skip-values. Hence the file `atEST.skp` can be safely removed. You can of course directly specify which files you want to generate by giving the appropriate output options.

Here is an example constructing an index for a complete genome, the pathogen *Ecoli O157:H7*. This is stored in the file `EcoliO157H7`. Now we explicitely ask for the construction of a subset of the tables comprising the index, using the options `-sti1`, `-bwt`, `-bck`, `-suf`, `-lcp`, `-tis`, and `-ois`.

```
$ mkvtree -db EcoliO157H7 -v -pl -sti1 -bwt -dna -bck -suf -lcp -tis -ois -skp
reading file "EcoliO157H7"
maximal value for argument of option -pl is 10, recommended value is 9
total length of sequences: 5471376
alphabet "aAcCgGtTuUnsywrkvbdhmNSYWRKVBDHM" (size 32) mapped to "acgtn" (size 5)
create file "EcoliO157H7.tis"
create file "EcoliO157H7.ois"
create file "EcoliO157H7.des"
create file "EcoliO157H7.sds"
create file "EcoliO157H7.lcp"
initializing data structures
sorting suffixes according to prefix of length 9
sorting all buckets
create file "EcoliO157H7.llv"
create file "EcoliO157H7.bck"
create file "EcoliO157H7.suf"
create file "EcoliO157H7.sti1"
create file "EcoliO157H7.bwt"
create file "EcoliO157H7.prj"
create file "EcoliO157H7.al1"
create file "EcoliO157H7.skp"
overall space peak: main=32.46 MB (6.22 bytes/symbol), secondary=10.50 MB
```

The previous call of `mkvtree` has constructed several index files:

```
$ ls -l EcoliO157H7.*
-rw-r-----  1 kurtz gistaff       37 2005-02-01 23:18 EcoliO157H7.al1
-rw-r-----  1 kurtz gistaff  2097152 2005-02-01 23:18 EcoliO157H7.bck
-rw-r-----  1 kurtz gistaff  5471381 2005-02-01 23:18 EcoliO157H7.bwt
-rw-r-----  1 kurtz gistaff       30 2005-02-01 23:18 EcoliO157H7.des
-rw-r-----  1 kurtz gistaff  5471377 2005-02-01 23:18 EcoliO157H7.lcp
-rw-r-----  1 kurtz gistaff  1031440 2005-02-01 23:18 EcoliO157H7.llv
-rw-r-----  1 kurtz gistaff  5471376 2005-02-01 23:18 EcoliO157H7.ois
-rw-r-----  1 kurtz gistaff      186 2005-02-01 23:18 EcoliO157H7.prj
-rw-r-----  1 kurtz gistaff        8 2005-02-01 23:18 EcoliO157H7.sds
-rw-r-----  1 kurtz gistaff  5471377 2005-02-01 23:18 EcoliO157H7.sti1
-rw-r-----  1 kurtz gistaff 21885508 2005-02-01 23:18 EcoliO157H7.suf
-rw-r-----  1 kurtz gistaff  5471376 2005-02-01 23:18 EcoliO157H7.tis
```

Of course, we can also construct an index from more than one file. Suppose we have two bacterial genomes in the files `mgen.fna` and `mpneu.fna`: The genome of *M. genitalis* and the genome of *M. pneunomiae*.

```
$ ls -l mgen.fna mpneu.fna
-rw-r-----   1 kurtz    users      589593 Oct 23 18:53 mgen.fna
-rw-r-----   1 kurtz    users      829789 Oct 23 18:53 mpneu.fna
```

```
$ mkvtree -dna -pl -bck -tis -lcp -suf -indexname bac -db mgen.fna mpneu.fna
```

Since there are two input files, we have to give a name `bac` for the index, using the option `-indexname`. Since we have not used the verbose option `-v`, the program works silently.

Now let us consider an example where we construct an index from a set of protein sequences given in SWISSPROT-format. The corresponding input file `swissp.sp.gz` is in `gzip` format. After gunzipping the file, the contents looks like this:

```
$ zcat swissp.sp.gz
ID   110K_PLAKN     STANDARD;      PRT;   296 AA.
AC   P13813;
DT   01-JAN-1990 (Rel. 13, Created)
DT   01-JAN-1990 (Rel. 13, Last sequence update)
DT   01-FEB-1994 (Rel. 28, Last annotation update)
DE   110 KD ANTIGEN (PK110) (FRAGMENT).
OS   Plasmodium knowlesi.
OC   Eukaryota; Alveolata; Apicomplexa; Haemosporida; Plasmodium.
RN   [1]
RP   SEQUENCE FROM N.A.
RX   MEDLINE; 88039002.
RA   PERLER F.B., MOON A.M., QIANG B.Q., MEDA M., DALTON M., CARD C.,
RA   SCHMIDT-ULLRICH R., WALLACH D., LYNCH J., DONELSON J.E.;
RT   "Cloning and characterization of an abundant Plasmodium knowlesi
RT   antigen which cross reacts with Gambian sera.";
RL   Mol. Biochem. Parasitol. 25:185-193(1987).
DR   EMBL; M19152; AAA29471.1; -.
DR   PIR; A54527; A54527.
KW   Malaria; Antigen; Repeat.
FT   NON_TER       1      1
FT   DOMAIN      131    296       13.5 X 12 AA TANDEM REPEATS OF E-E-T-Q-K-
FT                                T-V-E-P-E-Q-T.
SQ   SEQUENCE   296 AA;  34077 MW;  666F88DF CRC32;
     FNSNMLRGSV CEEDVSLMTS IDNMIEEIDF YEKEIYKGSH SGGVIKGMDY DLEDDENDED
     EMTEQMVEEV ADHITQDMID EVAHHVLDNI THDMAHMEEI VHGLSGDVTQ IKEIVQKVNV
     AVEKVKHIVE TEETQKTVEP EQIEETQNTV EPEQTEETQK TVEPEQTEET QNTVEPEQIE
     ETQKTVEPEQ TEEAQKTVEP EQTEETQKTV EPEQTEETQK TVEPEQTEET QKTVEPEQTE
     ETQKTVEPEQ TEETQKTVEP EQTEETQKTV EPEQTEETQN TVEPEPTQET QNTVEP
//
```

We use the gzipped-file like any other file. `mkvtree` automatically recognizes that it is in gzipped format (due to the suffix `.gz`). It gunzips the file internally, and extracts the required sequence information. Of course, any other sequence formats can also be in gzipped format.

```
$ mkvtree -protein -db swissp.sp.gz -pl -lcp -suf -tis -ois -bwt -bck -v
reading gzipped file "swissp.sp.gz"
maximal value for argument of option -pl is 2, recommended value is 2
total length of sequences: 4566 (including 7 separators)
alphabet of size 21: LVIFKREDAGSTNQYWPHMCX
creating file "swissp.sp.gz.tis"
creating file "swissp.sp.gz.ois"
creating file "swissp.sp.gz.des"
creating file "swissp.sp.gz.sds"
creating file "swissp.sp.gz.lcp"
initializing data structures
sorting suffixes according to prefix of length 2
sorting all buckets
creating file "swissp.sp.gz.llv"
creating file "swissp.sp.gz.bck"
```

```
creating file "swissp.sp.gz.suf"
creating file "swissp.sp.gz.bwt"
creating file "swissp.sp.gz.prj"
creating file "swissp.sp.gz.al1"
overall space peak: main=0.18 MB (40.80 bytes/symbol), secondary=0.00 MB
```

The description of the sequences is extracted from the `ID`-field and the `DE`-field of the SWISSPROT-entry. For the first sequence, stored in the input file `swissp.sp.gz`, `mkvtree` extracts the following description:

```
110K_PLAKN 110 KD ANTIGEN (PK110) (FRAGMENT).
```

We now consider a Fasta-File with two protein sequences:

```
$ cat Proteinseq
>
QPTFFLLPPGEGGAESYFNNIVKRRQTNMFILNNYYFHSKRIRTIEELAEMYLDQVRG
>
QGPTLFLLPPGEGGSYFNNIVKRLRQTNMVVFNNYYLHSKRLRTFEELAEMYLDQVRG
```

We produce an index from this file, applying the symbol mapping `TransProt11`:

```
$ mkvtree -db Proteinseq -smap TransProt11 -pl -allout -v
reading file "Proteinseq"
maximal value for argument of option -pl is 1, recommended value is 1
total length of sequences: 117 (including 1 separator)
alphabet "LVIFKREDAGSTNQYWPHMCXUBZ*-" (size 26) mapped to "i+-sonaphmcx" (size 12)
create file "Proteinseq.tis"
create file "Proteinseq.ois"
create file "Proteinseq.des"
create file "Proteinseq.sds"
create file "Proteinseq.lcp"
initializing data structures
sorting suffixes according to prefix of length 1
sorting all buckets
create file "Proteinseq.llv"
create file "Proteinseq.bck"
create file "Proteinseq.suf"
create file "Proteinseq.sti1"
create file "Proteinseq.bwt"
create file "Proteinseq.prj"
create file "Proteinseq.al1"
create file "Proteinseq.skp"
overall space peak: main=0.13 MB (1171.36 bytes/symbol), secondary=0.00 MB
```

Note that the fourth line of the output reports that the input alphabet is the usual amino acid alphabet including some wildcards, while the transformed alphabet is of size 12.

3 `mkdna6idx`: generating a six frame translation index

`mkdna6idx` is very similar to `mkvtree`. While `mkvtree` can handle sequences over arbitrary alphabets, `mkdna6idx` requires DNA-sequences as input. It generates two indices, namely:

- A flat index *indexname* for the the given DNA sequences. It mainly consists of the two files *indexname*`.tis` and *indexname*`.ois`. This index is mainly used for output purpose.
- An index *indexname.6fr* for the given DNA sequences translated in all six reading frames. This is used for computing the matches.

These two indices allow `vmatch` to compute matches on the protein level.

The program is called as follows:

`mkdna6idx` *options*

And here is a description of the options:

`-db` *dbfiles*
: Specify a non empty list of database files separated by white spaces. Each database file contains sequences in one of the following formats: Fasta, Genbank, EMBL, and SWISSPROT. The user does not have to specify the input format. However, the format of all files has to be identical. The sequence must consist of characters over the alphabet `a`, `c`, `g`, `t`, or `u` (in lower or upper case), or wildcards `n`, `s`, `y`, `w`, `r`, `k`, `v`, `b`, `d`, `h`, `m`. White spaces in the input files are ignored. This option is mandatory. This option is identical with the same option of the program `mkvtree`.

`-smap` *mapfile*
: Specify the file storing the symbol mapping applied to the amino acid symbols after the six frame translation. If the given *mapfile* cannot be found in the directory where `mkvtree` is run, then all directories specified by the environment variable `MKVTREESMAPDIR` are searched. If defined correctly, this contains a list of directory paths separated by colons (`':'`). For example, if one uses the `csh` or the `tcsh`, the definition of the environment-variable could look like this:

```
setenv MKVTREESMAPDIR "$HOME/vstree:/usr/vstree/TRANS"
```

For the `bash` or the `sh` the definition could look like

```
MKVTREESMAPDIR="$HOME/vstree:/usr/vstree/TRANS"
export MKVTREESMAPDIR
```

Then, if *mapfile* is not available in the current directory, `mkvtree` searches for *mapfile* in the two given directories. It scans the directory-list from left to right. As soon as it has found the file it stops. If the file cannot be found, an error message is reported and the program exits with error code 1. See Appendix B for a more detailed explanation of the format of the symbol mapping file.

`-transnum` *t*

Specify the number of the codon translation table used for the six frame translation. t must be a number in the range $[1,23]$ except for 7, 8, 17, 18, 19 and 20. Table 3 gives the possible numbers and their names. The codon translation tables, their numbers, and their names were taken from `ftp://ftp.ncbi.nih.gov/entrez/misc/data/gc.prt`. If this option is not used, then the standard codon translation table (number 1) is used.

`-indexname` *filepath*

Specify *filepath* to be the name of the index, later referred to by *indexname*. This option is mandatory, if more than one database file is given and if additionally at least one file comprising the index is stored (i.e. if any of the output options is used). If no file from the index is stored, then this option is not allowed. If there is only one database file, and this option is not given, then *indexname* is the basename of the given *filepath*, i.e. the filename stripped by the directory path where it is stored. The *filepath* can be a complete path.

`-tis`

Store the table *tistab* in file *indexname.6fr*`.tis`.

`-ois`

Store the table *oistab* in file *indexname.6fr*`.ois`.

`-v`

Be verbose, that is, give reports about the different steps as well as the resource requirements of the computation. This option is recommended.

`-version`

Show the version of the program. Also report the compilation date and the compilation options.

`-help`

Show a summary of all options and terminate.

An example application of `mkdna6idx` is given in section 9.9.4.

4 `vseqinfo`: Obtaining Sequence Information

`vseqinfo` echoes for each database sequence its length and its description. The program has no options. It takes exactly one argument, namely the index name. The output goes to standard out.

4.1 Applying `vseqinfo`

We can obtain the information about the database sequences in `atEST`.

1	Standard
2	Vertebrate Mitochondrial
3	Yeast Mitochondrial
4	Mold Mitochondrial; Protozoan Mitochondrial; Coelenterate Mitochondrial; Mycoplasma; Spiroplasma
5	Invertebrate Mitochondrial
6	Ciliate Nuclear; Dasycladacean Nuclear; Hexamita Nuclear
9	Echinoderm Mitochondrial
10	Euplotid Nuclear
11	Bacterial
12	Alternative Yeast Nuclear
13	Ascidian Mitochondrial
14	Flatworm Mitochondrial
15	Blepharisma Macronuclear
16	Chlorophycean Mitochondrial
21	Trematode Mitochondrial
22	Scenedesmus Obliquus Mitochondrial
23	Thraustochytrium Mitochondrial

Table 3: The possible codon translation table numbers and table names.

```
$ vseqinfo atEST
0 275 gi|5587835|gb|AF078689.1|AF078689 AF078689 Arabidopsis thaliana lib
1 852 gi|4714049|dbj|C99932.1|C99932 C99932 Arabidopsis thaliana library
2 719 gi|4714048|dbj|C99931.1|C99931 C99931 Arabidopsis thaliana library
3 746 gi|4714047|dbj|C99930.1|C99930 C99930 Arabidopsis thaliana library
4 668 gi|4714046|dbj|C99929.1|C99929 C99929 Arabidopsis thaliana library
5 800 gi|4714045|dbj|C99928.1|C99928 C99928 Arabidopsis thaliana library
```

5 `vstree2tex`: Pretty Printing a Virtual Tree

The program `vstree2tex` produces a representation of a virtual suffix tree in LaTeX-format and outputs it to standard out.

Note that `vstree2tex` should only be used for very small indexes since it produces large output files. Suppose the total length of all sequences in the index is n. If the option `-s` is not used, then the output size of `vstree2tex` is about $10n$ bytes per option. (plus some constant number of bytes for the header and the footer of the LaTeX-file). If the option `-s` is used, then the size of the output is proportional to n^2. The program is mainly designed for debugging a program based on the index and for educational purposes.

The program is called as follows:

`vstree2tex` [*options*] *indexname*

And here is a description of the options:

`-s`
: Output the suffixes. The *i*th separator symbol in a multiple sequence is shown as *i*.

`-tis`
: Output table *tistab*. The *i*th separator symbol in a multiple sequence is shown as *i*.

`-ois`
: Output table *oistab*. The *i*th separator symbol in a multiple sequence is shown as *i*.

`-suf`
: Output table *suftab*.

`-sti1`
: Output table *stitab1*.

`-bwt`
: Output table *bwttab*. The *i*th separator symbol in a multiple sequence is shown as *i*.

`-bck`
: Output table *bcktab*.

`-lcp`
: Output table *lcptab*.

`-skp`
: Output table *skptab*.

`-help`
: Show a summary of all options and terminate with exit code 0.

5.1 Applying `vstree2tex`

Suppose a file with the following contents:

```
>a short sequence
acaaacatat
```

Let us construct the corresponding index:

```
$ mkvtree -dna -db smallseq.fna -pl -allout
```

Now run `vstree2tex` to produce the table in LaTeX-format:

Table 4: The output of `vstree2tex` after applying LaTeX to it.

i	*oistab*	*suftab*	*lcptab*	*bwttab*	$S_{suftab[i]}$
0	*a*	2		`c`	`aaacatat$`
1	*c*	3	2	`a`	`aacatat$`
2	*a*	0	1		`acaaacatat$`
3	*a*	4	3	`a`	`acatat$`
4	*a*	6	1	`c`	`atat$`
5	*c*	8	2	`t`	`at$`
6	*a*	1	0	`a`	`caaacatat$`
7	*t*	5	2	`a`	`catat$`
8	*a*	7	0	`a`	`tat$`
9	*t*	9	1	`a`	`t$`
10		10	0	`t`	`$`

w	$bcktab[\varphi(w)]$
`a`	(0,5)
`c`	(6,7)
`g`	(1,0)
`t`	(8,9)

```
$ vstree2tex -ois -suf -bwt -bck -lcp -s smallseq.fna > tmp.tex
```

Then the command

```
$ latex tmp.tex
```

produces Table 4.

6 `vseqselect`: Selecting Sequences

The program `vseqselect` selects sequences from a given index and prints them on standard out.

The program is called as follows:

`vseqselect` [*options*] *indexname*

And here is a description of the options:

`-minlength` α
: Select a sequence only if it is not shorter than α.

`-maxlength` ω
: Select a sequence only if it is not longer than ω.

`-seqnum` *filename*
: Select the sequences with the numbers given line by line in file *filename*.

`-randomnum` *k*
: Randomly select *k* sequences from the index.

`-randomlength` ℓ
: Randomly select sequences from the index until the total length of the selected sequences is ℓ or larger.

`-help`
: Show a summary of all options and terminate with exit code 0.

The options `-randomnum`, `-randomlength`, and `-seqnum` cannot be pairwise combined. However, each of these options can be combined with the option `-minlength`, and `-maxlength`. This allows the selection of sequences by random or by numbers specified in a file, satisfying additional constraints.

6.1 Applying `vseqselect`

Let us start with an example which randomly selects 3 sequences of length between 100 and 200 from the index `atEST`.

```
$ vseqselect -randomnum 3 -minlength 100 -maxlength 200 atEST
>gi|2764371|gb|T46124.1|T46124 9387 Lambda-PRL2 Arabidopsis thaliana cDNA clone 135
CCAAGCTCACATCCGATGCAGCCTTGGACCCCGCTGGTCTTGTNGCTGTCGCTGTGGCTC
ACGTCTTTNCCCTTTNNGTTGGTGTTTCCATAGCCGNCAACATCTCCGGCGGACACCTTA
ACCCCGCCGTNACACTTCGGTCTCGCCGTCGGTGGCAACATCACAG
>gi|3868949|dbj|AB015100.1|AB015100 AB015100 RIKEN-PMB-FL1 Arabidopsis thaliana cDN
TTTTTTGATGATGACAAGGTCTTAGCTTTGGTGGACATAACTCCCCAGGGTCCTGTTCAC
ATCCTCCTTATTCAAAAAGTGAGGGATGGCCTAACTGGCCTCTCTAAGGCTGAGGAAAGG
CACATCGACATCTTGGGCCACCTACTCTACACTGCCTAACTTTTCTCAAAACAAAAAGGC
CTAGCA
>gi|2764374|gb|T46197.1|T46197 9460 Lambda-PRL2 Arabidopsis thaliana cDNA clone 138
AATTTTTTTTNATTTCAGCAAAAAAGCAAAGGACTATACAGGTGATGGACATTCATGACA
TNCCTAAACCTAAGAAAGGTGGATTTTNCCCAAGAAAAGGCGGGAGTNCTCAAGGTGGTA
GACACNGGTAAATTGAGAAAGATGAGAGTTAAGTNAGGAAGAGAAAAAAAGACAGAGANC
AAAGCTCTGTTTCT
```

For each selected sequence which appears in the output, lines of the following form are output on standard error:

```
#     0: select sequence with number 1579 (length 166)
#     1: select sequence with number 161 (length 186)
#     2: select sequence with number 1576 (length 194)
```

The first number in each line is always the consecutive number of the selected sequences.

Alternatively we can randomly select sequences of some total length:

```
$ vseqselect -randomlength 300 -minlength 30 -maxlength 99 atEST
>gi|4713981|dbj|C99855.1|C99855 C99855 Arabidopsis thaliana library (Motohashi R) A
```

```
CCCGCCCTTTNAANCGAGTACAANACNGGGGGAAANGTTCCGAATAAAAAGTGGGG
>gi|4713999|dbj|C99875.1|C99875 C99875 Arabidopsis thaliana library (Motohashi R) A
TGGGGCNACCANCAANCTAANGAAGATCGAATTGGACACACTCNCGTCTATCACGCCAAC
ANCNCTGCATCTCTGTCTGCCCNATACGACTGCACGTG
>gi|4210234|gb|AI239394.1|AI239394 EST093 Arabidopsis pSMASH Library Arabidopsis th
ATACAACAATGGCTTCGTCATCTACAAGTATCTCTCTCCTTCTCTTCGTCTCTTTCCTTC
TTCTTCTCGTTAACTCGCGTGCAGAGAATGCGTGG
>gi|4210216|gb|AI239376.1|AI239376 EST078 Arabidopsis pSMASH Library Arabidopsis th
TAAGGCAATGCAAGCTTTGATCTTTCTTGGTTTCTTGGGCACTTCATGTCTCGCTCAAGC
TCCTGCACCAGCACCAACC
```

Now suppose we have stored the following sequence numbers in a file called `myseqnum`:

```
69
51
109
127
```

Then the program call

```
\$ vseqselect -seqnum myseqnum atEST
```

produces the same result as the previous call. A final example shows how to search for all sequences whose description matches the pattern `T46124.1`:

```
$ vseqselect -matchdesc T46124.1 atEST
>gi|2764371|gb|T46124.1|T46124 9387 Lambda-PRL2 Arabidopsis thaliana cDNA clone 135
CCAAGCTCACATCCGATGCAGCCTTGGACCCCGCTGGTCTTGTNGCTGTCGCTGTGGCTC
ACGTCTTTNCCCTTTNNGTTGGTGTTTCCATAGCCGNCAACATCTCCGGCGGACACCTTA
ACCCCGCCGTNACACTTCGGTCTCGCCGTCGGTGGCAACATCACAG
```

7 `vsubseqselect`: Selecting Subsequences

The program `vsubseqselect` selects subsequences from a given index and prints them on standard out, either line by line or in FASTA format. The selection can either be random or according to position ranges specified by the user.

The program is called as follows:

`vsubseqselect` [*options*] *indexname*

And here is a description of the options:

`-minlength` α
: Select subsequences from the index whose minimum length is α.

`-maxlength` ω
: Select subsequences from the index whose maximum length is ω.

`-snum` k

Select k sequences from the index in the specified length range (as given by the options `-minlength` and `-maxlength`).

`-range` k l

Report the substring in the index starting at position k and ending at position l.

`-seq` l n r

Report the substring in the index of length l in sequence number n starting at the relative position r.

`-help`

Show a summary of all options and terminate with exit code 0.

The three options `-minlength`, `-maxlength`, and `-snum` can only be used in combination with each other. No other option can be combined with any of these options. The combination of these options delivers sequences $s_0, s_1, \ldots, s_{k-1}$, such that for all $i \in [0, k-1]$ the following holds: If i is even, then s_i occurs in the index. If i is odd, then the reverse of s_i occurs in the index. Options `-range` and `-seq` exclude each other. Hence there are basically three choices of options:

- If the options `-minlength`, `-maxlength`, and `-snum` with argument k is used, then k subsequences are randomly selected, all of which occur in the given index. The lengths of the selected sequences are evenly distributed over the interval $[\alpha, \omega]$. Besides the sequences, the distribution of the length ranges of the selected subsequences is reported.
- If the option `-range` is used with two arguments k and l, then the substring starting at position k and ending at position l is shown.
- If the option `-seq` is used with three arguments l, n, and r, then the substring of length n in sequence number l starting at the relative position r is shown.

7.1 Applying `vsubseqselect`

The following command shows how to randomly select 5 subsequences of length between 30 and 40 from the index `atEST`:

```
$ vsubseqselect -minlength 30 -maxlength 40 -snum 5 atEST
agtttacttttctctcttcttgttttctgtgttggaag
tccttactgacttagtcatactcttctcaatc
cccattttatttcctctcaaatgctttccgtagt
atagtaagtaaaattgaactactgttattgagtt
gtttcttgtgtttgtgcttacgatactttcctc
# 1 subsequences of length 32
# 1 subsequences of length 33
# 2 subsequences of length 34
# 1 subsequences of length 38
```

Now suppose we want to select the first 30 residues of index `swissp.sp.gz`:

```
$ vsubseqselect -range 0 29 swissp.sp.gz
>110K_PLAKN 110 KD ANTIGEN (PK110) (FRAGMENT). swissp.sp.gz [0,29]
FNSNMLRGSVCEEDVSLMTSIDNMIEEIDF
```

8 `vmigrate.sh`: Migrating an Index

Some of the files comprising the virtual suffix tree contain integers in binary format. Such a binary format is not portable to a different computer architecture in general, since the byte order for 2-byte or 4-byte integers may differ. However, in some cases the user wants to construct a virtual suffix tree on a machine with large memory (e.g. a SUN-Sparc Server) and then use the virtual suffix tree on a machine with much less memory (e.g. a Linux-Laptop). For such a case the user should copy all files to the Laptop and then call `vmigrate.sh` *indexname*. This transforms the index with name *indexname*. That is, it reverses the byte order for all files containing 4-byte integers. A second call `vmigrate.sh` *indexname* results in the original files, i.e. it reverses the first transformation. The shell-script `vmigrate.sh` calls a program vendian which performs the transformation for the appropriate files. It is part of the virtual suffix tree distribution. `vmigrate.sh` takes the index as an argument and does not have any options.

8.1 Applying `vmigrate.sh`

Suppose we have created a very large index `vlsi` using `mkvtree` on a large, say SUN-server. This index cannot directly be used on our Linux-machine. So we convert it as follows:

```
$ vmigrate.sh vlsi
# migrate the indexfile vlsi.bck llv suf sds ssp skp
Converting vlsi.bck
Converting vlsi.llv
Converting vlsi.suf
Converting vlsi.sds
Converting vlsi.ssp
Converting vlsi.skp
Done.
```

The conversion of the byte order is only done for the files containing integers. The size of each converted file remains unchanged.

9 `vmatch`: Solving Matching Tasks

The program `vmatch` allows to solve a multitude of different matching tasks over an index constructed by `mkvtree`. Each matching task is solved by a combination of options specifying

- the *input*,
- the *kind of matches* sought,
- additional *constraints* on the matches,
- the *direction* of the matches (in case of DNA),
- the *kind of postprocessing* to be done,
- the *output mode* and *output format*.

Additionally, if there is more than one algorithm to solve a certain matching task, `vmatch` allows to specify which algorithm is to be used.

`vmatch` allows to compute the following kinds of matches:

(1) match all substrings of the database sequences against itself. The matches can be one of the following kinds:

  (a) *branching tandem repeats*, i.e. repeats where the two instances of the repeat occur at consecutive positions

  (b) *maximal repeats*, i.e. pairs of maximal substrings occurring more than once in the database sequences

  (c) *supermaximal repeats*, i.e. pairs of maximal substrings occurring more than once in the database sequences, but not in any other maximal repeat

(2) match a set of *query sequences* (given in an extra query file) against the index. The matches can be one of the following kinds:

  (a) *maximal substring matches*, i.e. the substrings of the query sequences matching substrings of the database sequences. All matches exceeding some minimum length, extended maximally to the left and to the right, are reported.

  (b) *maximal unique matches*, i.e. the substrings of the query sequences matching substrings of the database sequences. A match is reported if it is unique in the database sequences as well as in the query sequences.

  (c) *complete matches*, i.e. a query sequence must completely match (i.e. from the first character to the last character) a substring of the database sequences.

For all these match kinds, the matches themselves can be direct or palindromic (i.e. on the reverse strand, in case of DNA sequences). If required, DNA sequences are translated into six reading frames and the matches are computed on the protein level, and reported on the DNA level. Besides exact matches, also degenerate matches with a maximal number of errors (insertions, deletions, and mismatches) are supported. Moreover, degenerate matches can be derived from

exact matches by extending these using a greedy extension strategy. This does not apply to complete matches.

For all different match kinds, the matches delivered by `vmatch` can be selected according to their E-value, their identity value, or their match score.

In the default case, a match is reported as a formatted row of numbers, containing its lengths, the positions where it occurs, the E-value, the number of errors it contains, the match score, and the identity value. Optionally, an alignment of the sequences that are involved in the match can be reported.

An important feature of `vmatch` is the capability of directly postprocessing the matches found in the following ways:

(*i*) *inverse output*, i.e. report substrings of the database sequences or the query sequences *not* covered by a match

(*ii*) *masking* substrings of the database sequences or the query sequences covered by a match

(*iii*) *clustering* of a set of database sequences according to the matches found between these sequences. The output of this option can be a representation of the clusters, or a set of sequences each being representative for a cluster.

(*iv*) *chaining* of a set of matches, i.e. finding optimal subsets of all matches which do not cross

(*v*) *clustering* of matches according to the pairwise similarities on the sequences involved in the match.

(*vi*) *clustering* of matches according to the positions where they occur.

Finally, to accommodate many more kinds of user defined post processing tasks, `vmatch` provides the concept of selection functions. These provide an open interface which allow arbitrary on-the-fly postprocessing of the matches without output and parsing of the matches. For more details on this concept, see Appendix E.

Since the different kinds of matches can be postprocessed in different ways, using different direction of matches, or additional match constraints, or output modes, or output formats, the number of possible combinations of options is very large. Ignoring trivial variations of options (to e.g. modify the output format), we have generated 1386 different combinations of options. In Section 9.9 we can only give 43 examples for a few number of combinations of options.

`vmatch` is called as follows.

`vmatch` *options indexname*

The last argument of `vmatch` is always the *indexname*. This can be a complete path. The output of `vmatch` goes to standard output. `vmatch` currently has 39 options, described on about 9 pages of this manual. To allow a new user, to get acquainted with the program, we have divided

the options into different categories. An overview of the option categories with a short one-line description of each option is given in table 5.

9.1 Input Options

`-q` *queryfiles*

Specify the query files containing the queries to match against the indexed database sequences. The names of the query files are separated by white spaces. Each query file can be in FASTA-, EMBL-, SWISSPROT-, or GENBANK format. The format of all files has to be identical. If none of the four formats is used, then the program exits with error code 1. Each file can be in gzipped compressed format, in which case the file must have the ending `.gz`.

`-dnavsprot` *t* [*mapfile*]

Match six frame translation of query sequences against the protein sequence index. First argument *t* specifies the number of the codon translation table used for the six frame translation of the query. *t* must be a number in the range $[1,23]$ except for 7, 8, 17, 18, 19 and 20. Table 3 gives the possible numbers and their names. The codon translation tables were taken from the website `ftp://ftp.ncbi.nih.gov/entrez/misc/data/gc.prt`. The second optional argument is a filename containing a symbol mapping for DNA sequences. This is used for reading the query sequences. If the second argument is missing, then the standard DNA symbolmap is used, as if option `-dna` was used for `mkvtree`. When matching the six frame translation of the query against the index, the symbol mapping is used that was supplied to `mkvtree`, when computing the index.

9.2 Matchkind Options

`-tandem`

Compute branching tandem repeats for an index which only contains database sequences. This option requires the use of the option `-l` to specify the minimum length of the branching tandem repeats to be reported. A tandem repeat is a sequence that occurs more than once in the database sequence at consecutive positions. For a precise definition of the notion branching repeat, see the end of Appendix A.

`-supermax`

Compute supermaximal repeats for an index which only contains database files. This option requires the use of option `-l` to specify the minimum length of the supermaximal repeats to be reported. A repeat is a sequence that occurs more than once in the database sequence. A repeat is supermaximal if it never occurs as a substring of any other maximal repeat. Suppose that a maximal repeat of length l occurs at positions $i_0, i_1, \ldots, i_{k-1}$. Then we report all pairs of positions i_q and i_p such that $q \in [0, k-1]$, $p \in [0, k-1]$, and $i_q < i_p$, together with the usual information as reported for the other kinds of repeats.

Table 5: Overview of the `vmatch`-Options sorted by Categories

	Input parameter
`-q`	specify files containing queries to be matched
`-dnavsprot`	perform six frame translation of DNA
	Kind of matches
`-tandem`	compute right branching tandem repeats
`-supermax`	compute supermaximal matches
`-mum`	compute maximal unique matches
`-complete`	specify that query sequences must match completely
	Postprocessing of matches
`-dbnomatch`	mask all database substrings containing a match
`-qnomatch`	show all query substrings not containing a match
`-dbmaskmatch`	mask all database substrings containing a match
`-qmaskmatch`	mask all query substrings containing a match
`-dbcluster`	cluster the database sequences
`-nonredundant`	generate file with non-redundant set of sequences;
`-pp`	generic postprocessing of matches
`-selfun`	specify shared object file containing selection function
	Algorithms
`-online`	run algorithms online without using the index
`-qspeedup`	specify speedup level when matching queries
	Direction of matches
`-d`	compute direct matches (default)
`-p`	compute palindromic (i.e. reverse complemented matches)
	Match constraints
`-l`	specify that match must have the given length
`-h`	specify the allowed hamming distance > 0
`-e`	specify the allowed edit distance > 0
`-allmax`	show all maximal matches in the order of their computation
`-seedlength`	specify the seed length
`-hxdrop`	specify the xdrop value for hamming distance extension
`-exdrop`	specify the xdrop value for edit distance extension
`-leastscore`	specify the minimum score of a match
`-evalue`	specify the maximum E-value of a match
`-identity`	specify minimum identity of match in range [1..100%]
	Output modes
`-sort`	sort the matches, additional argument is mode
`-best`	show the best matches (those with smallest E-values)
`-i`	give information about number of different matches
	Output formats
`-s`	show the alignment of matching sequences
`-showdesc`	show sequence description of match
`-f`	show filename where match occurs
`-absolute`	show absolute positions
`-nodist`	do not show distance of match
`-noevalue`	do not show E-value of match
`-noscore`	do not show score of match
`-noidentity`	do not show identity of match
	Miscellaneous
`-v`	verbose mode
`-version`	show the version of the Vmatch package
`-help`	show basic options
`-help+`	show all options

`-mum` [*cand*]
Compute maximal substring matches which are unique, both in the the database sequences and in the query sequences. Such matches are called maximal unique matches. If the optional argument `cand` is used, then *MUM*-candidates are computed, i.e. maximal substring matches that are unique in the database but not necessarily in the query sequence. This option requires the use of the option `-l` to specify the minimum length of the matches to be reported. Additionally, the option `-q` is required.

`-complete` [*remred*]
Match each query sequence completely against the indexed database sequences. In this case, a query sequences is also called *pattern*. This option only works in conjunction with option `-q`. The pattern can be a sequence of characters (sequence pattern) over the same alphabet as the database. If this option is used together with the options `-online` and `-e`, then an additional argument `remred` removes redundant matches from the output.

If none of the matchkind options is given, then `vmatch` searches for maximal substring matches.

9.3 Postprocessing Options

`-dbnomatch` *r* [*flag*]
Compute substrings of length at least *r* in the database sequences which are not part of a match computed according to the remaining options. This works as follows: instead of showing the match in the usual way, the positions covered by a match are marked and finally only those substrings which are not marked are output if their length is at least *r*. The optional argument *flag* can be any of the following values:

- `keepleft` means that the left instance of the match is not marked.
- `keepright` means that the right instance of the match is not marked.
- `keepleftifsamesequence` means that the left instance of the match is not marked whenever the right instance of the match occurs in the same sequence.
- `keeprightifsamesequence` means that the right instance of the match is not marked whenever the left instance of the match occurs in the same sequence.

Note that the left instance of a match is always the part of the match in the indexed database sequences. If the index is compared to itself, then right instance of a match is also in the indexed database sequences. If query sequences are matched against the index, then the right instance of a match is in the query sequences. Note that positions that are covered by a match but that are not marked due to the use of one of these flags, may still be missing in the output, because they may be marked by the left or the right instance of a different match. This option was called `-allunique` in a previous version of this program when comparing an index against itself.

`-qnomatch` *r*

Compute substrings of length at least *r* in the query sequences which are not part of a match computed according to the remaining options. The query files containing the query sequences then have been specified with the option `-q` either given to `mkvtree` or to `vmatch`.

`-dbmaskmatch` (`tolower`|`toupper`|*X*) [*flag*]

Compute substring matches but do not output them. Instead, output the complete database sequence in FASTA format and mask the matching substrings. One of the two keywords `tolower` or `toupper` or a single character *X* must be used as an argument. It determines how the matches are masked:

- If the argument to this option is `tolower`, then all characters of the matching substrings are transformed from upper case to lower case. This requires that all characters in the database sequences are in upper case.
- If the argument to this option is `toupper`, then all characters of the matching substrings are transformed from lower case to upper case. This requires that all characters in the database sequences are in lower case.
- If the argument to this option is a single visible character, say *X*, then all characters of the matching substrings are replaced by *X*.

The optional argument *flag* can be any of the following values:

```
keepleft
keepright
keepleftifsamesequence
keeprightifsamesequence
```

If *flag* is used, then it has the same semantics as for option `-dbnomatch`. After showing all masked sequences, and extra line reports the number of symbols masked. This line is shown on stderr. If the environment variable `VMATCHCOMMENTTOSTDOUT` is set to the value `on`, then the line is shown on stdout.

`-qmaskmatch` (`tolower`|`toupper`|*X*)

Compute substring matches but do not output them. Instead, output the complete query sequence in FASTA format and mask the matching substrings. This option requires the option `-q` specifying query files. The additional argument specifies how the matches are masked. The semantics is as in option `-dbmaskmatch`.

`-dbcluster` p_{small} p_{large} [*filenameprefix*] ($(minclsize, maxclsize)$)]

Cluster the database sequences according to the matches found in a self comparison of the index. p_{small} and p_{large} are integers in the range $[0,100]$. Initially each database sequence is put into its own cluster. Then the matches are computed, but they are not shown. Instead, they are evaluated to form single linkage clusters. In particular, consider a match

between two database sequences, where the smaller of these sequences is of length ℓ_{small} and the larger of these sequences is of length ℓ_{large}. Whenever both match instances cover at least $p_{small}\%$ of the smaller sequence and $p_{large}\%$ of the larger sequence (or equivalently when the smaller of both match instances is of length $(\ell_{small} \cdot p_{small})/100$ and the larger is of length $(\ell_{large} \cdot p_{large})/100$), then the clusters containing these two sequences are joined. After computing all matches, the clusters are output. The given cluster numbers are consecutive numbers beginning with 0. Suppose that the argument *filenameprefix* is specified. Then additional information about the clusters is reported:

- For each cluster, say with number i, a file *filenameprefix*`.`s`.`i`.match` is generated storing all matches which were used to form cluster i. s is the size of the cluster with number i. The matches are reported in the same format as described in Section 9.9, and the format can be modified by the corresponding output options. However, note that the option `-s` does not give you the alignment in the `.match`-file. To obtain this, one can apply the program `vmatchselect` to the matchfile. `vmatchselect -s` gives the alignment for each sequence involved in a match.
- If the option `-s` (see below) is specified, then for each cluster, say with number i, a file named *filenameprefix*`.`s`.`i`.fna` is generated. s is the size of the cluster, and the file stores all sequences contained in cluster i in multiple FASTA format. This includes the description of the sequence prefixed by the sequence number.

If the third optional argument is used, then there may be a fourth optional argument of the form $(minclsize, maxclsize)$. *minclsize* and *maxclsize* are integers satisfying the following: $minclsize \geq 1$ and $minclsize \leq maxclsize$ or $maxclsize = 0$. If $maxclsize = 0$, then this is interpreted as ∞. If *minclsize* and *maxclsize* are specified, then the `.match` and `.fna` file for a cluster are only output if the cluster has at least *minclsize* and at most *maxclsize* elements. The singleton sequences of the index, i.e. those sequences not contained in any cluster are output in a single file named *filenameprefix*`.single.fna` if $minclsize = 1$ and if the option `-s` is used. Note that in some Unix-shells the symbols `(` and `)` have special meaning. So it may be necessary to quote the argument $(minclsize, maxclsize)$, so that the shell does not evaluate it. For example, single quotes work for the C-Shell and the Bourne-Shell.

Note that most of the options determining the matches to search for, can be combined with the option `-dbcluster`. The corresponding holds for the options determining the output format of the matches. Note also that the option `-s` has a slightly different meaning when used in combination with `-dbcluster`. It produces the additional `.fna` files containing the sequences of a cluster.

`-nonredundant` *filename*

Output a set of non-redundant sequences stored in the index. The output is in FASTA format and goes to file *filename*. The option only works in combination with option `-dbcluster`. The non-redundant sequences are determined in the following way: All sequences are clustered according to the given options. For each cluster we output the longest sequence from that cluster, including its description, to obtain a FASTA formatted file.

`-pp` *`chain`* *chain-parameter*

Specify that matches should be chained. *chain-parameter* is a sequence of the following keywords (each optionally followed by some value):

Keyword	meaning
`global`	global chaining
`local`	local chaining
`wf`	specify weight factor
`maxgap`	specify maximal gap width
`outprefix`	specify prefix of output files

When prepending the symbol − to a keyword, one obtains an option of the separate program *chain2dim*. All strings in *chain-parameters* that are not keywords, are considered to be arguments of the preceeding option. For example,

```
-pp chain local 10p wf 1.8 maxgap 10
```

is translated into the options

```
-local 10p -wf 1.8 -maxgap 10
```

of the program *chain2dim*. This program is part of the *Vmatch*-distribution and its options are described in a separate manual. We refer to this *chain2dim*-manual for a comprehensive description of these options and the meaning of the five keywords in turn. Note that *chain2dim* reads the matches from a match file before it does the chaining. In contrast `vmatch` computes matches, stores them internally, and then performs chaining.

`-pp` *`matchcluster`* *matchcluster-parameter*

Specify that matches should be clustered. *matchcluster-parameter* is a sequence of the following keywords (each optionally followed by some value):

Keyword	meaning
`erate`	specify maximum error rate for similarity clustering
`gapsize`	specify maximum gapsize for gap clustering
`overlap`	specify minimum percentage of overlap for overlap clustering
`outprefix`	specify prefix of output files

When prepending the symbol − to a keyword, one obtains an option of the separate program *matchcluster*. All strings in *matchcluster-parameter* that are not keywords, are considered to be arguments of the preceeding option. For example,

```
-pp matchcluster erate 35 outprefix clout
```

is translated into the options

`-erate 35 -outprefix clout`

of the program *matchcluster*. This program is part of the *Vmatch*-distribution and its options are described in a separate manual. We refer to this *matchcluster*-manual for a comprehensive description of these options and the meaning of the four keywords in turn. Note that *matchcluster* reads the matches from a match file before it computes clusters. In contrast `vmatch` computes matches, stores them internally, and then performs the clustering step.

`-selfun` *filename* [*extraargs*]
Access file *filename*, which must be a shared object file containing a selection function bundle. If the access to such a selection function bundle is not possible, then the program terminates with error code 1. If *filename* is not an absolute path (i.e., it does not begin with a forward slash (‘/’), then the file is searched for in the colon-separated list of directories specified by the environment variable `LD_LIBRARY_PATH`. For details on selection functions see Section E. *filename* must have the appropriate extension. This depends on the particular platform. For most Unix-platforms the extension `.so` is valid. For HP-UX, the valid file extension is `.sl`. Option `-filename` can be followed by an optional list *extraargs* of extra arguments (not beginning with the symbol `-`). These are ignored by `vmatch` and `vmatchselect`. Since they are stored in the argument vector passed to the function `selectmatchHeader` (see Section E), they can be evaluated in the selection function bundle.

9.4 Algorithm Options

`-online`
Run online algorithms where possible. These do not use the index, except for the original and the transformed input sequences, and are therefore more space efficient. However, the online algorithms usually run not as fast as the index based algorithms. We currently provide this option for three different matching tasks:

- match a substring of the query sequences against an index with or without allowing for errors.
- match each query sequence completely against an index with or without allowing for errors

This option only works together with option `-q`.

`-qspeedup` *i*
`vmatch` provides two algorithms to compute maximal exact substring matches of a data base and a query. We may later add more algorithms. The output of the algorithms are

identical. You can select each of these algorithms, termed Algorithm i for $i \in \{0,2\}$ by the appropriate choice of i:

- Algorithm 0 is the most space efficient algorithm. If there are not many long exact matches between the query sequences and the database sequences, then the algorithm is about as fast as Algorithm 2.
- Algorithm 1 is no longer available.
- Algorithm 2 requires n bytes more space than Algorithm 0. If there are many long exact matches between the query sequences and the database sequences, then this Algorithm is faster than Algorithm 0.

These algorithms are applied only if you specify query files using the option `-q`, or if you are searching for palindromic matches in a self comparison of the index. The default algorithm is Algorithm 2.

9.5 Direction Options

`-d`

Report direct matches. This option can be combined with option `-p`.

`-p`

Report palindromic matches in case one is searching DNA sequences. This option can be combined with option `-d`. If neither option `-d` nor `-p` is used, then only direct matches are reported.

9.6 Matchconstraint Options

`-l` ℓ [*mingapsize*] [*maxgapsize*]

Specify the length value ℓ. This must be a positive integer. Only matches of length at least ℓ are reported. The optional arguments *mingapsize* and *maxgapsize* can be specified when searching repeats. *mingapsize* and *maxgapsize* are (possibly negative) integers specifying the minimum and the maximum gap size between the two instances of a repeat. If *maxgapsize* is specified, then also *mingapsize* must be specified. If *maxgapsize* is not specified, then no upper bound on the gap size is specified. Both optional arguments can be negative specifying that the repeat instances may overlap. For an explanation of the effect of these optional arguments, consider a repeat specified by four integers (l,i,r,j). l is the length of first instance of the repeat. r is the length of second instance of the repeat. i is the start position of the left instance, and j is the start position of the right instance of the repeat. Note that $i < j$. If $i+l-1 > j$, then the gapsize g of the repeat is $-(i+l-j)$. If $i+l-1 \leq j$, then the gapsize g of the repeat is $j-(i+l-1)$. The repeat is accepted if $g \geq$ *mingapsize* and $g \leq$ *maxgapsize*, provided *maxgapsize* is defined.

`-h` *k*

Specify the allowed hamming distance > 0. This is the hamming distance option, which only allows mismatches in matches. If this option is used together with option `-l`, then substring matches are sought such that the two instances of the match contain at most k mismatches. Here the mandatory argument k is a positive integer. If this option is used together with option `-complete`, then complete matches are sought, and the mandatory argument k specifies the number of mismatches allowed in the match. k can have three different forms:

- If k is a positive integer, then this specifies the absolute number of mismatches allowed.
- If k is of the form *i*`p`, where i is a positive integer, then up to $\frac{i}{100} \cdot m$ mismatches are allowed in a match. Here m is the length of the current query to be searched. Thus the character `p` specifies the mismatch rate as a percentage of the query length. This mode is called the *percentage* search mode.
- If k is of the form *i*`b`, where i is a positive integer, then in a first search phase, the minimum number, say q, of mismatches is determined, which still delivers at least one match for the given query. Then all matches with exactly q mismatches are reported. The range of values for q to try in the first phase can be controlled by the positive integer. That is, q must be smaller than or equal to $\frac{i}{100} \cdot m$. This mode is called the *best* search mode.

`-e` *k*

Specify the allowed edit distance > 0. This is the edit distance option, which allows differences, i.e. mismatches, insertions, and deletions in matches. If this option is used together with option `-l`, then substring matches are sought such that the two instances of the match contain at most k differences. Here the mandatory argument k is a positive integer. If this option is used together with option `-complete`, then complete matches are sought, and the mandatory argument k specifies the number of differences allowed in the match. k can have three different forms:

- If k is a positive integer, then this specifies the absolute number of differences allowed.
- If k is of the form *i*`p`, where i is a positive integer, then up to $\frac{i}{100} \cdot m$ differences are allowed in a match. Here m is the length of the current query to be searched. Thus the character `p` specifies the difference rate as a percentage of the query length. This mode is called the *percentage* search mode.
- If k is of the form *i*`b`, where i is a positive integer, then in a first search phase, the minimum number, say q, of differences is determined, which still delivers at least one match for the given query. Then all matches with exactly q differences are reported. The range of values for q to try in the first phase can be controlled by the positive integer. That is, q must be smaller than or equal to $\frac{i}{100} \cdot m$. This mode is called the *best* search mode.

Be very careful when you choose the parameter k and do not use the option `-complete`. The running time of the program is very dependent on k. More precisely, it grows faster than $k^2 l$. A reasonable value for k is in the range $[1,10]$ (as was restricted in earlier program versions).

`-allmax`

Report all maximal matches, in the order in which they are found. We do not recommend to use this option. It is mainly used for compatibility with the program *REPuter*.

`-seedlength` m

Set the length of the exact seeds. This option is only allowed together with one of the options `-e`, `-h`, `-exdrop`, or `-hxdrop`. If options `-e` or `-h` is used, then the seed length is the maximum of $\lfloor \ell/(k+1) \rfloor$ and m, where the argument m is a positive integer, k is the argument for options `-e` or `-h`, respectively, and ℓ is the argument for option `-l`. If options `-exdrop` or `-hxdrop` is used, then the seed length is m. The user should carefully consider the choice for m if using either option `-e` or `-h`: if m is larger than $\lfloor \ell/(k+1) \rfloor$, `vmatch` may miss some matches of length at least ℓ with the specified number k of errors. If the options `-exdrop` or `-hxdrop` is used, but not `-seedlength`, then the default value for the seed length is 30.

`-hxdrop` X

Specify the *Xdrop*-score X when extending a seed in both directions allowing only for matches and mismatches. The argument X must be a positive integer smaller or equal to 255. Matches are scored 2 and mismatches are scored -1. The extension process stops as soon as the extension involving matches and mismatches has a score smaller than $T-X$ where T is the largest score seen so far. This option requires that one of the options `-l`, `-leastscore`, `-evalue` or `-identity` is used to restrict the matches. The minimum length of the seeds is specified by the argument to option `-seedlength`. If this option is not used, then the default value for the seed length is 30.

`-exdrop` X

Specify the *Xdrop*-score X when extending a seed in both directions allowing for matches, mismatches, insertions, and deletions. The argument X must be a positive integer smaller or equal to 255. Matches are scored 2, mismatches are scored -1, and indels are scored -2. The extension procedure is further explained in Appendix C. This option requires that one of the options `-l`, `-leastscore`, `-evalue` or `-identity` is used. The minimum length of the seeds is specified by the argument to option `-seedlength`. If this option is not used, then the default value for the seed length is 30.

`-leastscore` ls

Specify the least score ls a match must have to be reported. That is, if an optimal alignment of the matching sequences has score smaller than the positive integer ls, then it is not reported. The score of the alignment is computed from the match/mismatch/indel scores as defined above.

`-evalue` *e*

Specify the maximum E-value e a match must have to be reported. That is, if a match has an E-value larger than the floating point value e, then it is not reported. The argument e to this option must either be 0.0 or $\geq 1.0 \cdot 10^{-300}$.

`-identity` *q*

Specify the minimum identity value q a match must have to be reported. The identity value of a match is defined by $100 \cdot (1 - \frac{d}{l})$ where l is the maximum of the lengths of the substrings s_1 and s_2 involved in the match and d is the number of mismatches and indels in the optimal alignment of s_1 and s_2. Thus, if the identity value of the match is strictly smaller than the positive integer q, then it is not reported.

9.7 Output Options

`-sort` *mode*

sort the reported matches according to the given mode. The following modes are available:
`la`: sort in ascending order of length
`ld`: sort in descending order of length
`ia`: sort in ascending order of first position
`id`: sort in descending order of first position
`ja`: sort in ascending order of second position
`jd`: sort in descending order of second position
`ea`: sort in ascending order of E-value
`ed`: sort in descending order of E-value
`sa`: sort in ascending order of score
`sd`: sort in descending order of score
`ida`: sort in ascending order of identity
`idd`: sort in descending order of identity

If used for `vmatch`, then this option requires to also use the option `-best`.

`-best` *m*

Report at most the best m matches. m is a positive integer. The matches are reported according their E-value, their length, and their start position (in this order). That is, if two matches are compared, the most important value is their E-value. The match with the smaller E-value is "better". If both matches have the same E-value, then the longer match is "better". If both matches have the same E-value and are of the same length, then their first start position matters, and the matches with the smaller start position is "better". The reported matches satisfy the given length and error constraints.

`-i`

Do not show matches, but a distribution (preview `i`nformation) about the length of the different matches found.

`-s` [*q*] [*flag*]

Additionally report an alignment of the two instances of the match. The optional integer argument *q* specifies the linewidth. That is, the alignments or matching substrings are formatted to *q* symbols per line. If *q* is not specified, then the default linewidth is 60. For details see Section 9.9. The optional argument *flag* can be any of the following five values:

- `abbrev` means that the matching substrings of an exact match or a hamming distance match are shown in abbreviated form. That is, the exact matching substring is shown verbatim, while for a hamming distance match a mismatching pair of characters, say *a* in the left instance of the match and *c* in the right instance of the match, are shown as [*ac*]. The output of an edit distance match is not affected by this flag.
- `abbreviub` has the same meaning as `abbrev`, except that each pair of different symbols in a match containing only mismatches is shown as a single IUB-character, according to the following table:

	A	C	G	T
A		*m*	*r*	*w*
C	*m*		*s*	*y*
G	*r*	*s*		*k*
T	*w*	*y*	*k*	

  Note that a mismatch of a wildcard character, say *n*, with a symbol *a* is still reported as [*na*] or [*an*] since there is no IUB-character for denoting the set consisting of *a* and *n*. The output of an edit distance match is not affected by this flag.
- `leftseq` means that only the sequence information of the left instance of a match is reported.
- `rightseq` means that only the sequence information of the right instance of a match is reported.
- `xml` means that the entire output is in XML-format, see an example in section 9.9.2.

`-showdesc` *m*

Instead of the sequence number, show the descriptions of the sequences. The mandatory argument *m* can have three forms:

- If *m* is a positive integer, then up to the first *m* symbols of the descriptions of the sequences are shown.
- If *m* is 0, then the descriptions of the sequences is shown up to the first white space character. If there is no such character, then the complete description is shown.
- If *m* is of the form `(`*skipprefix*`,`*maxlength*`)` where *skipprefix* and *maxlength* are non-negative integers, then the initial *skipprefix* symbols of the sequence descriptions are skipped. Moreover, if *maxlength* > 0, then up to *maxlength* remaining symbols are shown. If *maxlength* is 0, then the remaining symbols of the description are shown

up to first white space character. If there is no such white space character, then the remaining part of the description is shown.

White spaces in the descriptions are replaced by the symbol _, so that each description is shown in a single column of the output. This simplifies parsing of the output file.

`-f`
Additionally report the filename the match instance is contained in.

`-absolute`
Show the absolute positions instead of pairs of sequence numbers and relative positions.

`-nodist`
Do *not* show the distance value of a match.

`-noevalue`
Do *not* report the E-value of a match.

`-noscore`
Do *not* report the score of a match.

`-noidentity`
Do *not* report the identity value of a match.

The previous four options reduce the size of the output considerably, and in a lot of cases also the running time of the program. This is especially true when comparing an index against itself. Then the matches are computed so fast that the generation of the output takes a considerable share of the total running time. Note however, that the options `-noevalue` and `-nodist` produce output that cannot parsed by the program `vmatchselect`.

9.8 Miscellaneous Options

`-v`
Be verbose, that is, give reports about the different steps as well as the resource requirements of the computation. Additionally, it produces an initial explanation of the format in which the matches are shown.

`-help`
Show a summary of all basic options and terminate with exit code 0.

`-help+`
Show a summary of all options and terminate with exit code 0.

Note the following when combining options of `vmatch`:

1. Option `-seedlength` can only be used in combination with exactly one of the options `-h`, `-e`, `-hxdrop`, and `-exdrop`.
2. Option `-online` only works together with option `-q`.
3. Option `-complete` only works together with option `-q`.
4. Option `-mum` only works together with option `-l`.
5. Option `-super` only works together with option `-l`.
6. Option `-tandem` only works together with option `-l`.
7. Option `-allmax` only works together with either option `-h` or option `-e`.
8. Option `-nonredundant` only works together with option `-dbcluster`.
9. Option `-qspeedup` only works together with either option `-query` or option `-p`.
10. Options `-hxdrop` and `-exdrop` can only be used in combination with at least one of the options `-l`, `-leastscore`, `-identity`, or `-evalue`.
11. If option `-complete` is not used, then either option `-l` or `-exdrop` or `-hxdrop` must be used.
12. If option `-complete` is not used, then options `-e` or `-h` require to use option `-l`.

Table 6 shows all 102 combinations of options that cannot be pairwise combined.

If the options `-best`, `-allmax`, and `-complete` are not used, then for each seed a best match, i.e. one with a minimum E-value is output. The matches are enumerated in the order as they are found. There is no limit on the number of matches reported.

9.9 Applying `vmatch`

9.9.1 Self Comparison

Using the index `atEST` generated by `mkvtree` (see Section 2.2), we perform a self comparison of the database sequences, searching for exact duplicates of length at least 350 in `atEST`:

```
$ vmatch -v -l 350 atEST
# args=-v -l 350 atEST
# matches are reported in the following way
# l(S) n(S) r(S) t l(S) n(S) r(S) d e s i
# where:
# l = length
# n = sequence number
# r = relative position
# t = type (D=direct, P=palindromic)
```

Table 6: Options of `vmatch` that cannot be combined with each other. The entries are meant to be symmetric. That is, if option *a* cannot be combined with option *b*, then option *b* cannot be combined with option *a*.

option	cannot be combined with option
-dnavsprot	-supermax -tandem -dbcluster -nonredundant
-online	-supermax -tandem -dbcluster -nonredundant
-l	-complete
-q	-supermax -tandem -dbcluster -nonredundant
-complete	-dbcluster -nonredundant -allmax -mum -supermax -tandem -seedlength -hxdrop -exdrop -qspeedup
-mum	-supermax -tandem
-supermax	-tandem -p -qspeedup
-tandem	-allmax -h -e -hxdrop -exdrop -qnomatch -qmaskmatch -p -qspeedup -dbcluster -nonredundant
-i	-dbcluster -nonredundant -pp -sort -showdesc -absolute -f -dbnomatch -dbmaskmatch -qmaskmatch -qnomatch -nodist -noevalue -noscore -noidentity -s
-h	-e -hxdrop -exdrop
-e	-hxdrop -exdrop
-hxdrop	-exdrop
-allmax	-best -sort -hxdrop -exdrop -leastscore -evalue -identity
-showdesc	-absolute
-dbnomatch	-nodist -noevalue -noscore -noidentity -dbcluster -nonredundant -dbmaskmatch -qmaskmatch -qnomatch -pp
-qnomatch	-nodist -noevalue -noscore -noidentity -dbcluster -nonredundant -pp
-dbmaskmatch	-nodist -noevalue -noscore -noidentity -dbcluster -nonredundant -qmaskmatch -pp
-qmaskmatch	-nodist -noevalue -noscore -noidentity -dbcluster -nonredundant -pp
-pp	-sort

```
# d = distance value (negative=hamming distance, 0=exact, positive=edit distance)
# e = E-value
# s = score value (negative=hamming score, positive=edit score)
# i = percent identity
# (S) = in Subject
# file=atEST 999815 772376
# databaselength=772375 (including 1951 separators)
# sequence lengths: minimal=56, maximal=1102, average=394.68
# alphabet of size 5: acgtn
# atEST.tis read
# atEST.bwt read
# atEST.suf read
# atEST.lcp read
# atEST.llv read
# atEST.ssp read
# find direct substring matches (repeats)
  388      79    236   D   388    1495    180   0   4.22e-223    776   100.00
  517      64      0   D   517      65      0   0    0.00e+00   1034   100.00
  392     229      0   D   392     270      0   0   1.65e-225    784   100.00
  369     736    141   D   369    1151     85   0   1.16e-211    738   100.00
  402      78    363   D   402    1488    146   0   1.57e-231    804   100.00
  369     902     54   D   369    1151     87   0   1.16e-211    738   100.00
  367     736    143   D   367     902     54   0   1.86e-210    734   100.00
# overall space peak: main=0.07 MB (0.09 bytes/symbol), secondary=5.18 MB (7.03 bytes/symbol)
```

In all examples, the symbol `$` is the prompt, after which user input consisting of the called program plus the arguments are shown. In the line beginning with `# args=`, the arguments of `vmatch` are echoed. Additionally, the verbose option gives us the remaining lines marked by the symbol `#`. They explain, in abbreviated form, the meaning of the different columns. Each of the remaining lines, if not marked by the symbol `#` in the first column, reports a match by showing the following items from left to right:

(1) The length of the left instance of the match.

(2) The number, say i, of the input sequence, the left instance of the match occurs in. The input sequence numbers are counted from 0.

(3) The relative position of the left instance of the match in sequence i.

(4) A character `D` for direct matches and a character `P` for palindromic matches.

(5) The length of the right instance of the match.

(6) The number, say j, of the input sequence, the right instance of the match occurs in. The input sequence numbers are counted from 0. If the right instance occurs in a query sequence, then j is relative to the first query sequence.

(7) The relative position of the right instance of the match in sequence j.

(8) The distance of the match. An exact match has distance 0. A k-mismatch match with $k > 0$ mismatches has distance $-k$. A k-differences match with $k > 0$ differences has distance k.

(9) The E-value of the match.

(10) The score of the match. The score is computed from the optimal alignment of the left and the right instance of the match. Matching characters in the alignment are scored 2, mismatching characters are scored -1, and indels (i.e. insertions and deletions) are scored -2.

(11) The identity value $100 \cdot (1 - \frac{d}{\max\{l_1, l_2\}})$ of the match where l_1 and l_2 are the lengths of the match instances s_1 and s_2 and d is the number of mismatches and indels in the optimal alignment of s_1 and s_2.

Now let us search for palindromic matches with a minimum length of 200, but allowing for up to one mismatch. Instead of a sequence number we output the corresponding sequence description using the option `-showdesc 10`. We additionally report the alignments of the different matches. This alignment is formatted to 60 characters per line. The matching sequences (on the `Sbjct`-lines) only contain a few mismatches. Each such mismatch between, say character c_1 in the left instance of the match (upper line) and character c_2 in the right instance of the match (lower line), is emphasized by the symbol `!`.

```
$ vmatch -p -l 200 -h 1 -showdesc 10 -s 60 atEST
# args=-p -l 200 -h 1 -showdesc 10 -s 60 atEST
  218   gi|3719113      0   P   218   gi|3450463     75  -1   6.18e-118   -433    99.54
Sbjct: TGAATTGGCAAAGTCTATAAAAAGACCCAAAAATAATACAATGAAAAGGAGAAAAGACAG          60
Sbjct: TGAATTGGCAAAGTCTATAAAAAGACCCAAAAATAATACAATGAAAAGGAGAAAAGACAG         135

Sbjct: AAGCAAATATTGGAGATCACTAAGATGCGCATGTTGATCACTGCTGGGCACATTGCACAC         120
Sbjct: AAGCAAATATTGGAGATCACTAAGATGCGCATGTTGATCACTGCTGGGCACATTGCACAC         195

Sbjct: GCTGAGCACCGCCTGGGTGATCTTCCTCATCGTCATCATAAGCCTCTCTTTGAGCTTGCG         180
Sbjct: GCTGAGCACCGCCTGGGTGATCTTCCTCATCGTCATCATAAGCCTCTCTTTGAGCTTGCG         255

Sbjct: CCTTCCTTTTCATCTCATCCTCAATGTTCACATCATGC                               218
                                    !
Sbjct: CCTTCCTTTTCATCTCATCCTCAATGGTCACATCATGC                               293


  209   gi|3719089      0   P   209   gi|3450056    231   0   2.48e-115    418   100.00
Sbjct: AAAAGTTTTGAAACTCTTCTATACACATACATTCTCCGGATGTGGTTGTTACTAACTTCA          60
Sbjct: AAAAGTTTTGAAACTCTTCTATACACATACATTCTCCGGATGTGGTTGTTACTAACTTCA         291

Sbjct: AAATATAAAAATTTAACAAAACAATTGTTATCATCATTTCCTACGAGTCATTAAACCCAA         120
Sbjct: AAATATAAAAATTTAACAAAACAATTGTTATCATCATTTCCTACGAGTCATTAAACCCAA         351

Sbjct: TCCCACTCGCCGTCGCCGGAAAACACCTCGGAAAATCAGCCACCGAAAACGATCTCCAGG         180
Sbjct: TCCCACTCGCCGTCGCCGGAAAACACCTCGGAAAATCAGCCACCGAAAACGATCTCCAGG         411

Sbjct: CACAAAAACCCAACGACGACTGAGACGAA                                        209
Sbjct: CACAAAAACCCAACGACGACTGAGACGAA                                        440
```

We only show the initial first 10 characters of the description. Note that this time we did not use the verbose option. The output contains one match with a single mismatch and one exact match.

Instead of an alignment, we can also report both sequences involved in the match:

```
$ vmatch -p -l 230 -hxdrop 3 -showdesc '(3,7)' -s abbrev atEST
# args=-p -l 230 -hxdrop 3 -showdesc (3,7) -s abbrev atEST
  231   3719145    131   P   231   3719112     80 -12   2.70e-103   -426    94.81
AA[TA][CT]C[TC]TT[GT][AG]A[TA][GT]GGGAGAA[NG]ATAAAGAGAGTCAC[
GV]ATATGCTTAGACAAGG[AT]TTCACTCATGCATTTTCGATGACCTTTGAGAACAAAG
ATGGTTACGTCGCCTTCACAAGCCATCCTCTTCATGTTGAATTCTCA[NG]CCGCTTTCA
CCGCCGTCATCGACAA[NG]ATCGTTCTCCTCGATTTCCCCGTCGCCGCTGTCAAATCTT
CCGTTGTTGCAACACCATGAATCTTGT
```

Here each mismatch between, say c_1 in the left instance of the match and c_2 in the right instance of the match, is reported as $[c_1c_2]$. Also note, that we use option `-showdesc '(3,7)'` to skip the first 3 characters of the description and to show only the first 7 characters from the beginning of the rest. As an alternative we can use option `-s abbreviub` to report a mismatch of c_1 and c_2 as a single IUB character (provided both c_1 and c_2 are not wildcard symbols).

```
$ vmatch -p -l 230 -hxdrop 3 -showdesc '(3,7)' -s abbreviub atEST
# args=-p -l 230 -hxdrop 3 -showdesc (3,7) -s abbreviub atEST
  231   3719145    131   P   231   3719112     80 -12   2.70e-103   -426    94.81
AAWYCYTTKRAWKGGGAGAA[NG]ATAAAGAGAGTCAC[GV]ATATGCTTAGACAAGGWT
TCACTCATGCATTTTCGATGACCTTTGAGAACAAAGATGGTTACGTCGCCTTCACAAGCC
ATCCTCTTCATGTTGAATTCTCA[NG]CCGCTTTCACCGCCGTCATCGACAA[NG]ATCG
TTCTCCTCGATTTCCCCGTCGCCGCTGTCAAATCTTCCGTTGTTGCAACACCATGAATCT
TGT
```

Instead of showing the two matching sequences at once, we can report the left or the right instance of the the match, using the argument *leftseq* or *rightseq*. This also works for inexact matches:

```
$ vmatch -p -l 379 -exdrop 3 -s 60 leftseq atEST
# args=-p -l 379 -exdrop 3 -s 60 leftseq atEST
>  379     299      0   P   387     883     53  22   1.14e-168    700    94.32
AAAAGTTTTGAAACTCTTCTATACACATACATTCTCCGGATGTGGTTGTTACTAACTTCA
AAATATAAAAATTTAACAAAACAATTGTTATCATCATTTCCTACGAGTCATTAAACCCAA
TCCCACTCGCCGTCGCCGGAAAACACCTCGGAAAATCAGCCACCGAAAACGATCTCCAGG
CACAAAAACCCAACGACGACTGAGACGAATAAATTACCAAAAGACCCTTGACTCCTAGGA
TACAACGGAGGAAGCTTCTTCATCTGATGTTGATGATGATGTAGCTGCCTCGGAGGAGAT
CCAGAAGTCGTCGGWYTTGATCCCGGCGACGAGTCACCTCCGGAAGAAGATCCTTCTCCG
GTCATCACACCAGCGGCAA
```

Now consider an example where we combine the search for direct and palindromic matches. At most 4 differences between the matches are allowed.

```
$ vmatch -d -p -l 300 -e 4 -showdesc 10 atEST
# args=-d -p -l 300 -e 4 -showdesc 10 atEST
  407   gi|4239690    217   D   407   gi|3449444    161   2   1.46e-227    808    99.51
  351   gi|4714047    172   D   349   gi|4714044    173   4   3.05e-188    688    98.86
  341   gi|4714044    181   D   341   gi|4714011    252   4   2.85e-182    670    98.83
  331   gi|3450456     49   D   328   gi|3450037     75   3   4.62e-179    650    99.09
  331   gi|3450456     49   D   328   gi|3449788    108   3   4.62e-179    650    99.09
  328   gi|3450488     88   D   331   gi|3450456     49   3   4.62e-179    650    99.09
  331   gi|3450456     49   D   328   gi|3450203    164   3   4.62e-179    650    99.09
  324   gi|3450456     56   D   322   gi|3450093    276   4   3.99e-172    634    98.77
  300   gi|3449391      0   D   300   gi|3449385     49   3   1.59e-160    591    99.00
  430   gi|4714045    219   D   430   gi|4714034    337   4   1.89e-235    848    99.07
  300   gi|3719179    121   P   301   gi|3719092      0   4   2.09e-158    589    98.67
  314   gi|3719089      0   P   317   gi|3450056    123   4   5.98e-168    619    98.74
```

Note that, except for option `-p`, all previously used options for `vmatch` can also be used for protein sequences. Here is an example involving the index `Proteinseq`, we constructed earlier:

```
$ vmatch -s -l 50 -exdrop 2 Proteinseq
# args=-s -l 50 -exdrop 2 Proteinseq
57    0   1   D 56    1   2   3    2.36e-47 104    94.74
Sbjct: PTFFLLPPGEGGAESYFNNIVKR-RQTNMFILNNYYFHSKRIRTIEELAEMYLDQVRG            58
         =         !!         !     ===    =    =  =
Sbjct: PTLFLLPPGEGG--SYFNNIVKRLRQTNMVVFNNYYLHSKRLRTFEELAEMYLDQVRG            58
```

Note that, in the alignment, the line between the matching sequences contains the symbols `!` and =. As usual, the symbol `!` emphasizes mismatches, or insertions, or deletions in the corresponding alignment column. The symbol `!` emphasizes columns with different characters, that are equivalent according to the given symbol mapping. For example, the symbols `F` and `L` in column 3 are equivalent according to the symbol map `TransProt11`.

Consider some more examples on the computation of maximal repeats for the *Ecoli O157:H7* genome, for which we have earlier constructed an index. To determine the appropriate parameter set for computing matches, we recommend to start with the option `-i`. This reports the distribution of the length of the matches, but not the match positions or matching sequences.

```
$ vmatch -i -l 12 EcoliO157H7
# args=-i -l 12 EcoliO157H7
# all 1507204
# 12 1066546
# 13 301935
# 14 88735
# 15 27097
# 16 8845
# 17 3753
# 18 1693
# 19 1088
# 20 895
# 21 476
# 22 537
# 23 528
# 24 323
# 25 200
# 26 517
# 27 136
# 28 180
# 29 278
```

This output shows the distribution of the length of all matches of length at least 12. The line starting with the keyword `all` reports the total number of the maximal repeats of length ≥ 12. Then each line of the form

ℓ k

reports that there are k direct repeats of length exactly ℓ. The output (of which only the first 20 lines are shown), reports that there are more 1 million exact repeats of length 12, and 301935 exact repeats of length 13. We conclude that the minimum length of 12 or 13 is probably not selective enough to compute repeats in the *Ecoli O157:H7* genome. A minimum length value of 15 or larger seems to be more appropriate.

We can also quickly get an overview of the number of very long repeats. For example, let us compute the number of matches of length at least 4000 with exact seeds of length 30 (the default seed length) and identity of 85% or more.

```
$ vmatch -i -exdrop 5 -l 4000 -identity 85 EcoliO157H7
# args=-i -exdrop 5 -l 4000 -identity 85 EcoliO157H7
# all 223
# 4113 3
# 4289 29
# 4884 36
# 5446 39
# 5480 19
# 6147 16
# 7494 21
# 7753 46
# 83654 14
```

Instead of restricting the length of the matches, we can restrict the E-value. In the following example, we compute all matches with seed length 20 and E-value smaller than 10^{-100}:

```
$ vmatch -seedlength 20 -exdrop 5 -evalue 10e-100 EcoliO157H7
# args=-seedlength 20 -exdrop 5 -evalue 10e-100 EcoliO157H7
 2793    0 1654897   D  2812    0 1725693 249    0.00e+00   4858    91.15
 1840    0 1280857   D  1848    0 1725693  89    0.00e+00   3421    95.18
 7753    0 1272934   D  7773    0 1647113 496    0.00e+00  14038    93.62
 2013    0 4746246   D  2017    0 4842428  60    0.00e+00   3850    97.03
 3212    0 1267826   D  3216    0 1862616  82    0.00e+00   6182    97.45
 2294    0 1641528   D  2278    0 1862438 297    0.00e+00   3681    87.05
 2439    0 2688397   D  2440    0 2909349 108    0.00e+00   4555    95.57
  395    0 2688268   D   396    0 3492312 123    0.00e+00    422    68.94
  502    0 1639778   D   513    0 1860288  94   1.20e-117    733    81.68
  451    0 1251809   D   449    0 1628124  79   3.46e-107    663    82.48
 6147    0 1287968   D  6141    0 1917801 828    0.00e+00   9804    86.53
  665    0  922807   D   670    0 1290526 247    0.00e+00    594    63.13
```

Now suppose we want to compute only the best 10 palindromic repeats, ordered in descending order of their length. We also want to allow a few errors in the repeats.

```
$ vmatch -p -l 1000 -best 10 -exdrop 1 -sort ld EcoliO157H7
# args=-p -l 1000 -best 10 -exdrop 1 -sort ld EcoliO157H7
 9441    0 1904945   P  9441    0 2124427  12    0.00e+00  18846    99.87
 3873    0 1335481   P  3873    0 2949532  33    0.00e+00   7647    99.15
```

```
 2541    0 1251817   P  2541    0 2726937  12    0.00e+00   5046    99.53
 2447    0 1070054   P  2447    0 2754267   7    0.00e+00   4873    99.71
 2447    0 1465661   P  2447    0 2754267   7    0.00e+00   4873    99.71
 2446    0  345353   P  2446    0 1070055   7    0.00e+00   4871    99.71
 2446    0  345353   P  2446    0 1465662   7    0.00e+00   4871    99.71
 2442    0 1070055   P  2442    0 4597374   4    0.00e+00   4872    99.84
 2442    0 1465662   P  2442    0 4597374   4    0.00e+00   4872    99.84
 2191    0 1923669   P  2191    0 2112243  36    0.00e+00   4274    98.36
```

The following example shows how to restrict the computation to supermaximal repeats (which only works for direct repeats):

```
$ vmatch -supermax -l 2000 EcoliO157H7
# args=-supermax -l 2000 EcoliO157H7
 5031    0 1106367   D  5031    0 1501974   0    0.00e+00  10062   100.00
22545    0 1066894   D 22545    0 1462501   0    0.00e+00  45090   100.00
 4256    0 1125607   D  4256    0 1521213   0    0.00e+00   8512   100.00
 2433    0 1143845   D  2433    0 1539451   0    0.00e+00   4866   100.00
 2430    0  345369   D  2430    0 4597386   0    0.00e+00   4860   100.00
 8316    0 1058577   D  8316    0 1454184   0    0.00e+00  16632   100.00
 4923    0 1120684   D  4923    0 1516291   0    0.00e+00   9846   100.00
 5904    0 1114779   D  5904    0 1510386   0    0.00e+00  11808   100.00
 2473    0 1112305   D  2473    0 1507913   0    0.00e+00   4946   100.00
 2162    0 1129864   D  2162    0 1525470   0    0.00e+00   4324   100.00
 9438    0 1132027   D  9438    0 1527633   0    0.00e+00  18876   100.00
 6002    0 1100364   D  6002    0 1495971   0    0.00e+00  12004   100.00
 2583    0 1896937   D  2583    0 2708503   0    0.00e+00   5166   100.00
10923    0 1089440   D 10923    0 1485047   0    0.00e+00  21846   100.00
```

Note that 14 supermaximal repeats are reported. Since there are 19 maximal repeats of length $\geq$ 2000 (see above), there are five maximal repeats which are not supermaximal. We can further restrict the set of maximal repeats by only computing branching tandem repeats. We decide to only report tandem repeats of length at least 50.

```
$ vmatch -tandem -l 50 EcoliO157H7
# args=-tandem -l 50 EcoliO157H7
   62    0 2658616   D    62    0 2658678   0    3.96e-25    124   100.00
  282    0 1293997   D   282    0 1294279   0   1.39e-157    564   100.00
  141    0 1294279   D   141    0 1294420   0    1.08e-72    282   100.00
  162    0 2925000   D   162    0 2925162   0    2.46e-85    324   100.00
   57    0 2099948   D    57    0 2100005   0    4.05e-22    114   100.00
   87    0  670299   D    87    0  670386   0    3.52e-40    174   100.00
   92    0  391492   D    92    0  391584   0    3.43e-43    184   100.00
  141    0 4600881   D   141    0 4601022   0    1.08e-72    282   100.00
```

Note that tandem repeats can only be computed on the forward strand. Sometimes the option `-tandem` is too restrictive, and one wants to allow short gaps between the match instances. This can be done by supplying two extra arguments for the option `-l`.

```
$ vmatch -l 50 10 30 EcoliO157H7
# args=-l 50 10 30 EcoliO157H7
   94    0 3679583   D    94    0 3679693   0    2.15e-44    188   100.00
   81    0 3679486   D    81    0 3679596   0    1.44e-36    162   100.00
   72    0  411148   D    72    0  411242   0    3.78e-31    144   100.00
   93    0 5218570   D    93    0 5218681   0    8.58e-44    186   100.00
   86    0 5218473   D    86    0 5218585   0    1.41e-39    172   100.00
   71    0  411544   D    71    0  411638   0    1.51e-30    142   100.00
   70    0  411432   D    70    0  411525   0    6.04e-30    140   100.00
   53    0 3086656   D    53    0 3086737   0    1.04e-19    106   100.00
```

9.9.2 Matching Queries against an Index

In the previous applications we had always compared the index against itself. This section shows applications where query sequences are involved. The corresponding query files containing the query sequences are specified via the option `-q`.

Computing Substring Matches: Let us match a DNA sequence in file `U89959` against `atEST` to find all direct substring matches of length at least 250 with at most 6 differences:

```
$ vmatch -l 250 -e 6 -q U89959 -s 70 atEST
# args=-l 250 -e 6 -q U89959 -s 70 atEST
  270    1265     63   D   269    0  72484   6   1.81e-135    521    97.78
Sbjct: AGTATGGGAAGCCCTGTCTCAGCCTNNGCTTCCAGCNTCTCCTCCTTCCACATCNTTAAAACTCCAACCT         133
                  !           !!!         !                 !
Query: agtatgggaag-cctgtctcagccacggcttccagcctctcctccttccacatcattaaaactccaacct       72553

Sbjct: TGGAAGATTTTAGGAGAATGAGAGCGACACGCTCTGTGCTTCTTTTCCTTATGATCCAGCTCTTCCACGC         203
Query: tggaagattttaggagaatgagagcgacacgctctgtgcttcttttccttatgatccagctcttccacgc       72623

Sbjct: ACAAATGAACTATGAAACATATATAAAGCGCACACATATATTTATGCATATCAAGCTTTTGGTGATTATG         273
Query: acaaatgaactatgaaacatatataaagcgcacacatatatttatgcatatcaagcttttggtgattatg       72693

Sbjct: GTATTGATAGAGTCAAATTAAGCTCGGTGACTATGGTATTAATAAGAGTACTATTTCCTT                   333
Query: gtattgatagagtcaaattaagctcggtgactatggtattaataagagtactatttcctt                 72753
```

So by additionally using the option `-q` we tell `vmatch` to not do a self comparison, but to match all query sequences against `atEST`. The right instance of the match is always in `U89959`, and this file only contains one sequence. Hence the second reported sequence number (in column 6 of the output) is always 0. Moreover, we have used the option `-s 70` to report an optimal alignment between the two match instances. The sequences in the alignment are marked by the keywords `Sbjct` and `Query`. The `Sbjct`-line always shows the the left instance of the match (i.e. the part in the indexed database sequence). The `Query`-line always shows the the right instance of the match (i.e. the part in the query sequence). Alignment columns with insertions, deletions or mismatches are marked by the symbol `!`. The last position of the alignment in every row is shown on the right. If the match instances are longer than the width of a line, it is split into different lines.

The output format is designed to contain all necessary information about the found matches. The kind of information shown on each line can be distinguished according to the first character of the line:

- If the first character of a line is the symbol `#`, then the rest is a comment. It, for example, shows the arguments of the program. The user can also specify a comment line beginning with

  `# smallheading=`

  or with

  `# largeheading=`

  followed by some user defined heading. This can be considered as an annotation, which may be displayed in a graphical user interface visualizing the output of `vmatch`.

- If the first non-white space character is a digit, then this line shows the match record.

- If the first non-white space character is neither the symbol `#` nor a digit, then the line shows the match instances, or an alignment of the match instances. In the latter case the line begins with `Sbjct` or `Query`.

`vmatch` can also produce XML-output format. This is not designed for the human eye, but easy to parse by standard XML-parsers. If we replace the parameter `70` of option `-s` by the keyword `xml` and add the option `-showdesc`, then we obtain the following XML output:

```
$ vmatch -l 250 -e 6 -q U89959 -s xml -showdesc 0 atEST
<?xml version="1.0"?>
<!DOCTYPE Vmatchoutput PUBLIC "-//VMATCH//VMATCH Vmatchoutput/EN" "Vmatchoutput.dtd
<Vmatchoutput>
  <Vmatchglobalparams>
    <Vmatchversion>1.0</Vmatchversion>
    <Vmatchindex>atEST</Vmatchindex>
    <Vmatchquery>U89959</Vmatchquery>
    <Vmatchnumofdbseq>1952</Vmatchnumofdbseq>
    <Vmatchdatabaselength>772375</Vmatchdatabaselength>
    <Vmatchnumofqueryseq>1</Vmatchnumofqueryseq>
    <Vmatchquerylength>106972</Vmatchquerylength>
    <Vmatchalphabet>
      <Vmatchalphabetdomainsize>32</Vmatchalphabetdomainsize>
      <Vmatchalphabetmapsize>5</Vmatchalphabetmapsize>
      <Vmatchalphabetmappedwildcards>22</Vmatchalphabetmappedwildcards>
      <Vmatchalphabetundefsymbol>253</Vmatchalphabetundefsymbol>
      <Vmatchalphabetdomain>aAcCgGtTuUnsywrkvbdhmNSYWRKVBDHM</Vmatchalphabetdomain>
      <Vmatchalphabetverbosechar>acgtn</Vmatchalphabetverbosechar>
    </Vmatchalphabet>
  </Vmatchglobalparams>
  <Vmatchiterationmatches>
    <Match>
      <Vmatchmatchidnumber>0</Vmatchmatchidnumber>
      <Vmatchlength1>270</Vmatchlength1>
      <Vmatchseqnum1>1265</Vmatchseqnum1>
      <Vmatchdescription1>gi|3449674|gb|AI099935.1|AI099935</Vmatchdescription1>
      <Vmatchrelpos1>63</Vmatchrelpos1>
      <Vmatchflag>D</Vmatchflag>
```

```
      <Vmatchlength2>269</Vmatchlength2>
      <Vmatchseqnum2>0</Vmatchseqnum2>
      <Vmatchdescription2>Arabidopsis</Vmatchdescription2>
      <Vmatchrelpos1>63</Vmatchrelpos1>
      <Vmatchrelpos2>72484</Vmatchrelpos2>
      <Vmatchdistance>6</Vmatchdistance>
      <Vmatchevalue>1.81e-135</Vmatchevalue>
      <Vmatchscore>521</Vmatchscore>
      <Vmatchidentity>97.78</Vmatchidentity>
      <DNA_eops>
        <DNA_eop_type>match</DNA_eop_type>
        <DNA_eop_length>11</DNA_eop_length>
        <DNA_eop_type>deletion</DNA_eop_type>
        <DNA_eop_length>1</DNA_eop_length>
        <DNA_eop_type>match</DNA_eop_type>
        <DNA_eop_length>12</DNA_eop_length>
        <DNA_eop_type>mismatch</DNA_eop_type>
        <DNA_eop_length>3</DNA_eop_length>
        <DNA_eop_type>match</DNA_eop_type>
        <DNA_eop_length>9</DNA_eop_length>
        <DNA_eop_type>mismatch</DNA_eop_type>
        <DNA_eop_length>1</DNA_eop_length>
        <DNA_eop_type>match</DNA_eop_type>
        <DNA_eop_length>17</DNA_eop_length>
        <DNA_eop_type>mismatch</DNA_eop_type>
        <DNA_eop_length>1</DNA_eop_length>
        <DNA_eop_type>match</DNA_eop_type>
        <DNA_eop_length>215</DNA_eop_length>
      </DNA_eops>
    </Match>
  </Vmatchiterationmatches>
</Vmatchoutput>
```

The XML-output starts with some header giving the kind of output a name, in this case it is called Vmatchoutput. The output of a single vmatch run is shown between <Vmatchoutput> and </Vmatchoutput>. In the first part of the output some global parameters are defined, like the name of the index and the query (if any). Their sizes and the numbers of sequences they contain are also given. The alphabet of the given sequences is followed a list of matches. For each match, its ID-number is given, and all important parameters, all tagged such that they are easily identified by an XML-parser to process them by other tools. Since the option -showdesc is given, the sequence number and the sequence description is reported. The match is completed by an alignment in form of a sequence of edit operations. There are four kinds of edit operations, namely match, deletion, insertion, and mismatch. For each of these operations a positive number is specified which gives the number of character involved in the operation, i.e. the number of characters matched, or deleted, etc.

Computing Maximal Unique Matches: In some applications it is helpful to restrict the set of maximal substring matches to those which only occur exactly once in the database and once in the query. That is, one wants to restrict to maximal unique matches. Using the option -mum, we can compute these. In the following example, we only output the best 10 maximal unique matches (out of 28828).

```
$ vmatch -mum -l 30 -q EcoliK12 -best 10 EcoliO157H7
# args=-mum -l 30 -q EcoliK12 -best 10 /vol/vstree/src/vstree/src/doc/EcoliO157H7
 2632    0  198385   D  2632    0  195040   0    0.00e+00   5264   100.00
```

```
 2567    0 4189568   D  2567    0 3442567   0    0.00e+00   5134   100.00
 2153    0 4184202   D  2153    0 3437201   0    0.00e+00   4306   100.00
 1982    0  482323   D  1982    0  419564   0    0.00e+00   3964   100.00
 1973    0 1234212   D  1973    0 1014741   0    0.00e+00   3946   100.00
 1973    0 4725062   D  1973    0 3917861   0    0.00e+00   3946   100.00
 1845    0 4186356   D  1845    0 3439355   0    0.00e+00   3690   100.00
 1644    0 4171484   D  1644    0 3424482   0    0.00e+00   3288   100.00
 1616    0 4154547   D  1616    0 3407602   0    0.00e+00   3232   100.00
 1552    0 4195622   D  1552    0 3448621   0    0.00e+00   3104   100.00
```

There are 32236 maximal substring matches of length 30 or longer between the two Ecoli genomes. The number of MUM-candidates of this length is 28828, and the number of maximal maximal unique matches is 28409.

Computing Complete Matches: Now suppose, we have a set of strings in a file `ORFs`:

```
$ cat ORFs
>orf1
CTCTTCCAGT
>orf1
GTCTAGGTTT
```

We want to match each of these strings completely against the index. We can use the option `-complete`. We want to allow direct matches as well as palindromic matches. So we combine option `-d` and `-p`. Option `-s` additionally reports the matching sequences. For palindromic matches the reverse complement of the matching sequence is shown:

```
$ vmatch -complete -d -p -q ORFs -s -showdesc 10 atEST
# args=-complete -d -p -q ORFs -s -showdesc 10 atEST
   10   gi|2764223    272   D    10   orf1  0   0    4.14e-01     20   100.00
Sbjct: CTCTTCCAGT                                                       282
Query: CTCTTCCAGT                                                        10


   10   gi|4714046     26   D    10   orf1  0   0    4.14e-01     20   100.00
Sbjct: GTCTAGGTTT                                                        36
Query: GTCTAGGTTT                                                        10


   10   gi|4714003     10   D    10   orf1  0   0    4.14e-01     20   100.00
Sbjct: GTCTAGGTTT                                                        20
Query: GTCTAGGTTT                                                        10


   10   gi|3449668    225   P    10   orf1  0   0    4.14e-01     20   100.00
Sbjct: AAACCTAGAC                                                       235
Query: AAACCTAGAC                                                        10
```

Consider another example where we have two patterns in a file called `Patterns`:

```
$ cat Patterns
>pattern 1
```

```
AGCTCTCTAGAGATAGA
>pattern 2
ATCGCCTATAAGAGACTCTCG
```

Both patterns do not occur in the *Ecoli O157:H7* genome, as can easily be verified:

```
$ vmatch -complete -d -q Patterns EcoliO157H7
# args=-complete -d -q Patterns EcoliO157H7
```

We want to know, if the patterns occur with up to 25% differences. The percentage is relative to the length of the pattern. For the first pattern of length 18, 25% differences corresponds to an absolute difference threshold of 4. For the second pattern of length 21, 25% differences correspond an absolute difference threshold of 5. We can use the percentage search mode via option `-e 25p`, this time only counting the number of matches:

```
$ vmatch -i -complete -e 25p -q Patterns EcoliO157H7
# args=-i -complete -e 25p -q Patterns EcoliO157H7
# all 145
# 14 2
# 15 9
# 16 18
# 17 23
# 18 35
# 19 22
# 20 20
# 21 9
# 22 7
```

Thus we obtain 145. Far more than we want to see. Maybe the pattern occurs with less than 4 respectively 5 differences in the genome. We can use the best search mode, to find the best matches, i.e. those with the smallest number of differences:

```
$ vmatch -complete -e 25b -q Patterns -s EcoliO157H7
# args=-complete -e 25b -q Patterns -s EcoliO157H7
   17    0 1150435   D    18    0  0   3    3.22e+01     26    83.33
Sbjct: AGCTCGC-CGAGATAGGA                                     1150452
            ! !!
Query: AGCTCTCTAGAGATAGGA                                          18


   20    0 3435749   D    21    1  0   4    2.58e+01     29    80.95
Sbjct: ATCGCCAAT-AGAG-CTGCTCG                                 3435769
             !  !    !  !
Query: ATCGCCTATAAGAGACT-CTCG                                      21
```

That is, for the first pattern we have found a match with three differences, and for the second pattern a match with four differences.

9.9.3 Matching a DNA sequence against a Protein Index

Using the option `-dnavsprot`, we can match a DNA query sequence against an index built from a protein sequence. The DNA sequence is translated in all six reading frames. The resulting sequence of aminoacids is compared to the protein sequence. The position and length of the match referring to the query are shown with respect to the original DNA sequence. Hence a match of length l in the indexed protein database sequence is reported as a match of length $3l$ on the codon level. Consider a file `Codonseq.fna`:

```
$ cat Codonseq.fna
>
TACTTCAACAACCGATTACGACAAACAAACATGGTAGTATTCAACAACTAC
TACTTACACTCAAAACGATTACGAACAAACATGTTCATATTAAACAACTAC
```

We match this file against the index `Proteinseq`, which was constructed earlier, using the symbol mapping `TransProt11`:

```
$ vmatch -q Codonseq.fna -dnavsprot 1 -e 1 -l 19 -s Proteinseq
# args=-q Codonseq.fna -dnavsprot 1 -e 1 -l 19 -s Proteinseq
28    1  15   F 84    0  0   0    2.71e-84 112   100.00
Sbjct: YFNNIVKRLRQTNMVVFNNYYLHSKRLR                                         43
Query: YFNNIVKRLRQTNMVVFNNYYLHSKRLR                                         28


27    0  16   F 84    0  0   1    9.03e-81 108    98.81
Sbjct: YFNNIVKR-RQTNMFILNNYYFHSKRIR                                         43
               !     ===    =    =
Query: YFNNIVKRLRQTNMVVFNNYYLHSKRLR                                         28
```

Note that the symbol mapping `TransProt11` is inherited from `Proteinseq`, when comparing the translated codons of the six reading frames of `Codonseq.fna` with the protein sequence. Also note that the fourth column of the position line shows the symbol `F`. This means that the right match instance (in the query sequence) refers to one of the three reading frames on the forward strand. In case the right match instance refers to the one of the three reading frames on the reverse strand, the symbol `G` would be shown.

9.9.4 Matching a DNA sequence on the Protein Level

To match a DNA sequence on the Protein level, we construct a six frame translation index, using the program `mkdna6idx`. We do this for the file `Codonseq.fna` of the previous section.

```
$ mkdna6idx -db Codonseq.fna -indexname Codonseq -tis -ois -v
reading file "Codonseq.fna"
total length of sequences: 102
create file "Codonseq.tis"
create file "Codonseq.ois"
create file "Codonseq.des"
```

```
create file "Codonseq.sds"
create file "Codonseq.prj"
create file "Codonseq.al1"
maximal value for argument of option -pl is 1, recommended value is 1
total length of sequences: 205 (including 5 separators)
alphabet "LVIFKREDAGSTNQYWPHMCXUBZ*-" (size 26) mapped to "LVIFKREDAGSTNQYWPHMCX" (size 21)
create file "Codonseq.6fr.tis"
create file "Codonseq.6fr.ois"
create file "Codonseq.6fr.des"
create file "Codonseq.6fr.sds"
create file "Codonseq.6fr.lcp"
initializing data structures
sorting suffixes according to prefix of length 1
sorting all buckets
create file "Codonseq.6fr.llv"
create file "Codonseq.6fr.suf"
create file "Codonseq.6fr.bwt"
create file "Codonseq.6fr.prj"
create file "Codonseq.6fr.al1"
# overall space peak: main=0.13 MB,  secondary=0.00 MB
```

The generated index has a name ending with suffix `.6fr`. The additional plain text index named `Codonseq` is used for transforming and showing the matches. We want to compute exact matches of length at least 12 on the protein level:

```
$ vmatch -l 12 -s Codonseq.6fr
# args=-l 12 -s Codonseq.6fr
12    0  20   F 12    0  71   0    1.15e-12  24   100.00
Sbjct: ACAAACAAACAT                                                          32
         !
Sbjct: ACGAACAAACAT                                                          83


12    0  70   I 12    0  19   0    1.15e-12  24   100.00
Sbjct: TGTTTGTTCGTA                                                          82
               !  !
Sbjct: TGTTTGTTTGTC                                                          31
```

As usual, the symbol `!` shows alignment columns with mismatches. However, the mismatching bases still allow a match on the corresponding translated codon. Note that the fourth column of the position line shows the symbols `F` and `I`. These symbols describe if the match instances are on the three reading frames of the forward strand or of the three reading frames on the reverse strand. Since all combinations are possible, there are two additional symbols `G` and `H` besides `F` and `I`. The following table shows which symbol stands for which combination:

symbol	left match instance	right match instance
`F`	forward strand reading frame	forward strand reading frame
`G`	forward strand reading frame	reverse strand reading frame
`H`	reverse strand reading frame	forward strand reading frame
`I`	reverse strand reading frame	reverse strand reading frame

9.9.5 Computing Regions not containing a Match

There are applications, where it is not important to know where the matches are. Instead, one is interested in knowing those regions of the database sequences or of the query sequences where *no* match occurs. For these kinds of applications `vmatch` provides the options `-dbnomatch` and `-qnomatch`. Consider, for example, a comparison of the index against itself. The corresponding matches can be called repeats, since they occur more than once in the same sequence, or sets of sequences. Those parts of the sequence not covered by a repeat can then be considered unique substrings. To compute these, we can use the option `-dbnomatch` which requires an additional argument specifying the least length of the unique substrings to be reported. For example, the following program call reports all substrings of length at least 700 which do not contain a direct repeat of length 25 with at most 1 difference:

```
$ vmatch -dbnomatch 700 -l 25 -e 1 atEST
# args=-dbnomatch 700 -l 25 -e 1 atEST
>10 0 815
>26 0 1019
>32 0 1039
>77 191 742
>304 0 725
```

Each line beginning with the symbol > reports the sequence number, the relative start position, and the length of a unique substring (i.e. a substring not covered by a repeat instance) in the sequence under consideration. As usual, the output format can be modified by the options `-f`, `-showdesc`, `-absolute`, and `-s`. For example, if we additionally use the option `-s`, then also the sequence content of the unique substrings is reported. This gives an output file in FASTA-format. Additionally, the >-line shows the character distribution of the sequence reported.

The option `-qnomatch` can be used when comparing a set of query sequences against the database sequences. For example, the following program call reports all regions of length 10000 in the genome of *Ecoli K12* which do not contain any exact match to a substring of length 18 or longer in the *Ecoli O157:H7* genome.

```
$ vmatch -qnomatch 10000 -q EcoliK12 -l 18 EcoliO157H7
# args=-qnomatch 10000 -q EcoliK12 -l 18 EcoliO157H7
>0 1385319 10800
>0 1398241 10711
>0 1570168 20414
>0 1599821 12375
>0 2755646 11820
```

Each line beginning with the symbol > reports the starting position and the length of a substring in the query sequences which is not common to both genomes under consideration. If we additionally use the option `-s`, then also the sequences in the query which are not common with the database sequences are reported.

Alternatively, we can output the substrings in the *Ecoli O157:H7* genome not occurring in *Ecoli K12*. These could be good candidates to explain the pathogenicity of *Ecoli O157:H7*.

```
$ vmatch -dbnomatch 10000 -q EcoliK12 -l 18 EcoliO157H7
# args=-dbnomatch 10000 -q EcoliK12 -l 18 EcoliO157H7
>0 664171 11617
>0 1087635 12158
>0 1105699 10152
>0 1117238 14153
>0 1278595 13158
>0 1434907 13857
>0 1483242 12158
>0 1501306 10152
>0 1512845 14152
>0 1652802 11515
>0 1718335 11241
>0 1956644 11322
>0 2030357 12870
>0 2111045 12614
>0 2934852 12334
>0 3206647 12308
>0 3729821 15836
>0 4603137 15160
>0 4618316 13631
>0 5319491 10210
>0 5339473 11560
```

9.9.6 Masking Matches

The options `-dbmaskmatch` and `-qmaskmatch` are very similar to the *nomatch*-options used in the previous section. While the *nomatch*-options output those parts of the sequences *not* covered by a match, the *maskmatch*-options allows to mask the matches in the database sequences or in the query sequences. For example, the following program call masks the left instance of substring matches of length ≥ 20 when comparing the index `swissp.sp.gz` against itself. `X` is used as a masking character.

```
$ vmatch -l 20 -s 90 -dbmaskmatch X swissp.sp.gz
>110K_PLAKN 110 KD ANTIGEN (PK110) (FRAGMENT).
FNSNMLRGSVCEEDVSLMTSIDNMIEEIDFYEKEIYKGSHSGGVIKGMDYDLEDDENDEDEMTEQMVEEVADHITQDMIDEVAHHVLDNI
THDMAHMEEIVHGLSGDVTQIKEIVQKVNVAVEKVKHIVETEETQKTVEPEQIEETQNXXXXXXXXXXXXXXXXXXXXXXXXXXXXXQIE
ETQKTVEPEQTEEAXXXXXXXXXXXXXXXXXXXXXXXXXXXXXXXXXXXXXXXXXXXXXXXXXXXXXXXXXXXXXXXXXXXXXXXXXXXX
XXXXXXXXXXXXXXXPTQETQNTVEP
```

Of course, the masking options can also be used when matching a query against an index. Then there are two choices. One can either mask the database sequences or the query sequences. For example, the following application masks both instances of the substring matches of length 70 or longer between *Ecoli O157:H7* and *Ecoli K12*. That is, the matches in the database sequence represented by the index `EcoliO157H7` are masked by converting the original lower case characters to upper case characters.

```
$ vmatch -l 70 -q EcoliK12 -s 70 -dbmaskmatch tolower EcoliO157H7
>Ecoli O157:H7 Complete Genome
AGCTTTTCATTCTGACTGCAACGGGCAATATGTCTCTGTGTGGATTAAAAAAAGAGTCTCTGACagcagc
ttctgaactggttacctgccgtgagtaaattaaaattttattgacttaggtcactaaatactttaaccaa
tataggcatagcgcacagacagataaaaattacagagtacacaacatccatgaaacgcattagcaccacc
attaccaccaccatcaccaCCACCATCACCATTaccattaccacaggtaacggtgcgggctgacgcgtac
aggaaacacagaaaaaagcccgcacctgacagtgcgggctttttttttcgaccaaaggtaacgaggtaaca
accatgcgagtgttgaagttcggcggtacatcagtggcaaatgcagaacgttttctgcgGgttgccgata
ttctggaaagcaatgccaggcaggggcaggtggccaccgtcctctctgcccccgccaaaatcaccaacca
cctggtggcgatgattgaaaaaaccattagcggccaggatgctttacccaatatcagcgatgccgaacgt
atttttgccgaacttCTGACGGGACTCGCCGCCGCCCAGCCGGGATTCCCGCTGGCGCAATTGAAAACTT
```

It is easily visible that there are several matches in the first few hundred bases of `EcoliO157H7`.

To mask the substrings of the query sequence *Ecoli K12* covered by a match, we replace option `-dbmaskmatch` by `-qmaskmatch`. We also use the flag `tolower`, since the query sequence consists of upper case characters.

```
$ vmatch -l 70 -q EcoliK12 -s 70 -qmaskmatch tolower EcoliO157H7
>gb|U00096|U00096 Escherichia coli K-12 MG1655 complete genome
AGCTTTTCATTCTGACTGCAACGGGCAATATGTCTCTGTGTGGATTAAAAAAAGAGTGTCTGATagcagc
ttctgaactggttacctgccgtgagtaaattaaaattttattgacttaggtcactaaatactttaaccaa
tataggcatagcgcacagacagataaaaattacagagtacacaacatccatgaaacgcattagcaccacc
attaccaccaccatcaccattaccacaggtaacggtgcgggctgacgcgtacaggaaacacagaaaaaag
cccgcacctgacagtgcgggcttttttttttcgaccaaaggtaacgaggtaacaaccatgcgagtgttgaa
gttcggcggtacatcagtggcaaatgcagaacgttttctgcgTgttgccgatattctggaaagcaatgcc
aggcaggggcaggtggccaccgtcctctctgcccccgccaaaatcaccaaccacctggtggcgatgattg
aaaaaaccattagcggccaggatgctttacccaatatcagcgatgccgaacgtatttttgccgaacttTT
GACGGGACTCGCCGCCGCCCAGCCGGGGTTCCCGCTGGCGCAATTGAAAACTTTCGTCGATCAGGAATTT
```

9.9.7 Clustering Indexed Sequences

Clustering a set of sequences according to matches occurring in the sequences is an important task. It is done, for example, when clustering ESTs or protein sequences. The following command computes clusters of all database sequences in `atEST`, based on all matches of length at least 200 with at most 10 differences. The matches must cover at least 30% of the smaller of the two matching sequences and 50% of the larger of the two sequences.

```
$ vmatch -dbcluster 30 50 -p -d -l 200 -e 10 atEST
# args=-dbcluster 30 50 -p -d -l 200 -e 10 atEST
# 152 clusters
# 389 elements out of 1952 (19.93%) are in clusters
# 1563 elements out of 1952 (80.07%) are singlets
# 124 clusters of size 2
# 18 clusters of size 3
# 4 clusters of size 4
# 2 clusters of size 5
# 1 cluster of size 6
```

```
# 1 cluster of size 7
# 1 cluster of size 8
# 1 cluster of size 40
0:  1646 1862
1:  3 39 6
2:  1764 1896 1745 1767 1882
```

We only show the first 3 clusters of size at least two. For example, cluster 2 contains the sequences with numbers 1764, 1896, 1745, 1767, 1882.

Instead of the sequence numbers, we can output the descriptions, using the option `-showdesc 0`.

```
$ vmatch -dbcluster 30 50 -p -d -l 200 -e 10 -showdesc 0 atEST
# args=-dbcluster 30 50 -p -d -l 200 -e 10 -showdesc 0 atEST
# 152 clusters
# 389 elements out of 1952 (19.93%) are in clusters
# 1563 elements out of 1952 (80.07%) are singlets
# 124 clusters of size 2
# 18 clusters of size 3
# 4 clusters of size 4
# 2 clusters of size 5
# 1 cluster of size 6
# 1 cluster of size 7
# 1 cluster of size 8
# 1 cluster of size 40
0:
  gi|2764304|gb|R65326.1|R65326
  gi|2764088|gb|R30410.1|R30410
1:
  gi|4714047|dbj|C99930.1|C99930
  gi|4714011|dbj|C99887.1|C99887
  gi|4714044|dbj|C99927.1|C99927
2:
  gi|2764186|gb|R64887.1|R64887
  gi|2764054|gb|R30268.1|R30268
```

To output more information about the clustered sequences we supply the option `-dbcluster` with a third argument `CLatEST` and add the option `-s`.

```
$ vmatch -dbcluster 30 50 CLatEST -s -p -d -l 200 -e 10 atEST
# args=-dbcluster 30 50 CLatEST -s -p -d -l 200 -e 10 atEST
# 152 clusters
# 389 elements out of 1952 (19.93%) are in clusters
# 1563 elements out of 1952 (80.07%) are singlets
# 124 clusters of size 2
# 18 clusters of size 3
# 4 clusters of size 4
# 2 clusters of size 5
# 1 cluster of size 6
```

```
# 1 cluster of size 7
# 1 cluster of size 8
# 1 cluster of size 40
```

This generates files `CLatEST.`s`.`i`.match` and `CLatEST.`s`.`i`.fna` containing the matches and the clustered sequences for cluster i. s is the number of sequences in the cluster. For example,

```
$ cat CLatEST.5.2.match
  228    1882     70   D   229    1896     73   8   4.36e-106
  218    1767     90   D   219    1896     83   7   1.85e-102
  218    1745     90   D   219    1896     83   7   1.85e-102
  336    1764     74   D   336    1882     63   7   1.39e-171
  330    1764     74   D   334    1767     73  10   2.86e-163
  310    1745     73   D   308    1764     74   9   1.92e-151
...
```

shows the matches responsible for the cluster 2 of size 5. Furthermore,

```
$ cat CLatEST.5.2.fna
>gi|2764186|gb|R64887.1|R64887 13391 Lambda-PRL2 Arabidopsis thaliana cDNA
ACAAGAACTCAAACACTTCATAACTAAAACATCCTTTAANGCCTTTNNAAAAACTCAATC
ATGTCAAGCAACTNCGGAAGCTGCGACTGTCCTGACAAGACCCAGTGCGTAAAGAAGGGA
ACCAGCTACACCTTCGACATCGTCGAGACTCAGGAGAGCTACAAGGAGGCCATGATCATG
GACGTTGGTGCCGAGGAGAACAACGCAAATTGCAAGTNCAAGTNCGGCTCCTCTTGCAGC
TGCGTCAACTGCACTTGCTGCCCCAACTAATGANGCTTCTTTAATCAAAATGTAATATGA
ATAAAAGTTGATGTGGGCTCATCTATTGAGCTCATGTNTCTNTTATTACTACTCTCTAGT
ATGGTGTGATGTAATGGGTTATGACCCTTCTTTCCCTTCCCTATAAAACTNAAGGANCTT
GCAAGGTT
>gi|2764054|gb|R30268.1|R30268 12873 Lambda-PRL2 Arabidopsis thaliana cDNA
CTCAAACACTTCATAACTAAACATCCTTTAAAGCCTTTTCAAAAACTCAATCATGTCAAG
CAACTGCGGAAGCTGCGACTGTCCTGACAAGACCCAGTGCGTAAAGAAGGGAACCAGCTA
CACCTTCGACATCGTCGAGACTCAGGAGAGCTACAAGGAGGCCATGATCATGGACGTTGG
TGCCGAGGAGAACAACGCAAATTGCAAGTNCAAGTNCGGCTCCTCTTGCAGCTGCGTNAA
CTGCACTTGCTGCCCCCAACTAATGANGCTTCTTTAATCAAAATGTAATATGNNTAAAAG
TTTNATGTNGGGGCTCATCCTATTTGAGNCTCATGGTTTCTCTTTATTACTACCNCTCTN
GTAATGGGGGTGATGTAATGGGGTTNTTACCCCNTCTTCCCNTNCCCNTTAAAAACT
...
```

shows the sequences of this cluster.

9.9.8 Computing a non-redundant set of sequences

A colleague recently sent a list of 8124 sequences for different virus strains of the Mouth and Foot Disease. The goal was to produce a multiple alignment of the sequences. Given the GI-numbers of the sequences, it was easy to download the sequences from Genbank resulting in 126 files. The lengths of the sequences is in the range between 7733 and 8280. The total length of all 8124 sequences was . A simple call to `mkvtree` with all 126 files given as arguments to option `-db`, creates an index for all sequences. The name of the index is `mfdindex`. Since the construction of the index `mfdindex` takes longer than expected, there seemed to be long common substrings in the sequences. This can easily be verified by clustering the matches:

```
vmatch -l 7000 -seedlength 1000 -exdrop 2 -dbcluster 100 100 mfdindex
```

This means that the sequences are clustered based on matches of length 7000 or longer with seeds of length 1000 or longer, allowing an Xdrop threshold of 2. The parameters `100 100` to option `-dbcluster` specify that two sequences are clustered only if a match covers the sequence completely. That is, the sequences in each cluster are pairwise identical.

We obtain 110 clusters and 1 one singlet. That is 8213 sequences are in cluster of size at least two, and one sequence is singleton. So to compute an optimal alignment, it makes sense to only continue with the singlet and exactly one sequence from each cluster. These 111 sequences can be extracted by adding the option `-nonredundant` to the previous call of `vmatch`. The argument of this option is the name of the file to store the nonredundant sequences:

```
vmatch -l 7000 -seedlength 1000 -exdrop 2 -dbcluster 100 100 -v -nonredundant nrset mfdindex
```

Having computed the non-redundant set, we can, for example, continue with computing a multiple alignment using the software MGA [5].

9.9.9 Chaining Matches

We only give two examples of using the option `-pp chain`. We compare the *E.coli* genomes *Ecoli K12* and *Ecoli O157:H7* and compute all 659 matches of length 500 or longer between these sequences. The first example shows how to compute a global chain. The optimal global chain contains 597 matches (i.e. 91% of all matches), of which we only show the first and the last seven.

```
$ vmatch -l 500 -q EcoliK12 -pp chain global EcoliO157H7
# args=-l 500 -q EcoliK12 -pp chain global EcoliO157H7
# chain 0: length 596 score 835932
  512    0   13736   D   512    0   13719   0    0.00e+00   1024   100.00
  645    0   59313   D   645    0   54337   0    0.00e+00   1290   100.00
 1501    0   59959   D  1501    0   54983   0    0.00e+00   3002   100.00
  895    0   61917   D   895    0   56941   0    0.00e+00   1790   100.00
  515    0   65876   D   515    0   60943   0    0.00e+00   1030   100.00
  502    0   67856   D   502    0   62923   0    0.00e+00   1004   100.00
  671    0 5448270   D   671    0 4615599   0    0.00e+00   1342   100.00
  915    0 5449694   D   915    0 4617023   0    0.00e+00   1830   100.00
  577    0 5450982   D   577    0 4618311   0    0.00e+00   1154   100.00
  597    0 5455239   D   597    0 4623611   0    0.00e+00   1194   100.00
  553    0 5457015   D   553    0 4625387   0    0.00e+00   1106   100.00
  871    0 5457972   D   871    0 4626344   0    0.00e+00   1742   100.00
  828    0 5469153   D   828    0 4637525   0    0.00e+00   1656   100.00
```

To find spots of high local similarity, we instead perform local chaining. We compute the local chains with the best and the second best scores: We obtain two chains. The highest scoring chain has score 23118 and is of length 10. The second highest scoring chain has score 12654 and is of length 7:

```
$ vmatch -l 500 -q EcoliK12 -pp chain local 2b EcoliO157H7
# args=-l 500 -q EcoliK12 -pp chain local 2b EcoliO157H7
# chain 0: length 7 score 12654
  749    0  193444   D   749    0  189957  0    0.00e+00   1498   100.00
  592    0  194194   D   592    0  190707  0    0.00e+00   1184   100.00
 1210    0  194787   D  1210    0  191300  0    0.00e+00   2420   100.00
 1065    0  196408   D  1065    0  193063  0    0.00e+00   2130   100.00
  505    0  197510   D   505    0  194165  0    0.00e+00   1010   100.00
 2632    0  198385   D  2632    0  195040  0    0.00e+00   5264   100.00
  589    0  201135   D   589    0  197790  0    0.00e+00   1178   100.00
# chain 1: length 10 score 23118
 2153    0 4184202   D  2153    0 3437201  0    0.00e+00   4306   100.00
 1845    0 4186356   D  1845    0 3439355  0    0.00e+00   3690   100.00
  575    0 4188373   D   575    0 3441372  0    0.00e+00   1150   100.00
  537    0 4188949   D   537    0 3441948  0    0.00e+00   1074   100.00
 2567    0 4189568   D  2567    0 3442567  0    0.00e+00   5134   100.00
  798    0 4192136   D   798    0 3445135  0    0.00e+00   1596   100.00
  833    0 4192935   D   833    0 3445934  0    0.00e+00   1666   100.00
  952    0 4194011   D   952    0 3447010  0    0.00e+00   1904   100.00
 1552    0 4195622   D  1552    0 3448621  0    0.00e+00   3104   100.00
  917    0 4197175   D   917    0 3450174  0    0.00e+00   1834   100.00
```

For examples showing the effect of the different chaining options, see the corresponding manual for the program *chain2dim*.

9.9.10 Clustering Matches

Suppose we have constructed an index for yeast chromosome III:

```
$ mkvtree -dna -db ychrIII.fna -v -tis -ois -bwt -suf -lcp
reading file "ychrIII.fna"
total length of sequences: 315339
create file "ychrIII.fna.tis"
create file "ychrIII.fna.ois"
create file "ychrIII.fna.des"
create file "ychrIII.fna.sds"
create file "ychrIII.fna.lcp"
initializing data structures
sorting suffixes
create file "ychrIII.fna.llv"
create file "ychrIII.fna.suf"
create file "ychrIII.fna.bwt"
create file "ychrIII.fna.prj"
create file "ychrIII.fna.al1"
overall space peak: main=2.82 MB (9.38 bytes/symbol), secondary=0.31 MB
```

Now compute all repeats of length ≥ 100 in yeast chromosome III, and cluster these matches by similarity, using an error rate of 35. The cluster files produced have the prefix `clout`:

```
$ vmatch -l 100 -pp matchcluster erate 35 outprefix clout ychrIII.fna
# args=-l 100 -pp matchcluster erate 35 outprefix clout ychrIII.fna
# cluster 16 matches
# create cluster 0 of size 2
# create cluster 1 of size 3
# create cluster 2 of size 2
```

Three files where generated by this program call:

```
$ ls -l clout.*
-rw-r-----    1 kurtz    gistaff       285 2005-02-21 16:41 clout.2.0.match
-rw-r-----    1 kurtz    gistaff       289 2005-02-21 16:41 clout.2.2.match
-rw-r-----    1 kurtz    gistaff       491 2005-02-21 16:41 clout.3.1.match
```

Consider the last file `clout.3.1.match` representing cluster 1 with three matches:

```
$ cat clout.3.1.match
# args=-l 100 ychrIII.fna
# id 7
  280      0  83954   D   280      0  84469   0   7.41e-159    560   100.00
# id 8
  239      0  83954   D   239      0  90099   0   3.58e-134    478   100.00
# id 12
  286      0  84422   D   286      0  90052   0   1.81e-162    572   100.00
# linked 8 and 12 with edit distance 47 (error rate 19.67%)
# linked 7 and 12 with edit distance 88 (error rate 31.43%)
# linked 7 and 8 with edit distance 41 (error rate 17.15%)
```

Cluster 1 thus contains the matches with identification numbers 7, 8, and 12. For example, match 7 and 12 achieve a distance of 88, which corresponds to an error rate of 31.43%, well below the maximum error rate of 35%. Note that the generated files are in a format that they can be read by `vmatchselect`.

Instead of clustering by similarity, we can cluster by gap size, allowing gaps of size up to 1000.

```
$ vmatch -l 100 -pp matchcluster gapsize 1000 outprefix clout ychrIII.fna
# args=-l 100 -pp matchcluster gapsize 1000 outprefix clout ychrIII.fna
# cluster 16 matches
# create cluster 0 of size 9
# create cluster 1 of size 4
```

Consider the file `clout.4.1.match` representing cluster 1 with four matches: It contains the matches with identification numbers 14, 7, 8, and 12. For example, match 8 and 7 have a gap of $276 = 84469 - 83954 - 239$ which is well below the maximum gap size of 1000.

```
$ cat clout.4.1.match
# args=-l 100 ychrIII.fna
# id 14
  126      0  83680   D   126      0  84422   0    3.86e-66    252   100.00
# id 7
  280      0  83954   D   280      0  84469   0   7.41e-159    560   100.00
# id 8
  239      0  83954   D   239      0  90099   0   3.58e-134    478   100.00
# id 12
  286      0  84422   D   286      0  90052   0   1.81e-162    572   100.00
# linked 8 and 7 with gapsize 276
# linked 8 and 14 with gapsize 229
# linked 8 and 12 with gapsize 229
# linked 14 and 7 with gapsize 663
# linked 14 and 12 with gapsize 616
# linked 14 and 8 with gapsize 148
# linked 14 and 7 with gapsize 148
```

In a third run we cluster the matches by overlap, allowing overlaps of minimum 10%.

```
$ vmatch -l 100 -pp matchcluster overlap 10 outprefix clout ychrIII.fna
# args=-l 100 -pp matchcluster overlap 10 outprefix clout ychrIII.fna
# cluster 16 matches
# create cluster 0 of size 3
# create cluster 1 of size 5
# create cluster 2 of size 3
```

Consider the file `clout.3.2.match` representing cluster 2 with three matches: It contains the matches with identification numbers 0, 6, and 1. For example, match 0 and 6, overlap by $82 = 199591 + 203 - 199712$ positions, which is 40.39% of the length 203 of the longer match.

```
$ cat clout.3.2.match
# args=-l 100 ychrIII.fna
# id 0
  203      0 199591   D   203      0 293111   0   1.69e-112    406   100.00
# id 6
  195      0  13811   D   195      0 199712   0   1.11e-107    390   100.00
# id 1
  120      0  13690   D   120      0 293111   0    1.58e-62    240   100.00
# linked 0 and 1 with overlap percentage 100.00
# linked 0 and 6 with overlap percentage 40.39
```

9.9.11 Selection Functions

The concept of selection functions (bundles) is extensively explained in Appendix E. The following function bundle (only consisting of the function `selectmatch`), defined in a file `sel392.c`, accepts matches of length at most 392.

```
#include <string.h>
#include "select.h"

int selectmatch(Alphabet *alpha,
                Multiseq *virtualmultiseq,
                Multiseq *querymultiseq,
                StoreMatch *storematch)
{
  if(storematch->Storelength1 <= 392)
  {
    return 1;  /* accept */
  } else
  {
    return 0;  /* reject */
  }
}
```

To compile the appropriate shared object file under Linux or a Compaq-Alpha/True64 system, we use the `gcc` compiler with the following options:

```
$ gcc -Wall -Werror -O3 -shared sel392.c -o sel392.so
```

On a SUN-Sparc/Solaris computer, the option `-shared` is replaced by the option `-G`. Note that in case you are using the 64-bit version of the program you have to add the compiler option `-m64`. For statically linked executables of `vmatch` and `vmatchselect` selection functions only work under very restrictive circumstances: The machine which runs `vmatch` or `vmatchselect` must have the same versions of the libraries as the machine on which the statically linked binaries were compiled.

Now we can select the matches using the shared object `sel392.so`. This rejects the two matches of length 517 and 402, see Section 9.9.1, above.

```
$ vmatch -l 350 -selfun sel392.so atEST
  388     79    236   D   388    1495    180   0   4.22e-223    776   100.00
  392    229      0   D   392     270      0   0   1.65e-225    784   100.00
  369    736    141   D   369    1151     85   0   1.16e-211    738   100.00
  369    902     54   D   369    1151     87   0   1.16e-211    738   100.00
  367    736    143   D   367     902     54   0   1.86e-210    734   100.00
```

In the usual case you want to call the program `vmatch` or `vmatchselect` in any directory. If you are using option `-selfun`, you have to specify the path to the directory where the shared object can be found. This is done by defining the environment variable `LD_LIBRARY_PATH`. For the `csh` or the `tcsh` you can, for example, define it as follows:

```
$ setenv LD_LIBRARY_PATH ".":"/usr/vstree/SELECT"
```

The corresponding definition for the `bash` or the `sh` is

```
$ LD_LIBRARY_PATH=".":"/usr/vstree/SELECT"
$ export LD_LIBRARY_PATH
```

This means that the shared objects are found in the current directory or in the directory

```
/usr/vstree/SELECT
```

The above example is a very simple selection function. The distribution of the programs described in this manual comes with a subdirectory `SELECT` containing more well-documented selection functions for diverse tasks.

10 `vmatchselect`: Sorting and Selecting Matches

`vmatchselect` allows to select interesting matches from the output of `vmatch` as specified by user-defined criteria. It delivers matches of chosen length, degeneracy or significance into further analysis routines. `vmatchselect` removes from the input all those matches that are contained in another match. To do this efficiently, the matches are sorted by the position in the database sequence, and hence in the order in which the matches are output, unless the user specifies otherwise. Moreover, the sequences of the virtual suffix tree for which the match file was produced can be clustered according to the matches. The input for `vmatchselect` is a file produced by `vmatch`, called a *match file*.

`vmatchselect` is called as follows:

`vmatchselect` [options] *matchfile*

The output of `vmatchselect` goes to standard output and is sorted in ascending order of the positions of the left instance of a matches. Two matches where the left instance occurs at the same position, are sorted in descending order of their length. Two matches of the same length where the left instance occurs in the same position, are sorted in ascending order of the position of the right instance of the match.

`vmatchselect` provides a subset of the options of `vmatch`, namely the following options:

-l	-leastscore	-evalue	-identity	-showdesc
-best	-sort	-selfun	-s	-absolute
-f	-nodist	-noevalue	-noscore	-noidentity
-dbcluster	-nonredundant	-v	-help	

The main difference to `vmatch` is that `vmatchselect` gets the matches from a *match file*, while `vmatch` computes the matches from scratch. Therefore options specifying the index and/or the query sequences to be matched, as well as options specifying how to match are not available in `vmatchselect`. The options of `vmatchselect` have the same meaning as in the program `vmatch`. Thus, for a description, see above.

Note that `vmatchselect` also allows to use the option `-dbcluster`. If `vmatchselect` is called with this option, then parses the given match file and performs single linkage clustering

based on the matches in this file. Thus `vmatch` and `vmatchselect` allow hierarchical clustering. In a first step an initial set of matches with loose matching criteria is computed, using `vmatch`. Then one clusters these matches by calling `vmatchselect`. In a second round one applies more strict choices for the matches by the using the options `-l`, `-leastscore`, `-evalue`, or `-identity`, etc. This allows stepwise refinement of clusters without much computational effort and no new index construction for the sequence of a cluster. The output of `vmatchselect` is the same as the output of `vmatch`.

11 Recent Changes

The following new features have recently been integrated into the software described in this manual.

2003-May-08; `mkvtree`: Option `-pl` can now be used without an argument. In this case, the value for *prefixlength* is determined automatically. If option `-pl` is used with an argument, then the behavior is as before. See page 9 for more details.

2003-May-15; `vsubseqselect`: The previous restriction on the maximum size of the substring to be selected was removed. Otherwise, the behavior of the program did not change. See page 22 for more details.

2003-May-17; `vmatch`, `vmatchselect`: Option `-iub` was removed. The effect of this option can be achieved by the argument *abbreviub* of option `-s`. See page 38 for more details.

2003-May-18; `vmatch`: The default speedup-parameter for matching a query against an index is now 2. That is, the option `-qspeedup 2` is implicitly set. As a consequence, table *stitab1* is required for the matching, i.e. it has to be created. However, by using `-qspeedup 0`, the matching works as before with additional tables, but probably not as efficient as with option `-qspeedup 2`. See page 33 for more details.

2003-May-31; `mkvtree`: `mkvtree` now scans the input files in GENBANK, EMBL or SWISSPROT format in such a way that additionally the `LOCUS`-information (in case of GENBANK) and the `ID`-information (in case of EMBL and SWISSPROT) is extracted and later reported as part of the description of the scanned sequence. See page 6 for more details.

2003-May-31; `mkvtree`: `mkvtree` can now read gzipped database files. These are internally unzipped and then processed as any other input files. See page 6 for more details.

2003-May-31; vmatch: vmatch can now read gzipped query files. These are internally unzipped and then processed as any other input files. See page 6 for more details.

2003-Jun-4; vseqselect: vseqselect now gives an error message when the chosen minimum length is larger than the chosen maximum length. See page 20 for more details.

2003-Jun-4; vmatch, vmatchselect: The argument *e* to option -evalue is now restricted to either be 0.0 or $\geq 1.0 \cdot 10^{-300}$. See page 36 for more details.

2003-Jun-15; vmatch, vmatchselect: The optional flags

keepleft, keepright, and keepleftifsamesequence

of option -dbnomatch have now been complemented by the flag keeprightifsamesequence. All four flags can now also be used for the option -dbmaskmatch. See page 29 for more details.

2003-Jun-28; vmatch, vmatchselect: The option -best has now been implemented with more efficient data structures. This gives a speedup if many matches are sought in order of their quality. See page 37 for more details.

2003-Jul-1; vsubseqselect: If option -range or option -seq is used, then the output shows the sequence description of the sequence, the substring was selected from. See page 22 for more details.

2003-Jul-2; vmatch, vmatchselect: Many new combinations of options are now possible. As a consequence, Table 6, contains considerably less entries of forbidden option combinations.

2003-Jul-12; vmatch, vmatchselect: Option -selfun can now have an arbitrary number of arguments, the first being the shared object containing the selection function bundle. All other arguments (not beginning with the symbol −) are ignored by vmatch and vmatchselect. See page 33 for more details.

2003-Jul-28; vstree2tex: Separator symbols are shown appropriately as different symbols. That is, the ith separator symbol is shown as number i. See page 18 for more details.

2003-Nov-13; all programs: Option -version added.

2004-Jun-18; `vmatch`, `vmatchselect`: Added new selection function bundle for reporting matches in CGviz-format. See the file `SELECT/cgvizout.c`. The selection function bundle was contributed by Katrin Wimmenauer. See page 81 for more details

2004-Jun-25; `vmatch`: When using the options `-dbmaskmatch` or `-qmaskmatch`, it is reported how many characters are masked. Previously, this information was shown on `stdout`. Now it is shown on `stderr`. See page 30 for more details.

2004-Aug-20; `vmatch`: Option `-online` now works properly in all possible combinations with other options. We extensively verified that it produces the same result as when running `vmatch` without this option. See page 33 for more details.

2004-Aug-21; `vmatch`: Many combinations of options that were not possible before now work properly. This mainly concerns the options `-i` and `-online`. As a consequence there are only 102 combinations of options in `vmatch` excluded (see Table 6), compared to 112 in the previous program version (which had even less options).

2004-Sep-03; `vmatch`, `vmatchselect`: In the `Sbjct`- and `Query`-lines showing an alignment, a position shown on the right end is now displayed correctly. In particular, gaps are ignored when counting the positions. This leads to smaller positions and makes the output more compatible with other programs.

2004-Sep-18; `vmatch`, `vmatchselect`: Modified the interface to function `selectmatchHeader`. Besides the parameter used for computing matches, the function also gets access to the parameters used in the call of the program using option `-selfun`, see Section E. See page 79 for more details.

2004-Sep-18; `vmatch`, `vmatchselect`: Added an extra function `selectmatchFinaltable` to the selection function bundle. It allows to e.g. display matches stored in a user defined table. See page 33 for more details.

2004-Sep-19; `vmatch`, `vmatchselect`: Added new selection function bundle for merging overlapping matches. See the file `SELECT/mergematches.c`. See page 82 for more details

2004-Oct-8; `vmatch`, `vmatchselect`: Changed the signature for the following functions of the selection function bundle: `selectmatchInit`, `selectmatch`, `selectmatchWrap`. These function are now called with three arguments:

- alphabet of the sequences to be compared,

- the Multiseq-structure representing the sequences in the index.
- the Multiseq-structure representing the sequences of the query.

See page 79 for more details.

2004-Oct-21; `vmatch`: When searching for repeats, option `-l` can now optionally be supplied with a second and third argument. Both arguments are integers specifying the minimum and maximum gap size of the repeat instances. See page 34 for more details.

2004-Dec-5; `mkvtree`: Given the indexname, `mkvtree` now removes all existing files comprising the index, before it generates the first index. This prevents from mixing index files from different runs of `mkvtree`. Furthermore, `mkvtree` now reports an error message, if an index file to be generated already exists.

2004-Dec-20; `mkvtree`: All symbol mappings for protein sequences now contain an additional wildcard symbol −, in addition to ⋆, to represent stop codons. This feature was suggested by Volker Brendel.

2004-Dec-22; `vmatch`: The maximum number of errors allowed with option `-h` and `-e` has been extended from 10 to 25. See page 35 for more details

2004-Dec-22; `vmatch`: Computing complete matches with errors is now much more convenient: options `-h` and `-e` have been extended to also allow the percentage search mode and the best search mode. In the percentage search mode the user only specifies the percentage of errors relative to the length of the query to be searched. In the best search mode, the user specifies that matches for a given query sequence are reported only for the minimum number of errors. See page 35 for more details

2004-Dec-22; `vmatch`: Computing complete matches with errors is now also possible on the index, without using option `-online`. For many combinations of error thresholds and query lengths this leads to reduced running times at the cost of increased space requirement.

2004-Dec-29; `vmatch`: If the given index is for sequences over the protein alphabet and the given query sequences are over the DNA alphabet, then one can use option `-dnavsprot`. This translates the DNA query sequences in all six reading frames. The six reading frames are matched against the protein database index, with all parameters allowed to control the kind of matches computed. The length of the matches and their positions are reported with respect to the original DNA sequence. An alignment for the matching substrings is shown on the protein level. See page 27 for more details

2004-Dec-31; `vmatch`: The recognition of symbol mappings for the DNA and the Protein alphabet has been improved. Now all symbol mappings in the subdirectory `TRANS` are correctly recognized. That is, all files in the directory `TRANS` with names containing the keyword `DNA` are recognized as DNA symbol mappings. All files in the directory `TRANS` with names containing the keyword `Prot` are recognized as Protein-symbol mappings. Of course, the recognition does not depend on the name of the symbol mapping files. See page 7 for more details

2005-Jan-18; `vmatch`: When using the options `-dbmaskmatch` or `-qmaskmatch`, it is reported how many characters are masked. If the environment variable `VMATCHCOMMENTTOSTDOUT` is set to the value `on`, then this information is shown on `stdout`. Otherwise it is shown on `stderr`. See page 30 for more details.

2005-Feb-1; `vmatch`: Option `-s` now allows an optional argument `xml`. This produces XML-output.

2005-Feb-21; `vmatch`: Recall that in the `vmatch`-output, the line beginning with the symbol `# args=` shows the arguments of `vmatch`. The last argument of a `vmatch`-call is the indexname. By default, the indexname is shown with its absolute path. If the environment variable `VMATCHRELATIVEINDEXPATH` is set to the value `on`, then the path before the indexname is omitted.

2005-Feb-25; `vmatch`: The option `-s abbreviub` now works correctly for all mismatching characters which are not wildcards.

2005-Feb-25; `vmatch`: The option `-mum` now allows an optional flag *cand* to compute *MUM*-candidates.

2005-Feb-28; `vmatch`: Fixed bug in the codon translation on the reverse strand.

2005-Feb-28; `vmatch`: Added program `mkdna6idx` to compute a six frame translation index. `vmatch` can now read such an index to perform a self comparison of the indexed DNA database sequences on the protein level.

2005-Feb-28; `vmatch`: Added program *chain2dim*. This computes global and local optimal chains. Added corresponding option `-pp chain` to directly compute chains in `vmatch`.

2005-Feb-28; `vmatch`: Added program *matchcluster*. This clusters matches. Added corresponding option `-pp matchcluster` to directly compute compute clusters in `vmatch`.

2005-Mar-11; mkvtree, vmatch: Added perl script `repfind.pl`. This allows to emulate the program `repfind` from the REPuter software package.

2005-Mar-15; vmatch: In former versions of the program, the maximum number of errors allowed with option `-h` and `-e` was 10 and later 25. This fixed maximum error number is removed now. That is you can choose any number of errors. See page 35 for more details

2005-Sep-13; vmatch: When combining option `-hxdrop` and `-leastscore`, `vmatch` did not show a match with 0, no matter what score it had. This was a bug reported by Martin Frith, and is now fixed.

2006-Apr-05; vmatchselect: When using `vmatchselect` with option `-s` applied to matchfiles containing query matches, the program produced a segmentation fault. This bug is now removed.

2006-Apr-12; mkvtree, vmatch: The sequences file parser previously tolerated sequences containing no symbols. This caused problems in several cases. Now `mkvtree` and `vmatch` produce an error message if empty sequences are detected. They exit with exit code 1.

2007-Jun-2; vmatch: Martin Frith pointed out that option `-supermax` delivers repeats which are not always supermaximal. The problem is fixed now, i.e. the algorithm for computing supermaximal repeats now works correctly.

2007-Jun-2; vmatch: Martin Frith pointed out that option `-i` combined with option `-supermax` delivers incorrect results. This also holds if option `-i` is combined with option `-mum` and self comparison is performed. This problems is fixed and `vmatch` now works correctly for these options.

2007-Nov-2; vmatch: Volker Brendel pointed out that in some cases `vmatch` reports different alignments for the same protein to genome comparison, depending on whether the protein query is supplied singly or together with other targets. This problem is fixed now.

2008-Feb-16; vmatch: Anar Khan reported that Vmatch exits with an error message, when reverse complemented repeats are computed and constraints on the distance of the matches are specified. This problem is fixed now.

2010-Apr-26; `vmatch`: option `-nonredundant` now delivers the longest sequence from the corresponding cluster (instead of an unspecified representative). For every binary the distribution now contains an additional statically linked binary with the same name in the directory static/ (except for Mac OS X).

Acknowledgements

Sven Rahmann, Kirstin Weber, Volker Brendel, Alexander Sczyrba, Jomuna Choudhuri, Gordon Gremme, Marisano James, Martin Frith, Thomas Jahns, Peter Andrews, Wei Zhu, Anar Khan, and Jeremy Leipzig gave valuable hints on improving the manual and the programs described herein. Their help is much appreciated.

12 References

[1] M.I. Abouelhoda, S. Kurtz, and E. Ohlebusch. The enhanced suffix array and its applications to genome analysis. In *Proceedings of the Second Workshop on Algorithms in Bioinformatics*, pages 449–463. Lecture Notes in Computer Science 2452, Springer-Verlag, 2002.

[2] M.I. Abouelhoda, S. Kurtz, and E. Ohlebusch. Replacing suffix trees with enhanced suffix arrays. *Journal of Discrete Algorithms*, 2:53–86, 2004.

[3] M.I. Abouelhoda and E. Ohlebusch. A Local Chaining Algorithm and its Applications in Comparative Genomics. In *Proc. 3rd Worksh. Algorithms in Bioinformatics (WABI 2003)*, number 2812 in Lecture Notes in Bioinformatics, pages 1–16. Springer-Verlag, 2003.

[4] O. Delgado-Friederichs, T. Dezulian, and D.H. Huson. A meta-viewer for biomolecular data. In R.K. Dittrich, W. König, A. Oberweis, K. Rannenberg, and W. Wahlster, editors, *INFORMATIK 2003 – Band 1. GI Edition Lecture notes in Informatics, Vol. P-34*. Gesellschaft für Informatik, 2003.

[5] M. Höhl, S. Kurtz, and E. Ohlebusch. Efficient multiple genome alignment. *Bioinformatics*, 18(Suppl. 1):S312–S320, 2002.

[6] S. Kurtz. A Time and Space Efficient Algorithm for the Substring Matching Problem, 2002.

[7] S. Kurtz, J.V. Choudhuri, E. Ohlebusch, C. Schleiermacher, J. Stoye, and R. Giegerich. REPuter: The manifold applications of repeat analysis on a genomic scale. *Nucleic Acids Res.*, 29(22):4633–4642, 2001.

[8] U. Manber and E.W. Myers. Suffix Arrays: A New Method for On-Line String Searches. *SIAM Journal on Computing*, 22(5):935–948, 1993.

[9] G. Myers. A Fast Bit-Vector Algorithm for Approximate String Matching Based on Dynamic Programming. *Journal of the ACM*, 46:395–415, 1999.

[10] E. Ukkonen. Algorithms for Approximate String Matching. *Information and Control*, 64:100–118, 1985.

[11] N. Volfovsky, B.J. Haas, and S.L. Salzberg. A Clustering Method for Repeat Analysis in DNA Sequences. *Genome Biology*, 2(8):research0027.1–0027.11, 2001.

[12] Z. Zhang, S. Schwartz, L. Wagner, and W. Miller. A Greedy Algorithm for Aligning DNA Sequences. *J. Comp. Biol.*, 7(1/2):203–214, 2000.

A Basic Notions

Throughout this section we consider a sequence u of length m over some alphabet Σ. The symbols are indexed from 0, i.e. $u = u_0u_1 \dots u_{m-1}$.

If Σ is the DNA alphabet $\{a,c,g,t\}$, then we define a function $wcc : \Sigma \to \Sigma$ by the following equations:

$$\begin{aligned} wcc(a) &= t \\ wcc(g) &= c \\ wcc(c) &= g \\ wcc(t) &= a \end{aligned}$$

We extend wcc to any sequence $u = u_0u_1 \dots u_{m-1}$ over Σ by defining

$$wcc(u) = wcc(u_0)wcc(u_1)\dots wcc(u_{m-1})$$

Consider two sequences u and v of equal length. The *Hamming distance* of u and v, denoted by $d_H(u,v)$, is the number of positions where u and v differ, that is,

$$d_H(u,v) = |\{i \in [1,|u|] \mid u_i \neq v_i\}|$$

There are three kinds of edit operations: *deletions*, *insertions*, and *mismatches* of single symbols. The *edit distance* or *Levenshtein distance* of u and v, denoted by $d_E(u,v)$, is the minimum number of edit operations needed to transform u into v.

Let $\approx$ be a binary relation on sequences, and assume that u and v are sequences of length m and n. Let $l,r > 0$, $i \in [0,m-l]$, and $j \in [0,n-r]$. We define two different *kinds* of matches between u and v:

- (l,i,r,j) is a *direct match* if and only if

  $$u_iu_{i+1}\dots u_{i+l-1} \approx v_jv_{j+1}\dots v_{j+r-1} \tag{1}$$

  If $u = v$, then we additionally assume $i < j$.

- (l,i,r,j) is a *palindromic match* if and only if

  $$u_iu_{i+1}\dots u_{i+l-1} \approx wcc(v_{j+r-1}v_{j+r-2}\dots v_j) \tag{2}$$

  If $u = v$, then we additionally assume $i \leq j$.

(l,i,r,j) is a *match*, if it either is a direct or a palindromic match. Each match (l,i,r,j) specifies two sequences:

1. the sequence $u_i u_{i+1} \dots u_{i+l-1}$ occurring on the left-hand side of (1) or (2). This is the *left instance* of (l,i,r,j).
2. the sequence occurring on the right-hand side of (1) or (2). This is the *right instance* of (l,i,r,j).

By specifying the relation $\approx$, we obtain different types of matches:

1. If $\approx$ is the identity on sequences, then a match (l,i,r,j) is an *exact match*.
2. Let $k > 0$. If for any sequences x,y the relation $\approx$ satisfies

$$x \approx y \iff |u| = |v| \text{ and } d_H(x,y) \leq k$$

   then a match (l,i,r,j) is a *k-mismatch match*.
3. Let $k \in \mathbb{N}$. If for any sequences x,y the relation $\approx$ satisfies

$$x \approx y \iff d_E(x,y) \leq k$$

   then a match (l,i,r,j) is a *k-differences match*.
4. A match is *degenerate* if it is a k-mismatch or a k-differences match for some $k \geq 0$.

To distinguish between the different types of matches, we assign to them a *distance value* according to the following rules:

- An exact match has distance 0.
- A k-mismatch match with $k > 0$ mismatches has distance $-k$.
- A k-differences match with $k > 0$ differences has distance k.

A match (l,i,r,j) is *contained* in a match (l',i',r',j') if and only if $i' \leq i \leq i+l-1 \leq i'+l'-1$ and $j' \leq j \leq j+r-1 \leq j'+r'-1$.
By requiring matches to be *maximal* we can reduce the number of interesting matches:

- An exact match is *maximal* if it is not contained in another exact match of the same kind.
- A k-mismatch match is *maximal* if it is not contained in another k-mismatch match of the same kind.
- A k-differences match is *maximal* if it is not contained in another k-differences match of the same kind.

A *maximal unique match* is a sequence w with the following properties:

- $|w| \geq l$.
- w occurs exactly once in u and it occurs exactly once in v.
- For any character a neither wa nor aw occurs both in u and in v.

w is represented by its length and its start positions in u and in v.

Example: Let $u = gattcttcgt$, $v = cctcgtgtcg$, and $l = 2$. Then there are two maximal unique matches ct and $tcgt$ represented by $(2,4,1)$ and $(4,6,2)$.

When comparing a sequence against itself, we also call a match (l,i,r,j) a repeat, if $i < j$. In other words, the two instances of a match do not occur at the same position. In this way, we do not report the trivial repeat where both instances of the match is the sequence itself.

Consider a repeat. If the left and the right instance of the repeat directly follow each other (without a gap or overlap), then the repeat is called *tandem repeat*. Suppose that the left instance of the tandem repeat starts at position p and the repeat is of length l. Then the right instance starts at position $p+l$. Such a tandem repeat is *branching* if the characters at position $p+l$ and $p+2l$ are distinct.

A *supermaximal repeat* is a maximal repeat that never occurs as a substring of any other maximal repeat.

B Specification of a symbol mapping

`mkvtree` can be applied to sequences over any set of printable ASCII symbols. So, in particular it can be used for DNA, RNA, and for protein sequences. The user specifies in a symbol mapping the symbols the alphabet consists of (including wildcards). By grouping the symbols on different lines, the user also defines which symbols are considered to be identical, and how the symbols should be output when reporting a match. The symbol mapping implicitly defines an alphabet transformation φ, which is applied to the input file. *mapsize* is the number of lines in the symbol mapping files. If a symbol mapping is given, then all symbols in the input files must occur in the alphabet. Otherwise, the program exits with error code 1.

The format of the symbol mapping file is best explained by some examples. Consider the file `TransDNA` containing the following lines:

```
aA
cC
gG
tTuU
nsywrkvbdhmNSYWRKVBDHM
```

These lines specify that the sequences in the input files are allowed to contain the symbols $a,c,g,t,u,n,s,y,w,r,k,v,b,d,h,m$ in either lower or upper case. Moreover, the first four lines

specify that, whenever characters are compared, the following equalities hold: $a = A$, $c = C$, $g = G$, and $t = T = u = U$. The last line specifies the wildcard symbols, which are replaced by unique symbols. As an effect, for each two (not necessary different) symbols α and β appearing on the last line, we have $\alpha \neq \beta$ and $\alpha \neq a$, $\alpha \neq A$, ..., $\alpha \neq u$, $\alpha \neq U$.

The internal symbol mapping φ is as follows: the symbols on the first line are mapped to 0, the symbols of the second line to 1, etc. However, the symbols on the last line are mapped to the constant `WILDCARD`, which is a non-printable ASCII character. When matching symbols, each occurrence of `WILDCARD` is handled in such a way, that it does not match anywhere. Thus no false exact matches containing a wildcard will be delivered. However, a degenerate match may contain a `WILDCARD`, but this always leads to a pair of mismatching characters, or it must be deleted or inserted.

When matches are reported, the sequence output (if any) usually consists of the first symbol of each line, except if on that line there is a symbol following a white space. For example, if a match contains the symbol `A` in the original input file, then this match is reported as `a` in the output of `vmatch`. So if the user wants to output capital letters, he/she has to reverse the columns in the symbol mapping file.

Here is another example (see file `TRANS/TransProt11`), where different groups of amino acids are mapped to single symbols. We have also added comment lines. Each line at the beginning of the file starting with the the symbol `#` is considered a comment line. It is ignored when parsing the symbol mapping file. Any number of comment lines is allowed, but only at the beginning of the file.

```
# 11-character alphabet as defined by Volker Brendel
LVIFJ i
KR +
ED -
AG s
ST o
NQ n
YW a
P p
H h
M m
C c
XUBZO*- x
```

This specifies the protein alphabet $L,V,I,F,K,R,E,D,A,G,S,T,N,Q,Y,W,P,H,M,C$. Some extra wildcard symbols $X,B,Z,*,-$ are specified on the last line. All symbols occurring on the same line to the left of the first white space (if any) are considered to be equivalent. In each match, the symbol after the first white space will be shown for each symbol to its left. The symbols on the last line are considered to be wildcards. Again they are replaced by the symbol `WILDCARD`. The implicit mapping φ maps the symbol on line number $i \in [0, mapsize - 1]$ to integer i. In our case, L,V,I,F are mapped to 0, K,R are mapped to 1, etc.

C The X-Drop Extension Strategy

Consider the problem of aligning initial portions of two sequences, say u and v of length m and n, respectively. Alignments are scored such that each mismatch has score -1, an indel (i.e. insertion or a deletion) has score -2, and each match has score 2. The goal is to find a highest-scoring (i.e. optimal) alignment of some prefix of u and some prefix of v, which are chosen to maximize the score. Here m is the length of u and n is the length of v. There is a simple method to compute such an optimal alignment, but this method is inefficient, since it requires $O(mn)$ time. Zhang et. al. have proposed a method to prune the quadratic search space. This method, called *greedy algorithm* in the sequel, does not guarantee that the highest scoring alignment is found. However, in practice the alignments reported score very close to the optimal alignment. The greedy algorithm requires to specify a positive integer X which influences the size of the evaluated search space. The smaller X, the smaller the evaluated search space. The greedy algorithm keeps track of the best alignment it has seen so far. Suppose this has score T. Whenever it extends an alignment, it checks whether the score of the extended alignment is smaller than $T-X$. If this is the case, than the extension is discarded. This effectively prunes the search space. The width of the search space expands in regions where the aligned sequence parts are different while it reduces in regions where the aligned sequences are similar. As a consequence, the greedy algorithm terminates if a similar part is followed by a region of many differences. The integer X determines how much differences are tolerated.

If you use the program `vmatch` with the option `-exdrop`, then the *Xdrop*-alignment strategy is used when extending seeds (i.e. maximal exact matches) to the left and to the right. More precisely, if a maximal exact match (l,i,r,j) of sequences u and v is found, then it is extended by applying the greedy algorithm twice:

- It is applied to the reverse of the two sequences $u_0u_1\dots u_{i-1}$ and $v_0v_1\dots v_{j-1}$
- It is applied to the two sequences $u_{i+l}u_{i+l+1}\dots u_m$ and $v_{j+r}v_{j+r+1}\dots v_n$

The two alignments reported by the greedy algorithm are concatenated with the seed to form an alignment of the substrings $u_{i'}\dots u_{i''}$ and $v_{j'}\dots v_{j''}$ where $i'\leq i$, $i+l-1\leq i''$, $j'\leq j$, and $j+r-1\leq j''$.

D Substring Specifications

There are applications where one wants to restrict the matching process to a substring of the query or to a substring of an database sequences. For example, if one already knows that the matches occur in a certain region of the database or the query, and one wants to allow less stringent matching conditions without spending time to search for matches in other regions. For these applications, `vmatch` allows two kinds of substring specifications:

- If only one query file with exactly one sequence, say s, is used as an argument to option `-q`, then the query file may optionally be followed by an extra argument of the form (i,j). i and j are integers in the range $[0,|s|-1]$ such that $i<j$ or $j=0$. If $j=0$, then this is interpreted as $|s|-1$. That is, let $j'=j$ if $j>0$ and $j'=|s|-1$, otherwise. The pair (i,j) specifies that the matching is to be restricted to the query substring $s_i \ldots s_{j'}$. Note that in some Unix-shells the symbols `(` and `)` have special meaning. Hence it may be necessary to quote the argument (i,j), so that the shell does not evaluate it. For example, single quotes work for the `csh`, the `tcsh`, the `sh`, and the `bash`. The matches reported are as before, with the exception of the E-value. This is reported with respect to the shorter query substring, and thus becomes smaller. This feature does not work in connection with the option `-online`.

- If the options `-online` and `-q` are used, then `vmatch` checks whether the descriptions of the query sequences begin with `>@` $i\,j$ where i and j are numbers such that $0 \leq i$ and $i<j$ or $j=0$. If this is the case for *all* descriptions of the query sequences, then the index is allowed to only contain one sequence, say t. If this is the case, then for each substring specification `>@` $i\,j$ the matching is restricted to the substring $t_i \ldots t_{j'}$ where $j'=j$, if $j>0$ and $j'=|t|-1$, otherwise. The matches reported are as before, with the exception of the E-value. This is reported with respect to the shorter database substring, and thus becomes smaller. Note that the substring specifications are only taken into account if the option `-online` is used.

E Selection Function Bundles

Although `vmatch` allows many different ways to postprocess matches or to modify the output, the wide range of option combinations may not deliver what is required in a special application. The standard approach, used for example for BLAST, works as follows: (1) output the matches to a file, (2) parse this file with a user written program, and (3) postprocess the matches as required. However, this approach has several disadvantages:

- The user has to write a program to parse the output of the search engine. Even if the output format is designed s.t. it is easy to parse, this still requires extra effort.

- The size of the output can be huge for large scale matching problems. Due to slow I/O processing, output and parsing the matches can often take more time than computing them.

- It is often not only the values reported in the output of a typical search that is relevant for postprocessing, but also the set of sequences (with the sequence content and the corresponding sequence descriptions) subject to the matching task. Hence extra effort is required to parse the input sequences in such a way that the postprocessing program has access to the sequence information.

To circumvent these disadvantages, vmatch provides the concept of *selection functions*. These allow flexible and fast on-the-fly postprocessing of the matches computed by vmatch, without extra effort for output and parsing of the matches and sequence information.

Selection functions allow to access the matches and the sequences involved in the match immediately after they are computed. The selection is applied before further postprocessing or output is done, so that all other postprocessing options can be combined with selection functions.

Technically, a selection function is specified by a bundle of functions written in the programming language C. The program code implementing the selection functions is compiled independently from vmatch, but based on the data structures used in vmatch. Using the option -selfun, vmatch calls the selection functions via shared library calls (sometimes also called dynamic libraries). Since the definition of a selection function bundle requires a basic knowledge in C-programming, this concept is probably not suited for the average user. Nevertheless, we describe this concept carefully to give an idea of the flexibility it allows.

A *selection function bundle* consists of four functions with the following function headers:

```
Sint selectmatchHeader(int argc,
                       char **argv,
                       int callargc,
                        char **callargv)

Sint selectmatchInit(Alphabet *alpha,
                     Multiseq *virtualmultiseq,
                     Multiseq *querymultiseq)

Sint selectmatch(Alphabet *alpha,
                 Multiseq *virtualmultiseq,
                 Multiseq *querymultiseq,
                 StoreMatch *storematch)

ArrayStoreMatch *selectmatchFinaltable(Alphabet *alpha,
                                       Multiseq *virtualmultiseq,
                                       Multiseq *querymultiseq);

Sint selectmatchWrap(Alphabet *alpha,
                     Multiseq *virtualmultiseq,
                     Multiseq *querymultiseq)
```

Sint is a type synonym for signed integers. The C-structures Multiseq and StoreMatch are defined in different header files. These are part of the distribution of the programs described in this manual.

Now suppose that these functions are specified in an extra file, and that they are compiled in the appropriate way (see below). Then the following holds:

- The function selectmatchHeader is called *before* the index and the query sequences (if any) are read. The first argument of the function is the number of arguments of the

corresponding call of `vmatch` and the second argument is the corresponding argument vector storing pointers

$$\texttt{argv}[0],\ldots,\texttt{argv}[\texttt{argc}-1]$$

to the arguments of `vmatch`. In a call of `vmatch`, we have `callargc=argc` and `callargv=argv`. In a call of `vmatchselect`, `callargc` is the number of arguments of the program call to `vmatchselect`. `callargv` is the corresponding argument vector.

- The function `selectmatchInit` is called *after* the index and the query files (if any) are read and *before* the first match is processed. The indexed database sequences can be accessed via the pointer `virtualmultiseq`, while the query sequence is accessed via the pointer `querymultiseq`. The latter is `NULL` if the index is compared to itself. This function can e.g. be used to initialize internal data structures defined by the user.

- The function `selectmatch` is applied to each match referenced by `storematch`.
  - If `selectmatch` returns a value smaller than 0, then this is interpreted as an error, and `vmatch` or `vmatchselect` exits with error code 1.
  - If `selectmatch` returns 0, then this is interpreted as *false* and the match pointed to by `storematch` is not selected. That is, it is discarded.
  - If `selectmatch` returns 1, then this is interpreted as *true* and the match pointed to by `storematch` is selected. That is, it is output or postprocessed by `vmatch` according to the options given.

- The function `selectmatchFinaltable` is called *after* the last match has been processed, but before `selectmatchWrap` is called. If specified by the user, it must return a pointer to a memory area which is available after the call to this function. The memory area stores an array of structures of type `StoreMatch`. See the selection function bundle in `mergematches.c` for an example. `vmatch` or `vmatchselect` accesses the memory area and processes it according to the options given by the user. In the standard case, the merged matches are shown on standard out.

- The function `selectmatchWrap` is called *after* the last match has been processed. It can, e.g., be used to output internal data structures which store accumulated information about the matches found.

A selection function bundle must define at least the function `selectmatch`. The other functions can be omitted in the C-file declaring the selection function bundle.

The distribution of the programs described in this manual comes with a subdirectory `SELECT` containing well-documented selection functions for diverse tasks:

cgvizout.c Output matches in a format readable for the visualization program CGviz, see [4].

dbseqstat.c counts the number of matches to different query sequences and outputs all database sequences containing at least two matches to different query sequences.

endmatch.c selects all matches which start with the first character of a database sequence or which end with the last character of a database sequence.

lowcomplex.c discards all matches where the matching sequences are of low complexity, according to a simple model based on the distribution of characters.

mstat.c groups matches according to their occurrence in the database sequences.

mergematches.c merge two matches if they overlap by a significant number of positions. This number is specified as some percentage of the length of the matches.

rightmost.c selects the rightmost matches in all database sequences and discards those matches which are not rightmost.

sel392.c selects matches which are not longer than some maximum value (see above). The latter can be defined at run time by the user.

selsplicesite.c selects matches where the right instance of the match (in the query) does not have `AG` to the right and `TC` to the left.

selstartend.c select matches where the left instance begins with the bases `AC` and ends with `GT`.

xmlout.c shows the numeric information of the matches (not the alignment or other sequence information) in XML-format.

Besides these files, subdirectory `SELECT` also contains a `makefile`, specifying how to compile a shared object for the supported platforms.

F The Tables Comprising the Index

Suppose the input files contain the sequences $T_1, \dots, T_k$ in this order. Let # be a symbol not occurring in any T_i and suppose $S = T_1 \# T_2 \# \dots T_{k-1} \# T_k$. That is, # is used as a separator symbol. Let $n = |S| = k - 1 + \sum_{i=1}^{k} |T_i|$. The index consists of the tables *prjtab*, *tistab*, *oistab*, *suftab*, *bwttab*, *lcptab*, *llvtab*, *skptab*, *bcktab*, *ssptab*, *destab*, *sdstab*.

These are defined as follows:

- *prjtab* contains the "‘project information"’, i.e. some basic information about the index in human readable form. Here is an example:

```
dbfile=atEST 999815 772376
totallength=772376
numofsequences=1952
numofdbsequences=1952
numofquerysequences=0
prefixlength=7
largelcpvalues=2317
maxbranchdepth=517
integersize=32
```

This information shall be read as follows: There is only one database file `atEST` of length 999815. The total length of the sequences in this file plus the separator symbols between the sequences is 772376. The total length of all database sequences and their number is given in the next two lines. The construction of the index was done using an initial bucket sorting step with *prefixlength* set to 7. The number of *lcp*-values (see below) larger or equal to 255 is 2317, and the largest *lcp*-value is 517. The last line tells that the index has been constructed by a version of mkvtree using 32-bit integer.

- *tistab* is the *transformed input sequence* (thus the abbreviation *tis*). In particular it is a table of length n such that, for any $i \in [0, n-1]$,

$$tistab[i] = \begin{cases} \texttt{SEPARATOR} & \text{if } S_i = \# \\ \varphi(S_i) & \text{otherwise} \end{cases}$$

  Here `SEPARATOR` is a non-printable ASCII-character different from `WILDCARD`. Each entry in *tistab* is stored in 1 byte.

- *oistab* is the original input sequence after parsing (thus the abbreviation *ois*). In particular it is a table of length n such that, for any $i \in [0, n-1]$,

$$oistab[i] = \begin{cases} \texttt{SEPARATOR} & \text{if } S_i = \# \\ S_i & \text{otherwise} \end{cases}$$

  Each entry in *oistab* is stored in 1 byte.

- *suftab* is a table of length $n+1$. To define it, we need some preliminaries: *tistab* can be interpreted as a sequence, say T, of length n. Let \$ be a new symbol greater than any symbol in T. Consider the string $T\$$ and let $X_i = T_i \dots T_{n-1}\$$ for any $i \in [0, n]$. That is, X_i is the suffix of $T\$$ starting at position i. Suppose $X_{j_0}, X_{j_1}, \dots, X_{j_n}$ is the sequence of suffixes in ascending lexicographic order. Then table *suftab* of length $n+1$ is defined by $suftab[i] = j_i$ for any $i \in [0, n]$. If $n < 2^{32}$, then each entry in *suftab* is stored in 4 bytes. Otherwise, it is stored in 8 bytes.

- *stitab1* is the reduced version of the inverse suffix table. It is a table of length $n+1$. To define it, we have to introduce table *stitab*. This is the inverse of table *suftab*, i.e. it

satisfies the equality $suftab[stitab[i]] = i$ for all $i \in [0,n]$. Now suppose that $stitab[i] \in [bcktab[w], bcktab[w]-1]$ for some string w of length *prefixlength*. Then

$$stitab1[i] = \begin{cases} 255 & \text{if } stitab[i] - bcktab[w] \geq 255 \\ stitab[i] - bcktab[w] & \text{otherwise} \end{cases}$$

Each entry in *stitab1* is stored in 1 byte.

- *bwttab* is a table of length $n+1$ such that, for any $i \in [0,n]$,

  $$bwttab[i] \quad = \quad \begin{cases} \text{undefined} & \text{if } suftab[i] = 0 \\ tistab[suftab[i]-1] & \text{otherwise} \end{cases}$$

  Each value in *bwttab* is stored in 1 byte. *bwttab* is the Burrows and Wheeler transformation.

- *lcptab* is a table of length $n+1$. Suppose that for any $i \in [1,n]$, $lcp(i)$ is the length of the longest common prefix of the suffix $T_{suftab[i-1]}$ and $T_{suftab[i]}$. If $lcp(i) < 255$, then $lcptab[i] = lcp(i)$. Otherwise, $lcptab[i] = 255$ and in table *llvtab* we store the pair $(i, lcp(i))$. In other words, *llvtab* stores the large lcp values. The pairs in table *llvtab* are ordered according to their left component. $lcptab[0]$ is undefined. Each entry in *lcptab* is stored in one byte. If $n < 2^{32}$, then each entry in *llvtab* is stored in 8 bytes. Otherwise, it is stored in 16 bytes.

- *skptab* is a table of length $n+1$ such that, for any $i \in [0,n]$,

  $$skptab[i] = \min(\{n\} \cup \{j \in [i+1,n] \mid lcp(i) > lcp(j)\})$$

  The abbreviation `skp` stands for skip-value. If $n < 2^{32}$, then each entry in *skptab* is stored in four bytes. Otherwise it is stored in 8 bytes.

- For some value $prefixlen > 0$ as specified in *prjtab*, table *bcktab* stores the bucket boundaries. These are defined as follows: Let $r = mapsize - 1$, $l = prefixlen$, and $\Sigma = [0, r-1]$. For any sequence $w \in \Sigma^l$, define $\kappa(w) = \sum_{i=0}^{l-1} r^{l-1-i} w_i$. $\kappa : \Sigma^l \to [0, r^l - 1]$ is a bijective function, mapping each $w \in \Sigma^l$ to the corresponding integer to base r. For each $w \in \Sigma^l$, $bcktab[\kappa(w)]$ stores a pair of integers $(p, q+1)$ such that:

  - $T_{suftab[p]}$ is the lexicographically smallest suffix of T which has w as a prefix
  - $T_{suftab[q]}$ is the lexicographically largest suffix of T which has w as a prefix

  If w does not occur as a substring of T, then $p = q+1$. *bcktab* is of size r^l. If $n < 2^{32}$, then each entry in *bcktab* is stored in 8 bytes. Otherwise, it is stored in 16 bytes.

- *ssptab* is a table of length $k-1$ storing, in sorted ordered, the positions in S where the separator symbol # occurs. If $n < 2^{32}$, then each entry is stored in four bytes. Otherwise, it is stored in 8 bytes. The abbreviation *ssp* stands for *sequence separator positions*.

- *destab* stores the concatenated descriptions from the input files including a separator `\n`.

Table 7: Overview of the tables comprising the index. n is the total length of all database sequences including the $k-1$ separator symbols. k is the number of database sequences. r is *mapsize* -1 and *mapsize* is the number of lines in the symbol mapping file. The size of each entry is given in bytes, assuming that $n < 2^{32}$. If $n \leq 2^{32}$, then the size of each entry in table *suftab*, *llvtab*, *skptab*, *bcktab*, and *ssptab* is twice as large. If any of the options `-smap`, `-dna`, or `-protein` was used for `mkvtree`, then there exists a corresponding table *al1tab* storing the symbol mapping. Otherwise, all symbols contained in the file are stored in *al2tab*.

table	description	length	size per entry
prjtab	project information	a few lines	
tistab	transformed input sequences	n	1
oistab	original input sequences	n	1
suftab	suffix array	$n+1$	4
stitab1	reduced inverse suffix array	$n+1$	1
bwttab	Burrows Wheeler Transformation	$n+1$	1
lcptab	longest common prefixes < 255	$n+1$	1
llvtab	longest common prefixes ≥ 255	$\lvert\{i \in [1,n] \mid lcp(i) \geq 255\}\rvert$	8
skptab	skip values	$n+1$	4
bcktab	bucket boundaries	r^l	8
ssptab	sequence separator positions	$k-1$	4
destab	descriptions for sequences	sum of description lengths	
sdstab	start pos. descriptions	$k+1$	4
al1tab	symbol mapping used	a few lines	
al2tab	enumeration of symbols	≤ 255	

- *sdstab* is a table of length $k+1$ storing the starting positions of the descriptions of the sequences. That is, for any $i \in [0,k-1]$, the description of sequence i is stored from position $sdstab[i]$ to $sdstab[i+1]-1$. If $n < 2^{32}$, then each entry is stored in four bytes. Otherwise, it is stored in 8 bytes.

Table 7 gives an overview of the different tables.

To satisfy the different matching tasks, different tables are required, according to the following rules:

self comparison: If only the number of exact substring matches is to be reported, then *bwttab* and *lcptab*. Otherwise, *lcptab*, *suftab*, and additionally:

- *bwttab*, if direct matches are to be reported.
- *tistab*, if degenerate matches or palindromic matches or the string content of a match is to be reported.

- *bcktab*, if palindromic matches are to be reported.

query comparison: If the matching task is to be performed online, then *tistab*. Otherwise, *lcptab*, *tistab*, *suftab*, and *bcktab*.

The following additional rules apply. In all cases, *destab*, *sdstab*, and *prjtab* are required. If there is more than one sequence in the index, then also *ssptab*. If *lcptab* is required, then also *llvtab*. Table *skptab* is currently not required for any application implemented in `vmatch`.

G Environment Variables

There are some environment variable which allow to influence the behavior of the programs described here:

- `MKVTREESMAPDIR`: is a colon separated list of directories to search for symbol map files.
- `VMATCHCOMMENTTOSTDOUT`: if set to the value `on`, then the line showing how much symbols are masked (for option `-dbmaskmatch` or `-qmaskmatch`) is shown on stdout. If set to `off`, then the line is shown on stderr.
- `VMATCHRELATIVEINDEXPATH`: The last argument of a `vmatch`-call is the indexname. By default, the indexname is shown with its absolute path. If this environment is set to the value `on`, then the path before the indexname is omitted.
- `LD_LIBRARY_PATH`: colon separated list of directories, where to find the shared object files used as arguments for the option `-selfun`.

Index

t, 17, 27
.fna, 31, 59
.match, 31, 59
.single, 31
.sl, 33
.so, 33
cand, 29
chain, 32
matchcluster, 32
remred, 29

abbrev, 38
abbreviub, 38, 66
alignment, 26, 31, 36–38, 43
  high scoring, 77
  optimal, 77
alphabet, 4
  DNA, 4, 73
  protein, 4
Arabidopsis, 11
atEST, 11, 17, 21, 23, 40, 48, 57
atEST.skp, 12

bac, 13
bcktab, 83
best mode, 35, 36
BLAST, 78
bwttab, 83
byte order, 24

cand, 70
chain-parameter, 32
chain-parameters, 32
cluster, 30, 57
  singleton, 31
Codonseq, 54
Codonseq.fna, 53
comment, 76
Compaq-Alpha, 4, 64
comparison
  query, 85
  self, 84

D, 42
database, 4
dbfiles, 7, 16
DE, 6, 15
DEFINITION, 6
deletion, 25, 36, 73
description
  EMBL, 6, 66
  FASTA, 6
  GENBANK, 6, 66
  SWISSPROT, 6, 66
destab, 83
distance
  edit, 73
  hamming, 73
  Levenshtein, 73
  value, 74
dnavsprot, 69

E-value, 26, 42
Ecoli K12, 55–57, 60
Ecoli O157:H7, 12, 45, 46, 52, 55, 56, 60
EcoliO157H7, 12, 56, 57
edit distance, 36
EMBL, 6
endian
  big, 24
  little, 24
error code, 6, 8, 11, 16, 27, 33, 75, 80
excluding options, 40
exit code, 10, 19, 21, 23, 39
extraargs, 33

F, 53, 54
file
  binary, 24
  cluster, 59
  database, 7
  index, 4
  input, 4
  match, 31, 59
  output, 18
filename, 20, 31, 33
filenameprefix, 30, 31

filepath, 9, 17
flag, 29, 30, 38

G, 53, 54
gcc, 64
GENBANK, 6
greedy, 26
greedy algorithm, 77
gzip, 66

H, 54
hamming, 35

I, 54
ID, 6, 15, 66
identity value, 26, 43
index, 4
indexname, 24, 26
input
    format, 6
    gzipped, 6, 27
insertion, 25, 36, 73
IUB, 38

keepleft, 29, 67
keepleftifsamesequence, 29, 67
keepright, 29, 67
keeprightifsamesequence, 29, 67

largeheading, 49
LaTeX, 18, 19
lcptab, 83
LD_LIBRARY_PATH, 64, 85
LD_LIBRARY_PATH, 33
leftseq, 44
leftseq, 38
Linux, 4, 24, 64
LOCUS, 6, 66
low complexity, 81

M. genitalis, 13
M. pneunomiae, 13
mapfile, 7, 8, 16, 27
match
    complete, 25
    containment, 74
    degenerate, 25, 74
    differences, 74
    direct, 25, 42, 73
    exact, 25, 74
    extension, 26
    instance, 42, 74
    kind, 25
    maximal, 74
    maximal unique, 25, 29, 50, 74
    maximal unique candidate, 29
    mismatch, 74
    palindromic, 25, 42, 73
    postprocessing, 26
    query, 48
    substring, 25
    unique, 55
match score, 26
matchcluster-parameter, 32
matchfile, 65
maxclsize, 30, 31
maxgapsize, 34
maxlength, 39
mfdindex, 59
mgen.fna, 13
minclsize, 30, 31
mingapsize, 34
mismatch, 25, 35, 36
mkvtree, 4
MKVTREESMAPDIR, 7, 16, 85
mode, 37
mpneu.fna, 13
multiple fasta, 6
Multiseq, 79
myseqnum, 22

oistab, 82
Option
    -absolute, 39
    -allmax, 36
    -allout, 10
    -bck, 10, 19
    -best, 37
    -bwt, 10, 19
    -complete, 29
    -dbcluster, 30
    -dbmaskmatch, 30
    -dbnomatch, 29

-db, 7, 16
-dnavsprot, 27
-dna, 8
-d, 34
-evalue, 37
-exdrop, 36
-e, 35
-f, 39
-help+, 39
-help, 10, 17, 19, 21, 23, 39
-hxdrop, 36
-h, 35
-identity, 37
-indexname, 9, 17
-i, 37
-lcp, 10, 19
-leastscore, 36
-l, 34
-maxlength, 20, 22
-minlength, 20, 22
-mum, 29
-nodist, 39
-noevalue, 39
-noidentity, 39
-nonredundant, 31
-noscore, 39
-ois, 9, 17, 19
-online, 33
-pl, 9
-pp, 32
-protein, 8
-p, 34
-qmaskmatch, 30
-qnomatch, 30
-qspeedup, 33
-q, 27
-randomlength, 21
-randomnum, 21
-range, 23
-seedlength, 36
-selfun, 33
-seqnum, 20
-seq, 23
-showdesc, 38
-skp, 10, 19
-smap, 7, 16
-snum, 23
-sort, 37
-stil, 9, 19
-suf, 9, 19
-supermax, 27
-s, 19, 38
-tandem, 27
-tis, 9, 17, 19
-transnum, 17
-version, 10, 17
-v, 10, 17, 39
allunique, 29
-best, 67
-evalue, 67
-pl, 66
-qspeedup, 66
-range, 67
-selfun, 67
-seq, 67
option category
  algorithms, 33
  direction, 34
  input, 27
  matchconstraint, 34
  matchkind, 27
  output, 37
  postprocessing, 29
ORFs, 51

P, 42
parsing
  output, 78
Patterns, 51
percentage mode, 35, 36
prefixlength, 9
prefixlength, 66
prjtab, 81
Proteinseq, 15, 45, 53

queryfiles, 27

reading frame, 25, 69
remred, 29
repeat
  branching, 25

branching tandem, 75
maximal, 25
supermaximal, 25, 27, 75
tandem, 25, 27, 75
replacement, 73
rightseq, 44
rightseq, 38

sdstab, 84
select
description, 22
number, 21
randomlength, 21
randomnum, 21
selection function, 78, 79
bundle, 79
selectmatch, 63, 68, 80
selectmatchFinaltable, 68
selectmatchHeader, 68
selectmatchFinaltable, 80
selectmatchHeader, 33, 79
selectmatchInit, 68, 80
selectmatchWrap, 68, 80
sequence
pattern, 29
SGI, 4
shared object, 79
compile, 64
Sint, 79
skipprefix, 39
skptab, 83
smallheading, 49
sort mode
ea, 37
ed, 37
ia, 37
id, 37
ida, 37
idd, 37
ja, 37
jd, 37
la, 37
ld, 37
sa, 37
sd, 37
ssptab, 83
stitab, 83
stitab1, 83
StoreMatch, 79, 80
Substring Specification, 77
suftab, 82
SUN, 4, 24, 64
swissp.sp.gz, 13, 15, 24, 56
SWISSPROT, 6
symbol mapping, 4, 7, 16, 75

tistab, 82
tolower, 30, 57
toupper, 30
TransProt11, 76

U89959, 48

vlsi, 24
vmatch, 24
VMATCHCOMMENTTOSTDOUT, 30, 70, 85
VMATCHRELATIVEINDEXPATH, 70, 85
vmatchselect, 65
vmigrate.sh, 24
vseqinfo, 17
vseqselect, 20
vstree2tex, 18
vsubseqselect, 22

wildcard, 8, 9, 38, 75, 76

X, 30
X, 30
Xdrop, 77
xml, 38, 49

Zhang, 77
