# Supplementary material for: iFISH is a publically available resource enabling versatile DNA FISH to study genome architecture
Source: Nat Commun. 2019 Apr 9;10:1636. doi: 10.1038/s41467-019-09616-w (PMC6456570; doi:10.1038/s41467-019-09616-w)
Supplement: Supplementary file 15 — Supplementary Software [file 41467_2019_9616_MOESM15_ESM.zip › Supplementary Software/source-code/iFISH-probe-design-2.0.2.zip!/iFISH-probe-design-2.0.2/ifpd/interface/views/header.tpl]

{{title}}


% if defined( 'custom\_stylesheets' ):
% for uri in custom\_stylesheets:
% end
% end
